# Palladium-catalyzed ligand-promoted site-selective cyanomethylation of unactivated C(sp^3^)–H bonds with acetonitrile†
†Electronic supplementary information (ESI) available: Experimental details including characterization data, copies of ^1^H, ^13^C NMR and NOESY spectra. See DOI: 10.1039/c5sc04066c


**DOI:** 10.1039/c5sc04066c

**Published:** 2016-01-06

**Authors:** Yongbing Liu, Ke Yang, Haibo Ge

**Affiliations:** a Department of Chemistry and Chemical Biology , Indiana University-Purdue University Indianapolis , Indianapolis , Indiana 46202 , USA . Email: geh@iupui.edu; b Institute of Chemistry and BioMedical Sciences , School of Chemistry and Chemical Engineering , Nanjing University , Nanjing 210093 , P. R. China

## Abstract

The direct cyanomethylation of unactivated sp^3^ C–H bonds of aliphatic amides was achieved *via* palladium catalysis assisted by a bidentate directing group with good functional group compatibility. This process represents the first example of the direct cross-coupling of sp^3^ C–H bonds with acetonitrile. Considering the importance of the cyano group in medicinal and synthetic organic chemistry, this reaction will find broad application in chemical research.

## Abstract 2

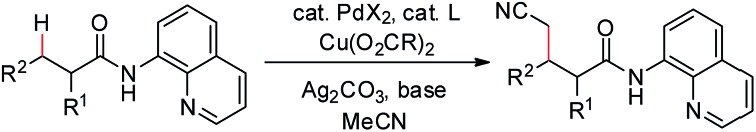
The direct coupling of unactivated sp^3^ C–H bonds in aliphatic amides with acetonitrile was achieved *via* palladium catalysis.

## Introduction

The cyanomethylation of organic molecules is of great research interest to organic and medicinal chemists due to the wide presence of the cyano group in biologically active molecules and the facile conversion of the cyano group into many other functional groups, such as amides, esters, aldehydes, and primary amines.^1^ A variety of different synthetic strategies have been developed for the selective introduction of the cyanomethyl group.^2^ Among these methods, transition metal-catalyzed cross-couplings with acetonitrile as the coupling partner^3^ have attracted considerable attention in recent years due to the avoidance of prefunctionalized substrates such as haloacetonitrile,^4^ trimethylsilylacetonitrile,^5^ cyanoacetate salts^6^ and cyanomethyltributyltin.^7^ In 2002, Culkin and Hartwig reported the first cross-coupling reaction of acetonitrile and aryl bromides *via* palladium catalysis.^8^ In another study by You and Verkade, aryl chlorides were also demonstrated as effective substrates for this transformation.^9^ Furthermore, the direct cross-coupling of benzene with acetonitrile was developed with a palladium catalyst hybridized with a titanium dioxide photocatalyst.^10^ However, to date, the direct cross-coupling of sp^3^ C–H bonds with acetonitrile has not been discovered. Considering the literature support for the Pd-catalyzed alkylation of unactivated C(sp^3^)–H bonds^11^ and the reductive elimination of dialkyl palladium(ii) species,11g–i,^12^ it is envisaged that this process should be feasible if the cyanomethyl group could effectively replace the anion of an alkyl palladium(ii) species.

## Results and discussion

In the previous reports, it was found that an alkyl arylpalladium(ii) species could be formed by the treatment of an arylpalladium(ii) species with a cyanomethyl anion in the presence of a ligand. On the basis of these results, the palladium-catalyzed direct cyanomethylation of *N*-(quinolin-8-yl)butyramide (**1a**) with acetonitrile was examined using 2,2′-bipyridine as the ligand, under basic conditions (Table 1, entry 1). Unfortunately, no desired product was observed. A copper(ii) salt was then added into the reaction system, because the copper-promoted C–H bond activation of acetonitrile^13^ and the transmetalation of an organocopper species onto an organopalladium(ii) species^14^ have been well documented. As shown in Table 1, copper carboxylates were found to be effective, with Cu(O_2_C^*n*^Pr)_2_ providing the best result (entry 3). A series of mono and bidentate ligands^15^ were then screened, and it was found out that the reaction yield was improved with 5,5′-dimethyl-2,2′-bipyridine (**L2**) (entry 6). Furthermore, the effect of the palladium catalyst was examined with Pd(OPiv)_2_ giving the optimal result (entry 9). Further optimization showed that this reaction was significantly improved with CsOPiv as the base (entry 15). In addition, the use of acetonitrile and heptane as the co-solvent could further increase the yield (entry 18). It was also noted that the reaction yield was dramatically decreased in the absence of the ligand, indicating that the ligand plays a role in stabilizing the dialkyl palladium(ii) species or the *in situ* generated Pd metal (entry 20). To our delight, the reaction yield could be further improved by increasing the load of palladium catalyst (entry 21).

**Table 1:** Optimization of the reaction conditions^*a*^

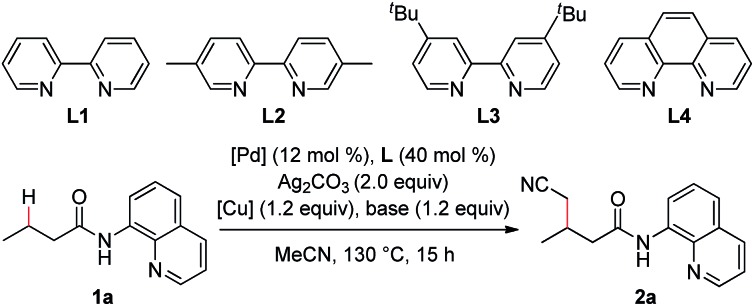					
Entry	Pd source	Cu source	Base	**L**	Yield^*b*^ (%)
1^*c*^	Pd(OAc)_2_	—	NaHMDS	**L1**	0
2	Pd(OAc)_2_	Cu(OAc)_2_	—	**L1**	11
3	Pd(OAc)_2_	Cu(O_2_C^*n*^Pr)_2_	—	**L1**	20
4	Pd(OAc)_2_	Cu(acac)_2_	—	**L1**	Trace
5	Pd(OAc)_2_	CuOAc	—	**L1**	11
6	Pd(OAc)_2_	Cu(O_2_C^*n*^Pr)_2_	—	**L2**	30
7	Pd(OAc)_2_	Cu(O_2_C^*n*^Pr)_2_	—	**L3**	23
8	Pd(OAc)_2_	Cu(O_2_C^*n*^Pr)_2_	—	**L4**	21
9	Pd(OPiv)_2_	Cu(O_2_C^*n*^Pr)_2_	—	**L2**	34
10	PdCl_2_	Cu(O_2_C^*n*^Pr)_2_	—	**L2**	22
11	Pd(MeCN)_2_Cl_2_	Cu(O_2_C^*n*^Pr)_2_	—	**L2**	26
12	Pd(OPiv)_2_	Cu(O_2_C^*n*^Pr)_2_	K_3_PO_4_	**L2**	25
13	Pd(OPiv)_2_	Cu(O_2_C^*n*^Pr)_2_	KOAc	**L2**	49
14	Pd(OPiv)_2_	Cu(O_2_C^*n*^Pr)_2_	KOPiv	**L2**	52
15	Pd(OPiv)_2_	Cu(O_2_C^*n*^Pr)_2_	CsOPiv	**L2**	56
16^*d*^	Pd(OPiv)_2_	Cu(O_2_C^*n*^Pr)_2_	CsOPiv	**L2**	69
17^*e*^	Pd(OPiv)_2_	Cu(O_2_C^*n*^Pr)_2_	CsOPiv	**L2**	73
18^*f*^	Pd(OPiv)_2_	Cu(O_2_C^*n*^Pr)_2_	CsOPiv	**L2**	76(72)^*g*^
19^*h*^	Pd(OPiv)_2_	Cu(O_2_C^*n*^Pr)_2_	CsOPiv	**L2**	68
20^*f*^	Pd(OPiv)_2_	Cu(O_2_C^*n*^Pr)_2_	CsOPiv	—	9
21^*i*^	Pd(OPiv)_2_	Cu(O_2_C^*n*^Pr)_2_	CsOPiv	**L2**	85(80)^*g*^

^*a*^Reaction conditions: **1a** (0.3 mmol), Pd source (0.036 mmol), **L** (0.12 mmol), Cu source (0.36 mmol), Ag_2_CO_3_ (0.6 mmol), base (0.36 mmol), MeCN (3.0 mL), air (1 atm), 130 °C, 15 h unless other noted.

^*b*^Yields are based on **1a**, determined by ^1^H-NMR using dibromomethane as the internal standard.

^*c*^NaHMDS (1 M in THF, 1.5 mL) was used.

^*d*^MeCN/toluene (1.5 mL/1.5 mL).

^*e*^MeCN/hexane (1.5 mL/1.5 mL).

^*f*^MeCN/heptane (1.5 mL/1.5 mL).

^*g*^Isolated yield.

^*h*^MeCN/cyclohexane (1.5 mL/1.5 mL).

^*i*^Pd(OPiv)_2_ (0.045 mmol).

With the optimized reaction conditions in hand, a substrate scope study on linear aliphatic amides was then carried out. As shown in Table 2, the direct cyanomethylation of unbranched amides provided the desired products in moderate to good yields (**2a–f**). In addition, a variety of functional groups, such as the alkenyl, chloro, ester, phenyl and thienyl groups, were well tolerated under the catalytic system, allowing for the further manipulation of the original products. Furthermore, there is an apparent steric effect for this reaction because a lower yield was obtained with substrates bearing a substituent on γ-carbon (**2g**).

**Table 2:** Scope of the linear aliphatic amides^*a*^ ^,^^*b*^

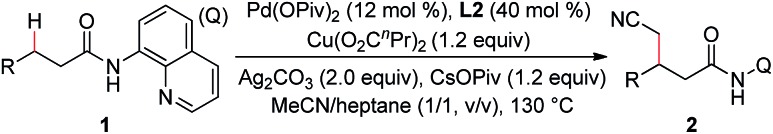
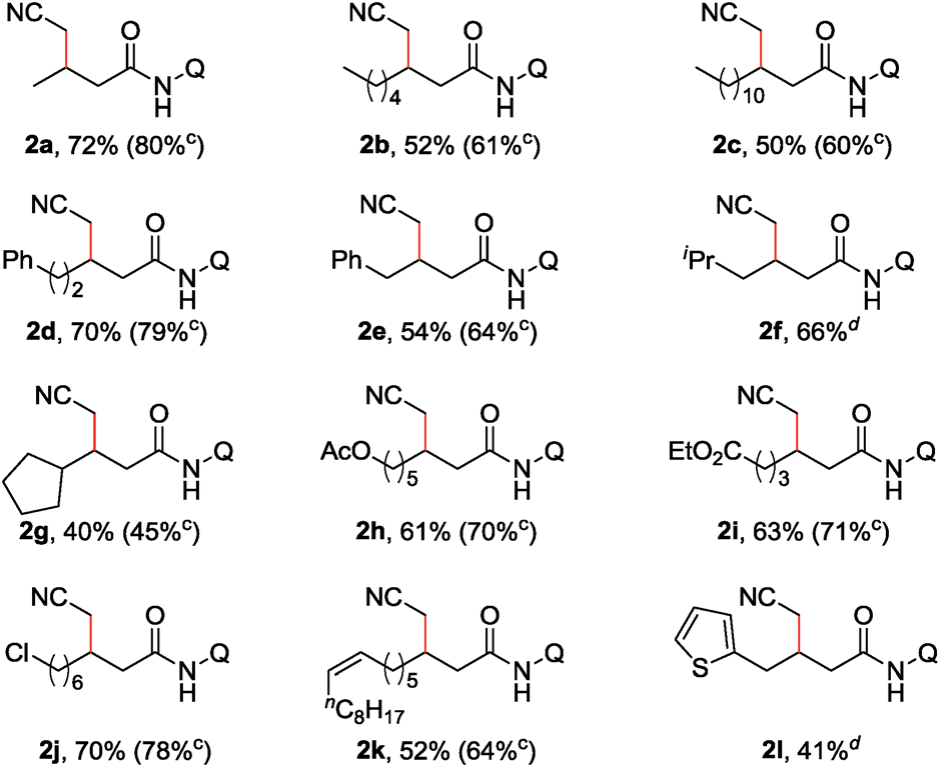

^*a*^Reaction conditions: **1** (0.3 mmol), Pd(OPiv)_2_ (0.036 mmol), **L2** (0.12 mmol), Cu(O_2_C^*n*^Pr)_2_ (0.36 mmol), Ag_2_CO_3_ (0.6 mmol), CsOPiv (0.36 mmol), MeCN (1.5 mL), heptane (1.5 mL), and air (1 atm), 130 °C, 15 h.

^*b*^Isolated yield.

^*c*^Pd(OPiv)_2_ (0.045 mmol).

^*d*^Pd(OPiv)_2_ (0.06 mmol). Q = 8-quinolinyl.

Furthermore, the scope of α-substituted aliphatic amides was studied under the modified reaction conditions (Table 3). As expected, propanamides bearing a linear, branched, or cyclic alkyl group were shown to be effective substrates (**3a–h**). It is worth mentioning that this reaction showed high site-selectivity by favouring the sp^3^ C–H bonds of the methyl group over those of the methylene groups, including that of the relatively reactive benzylic sp^3^ C–H bond (**3c**). Furthermore, the cyclic sp^3^ C–H bond could also be functionalized, albeit with a moderate yield (**3k**). Amides with α-tertiary carbon (**3l**) were inappropriate substrates and could be quantitatively recovered under current conditions.

**Table 3:** Scope of the α-substituted aliphatic amides^*a*^ ^,^^*b*^

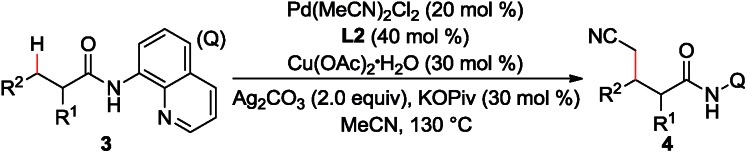
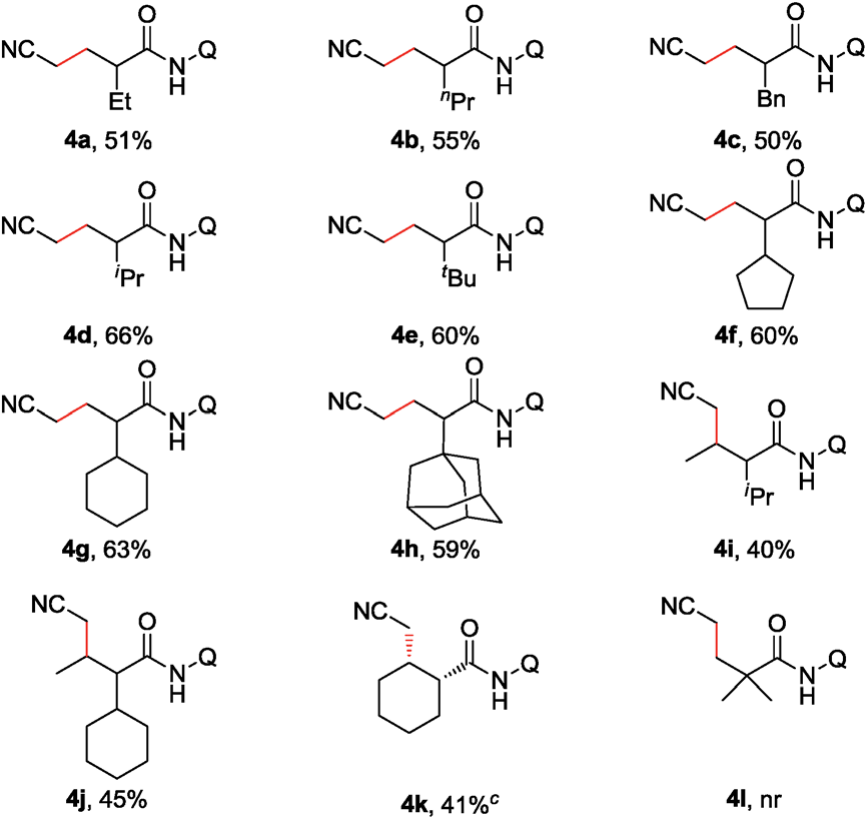

^*a*^Reaction conditions: **1** (0.3 mmol), Pd(MeCN)_2_Cl_2_ (0.06 mmol), **L2** (0.12 mmol), Cu(OAc)_2_·H_2_O (0.09 mmol), Ag_2_CO_3_ (0.6 mmol), KOPiv (0.09 mmol), MeCN (2.0 mL), air (1 atm), 130 °C, 1 h.

^*b*^Isolated yield.

^*c*^Pd(OPiv)_2_ (0.06 mmol), **L2** (0.12 mmol), Cu(O_2_C^*n*^Pr)_2_ (0.36 mmol), Ag_2_CO_3_ (0.6 mmol), CsOPiv (0.36 mmol), MeCN (1.5 mL), heptane (1.5 mL), air (1 atm), 130 °C, 15 h. Q = 8-quinolinyl.

**Scheme 1:**
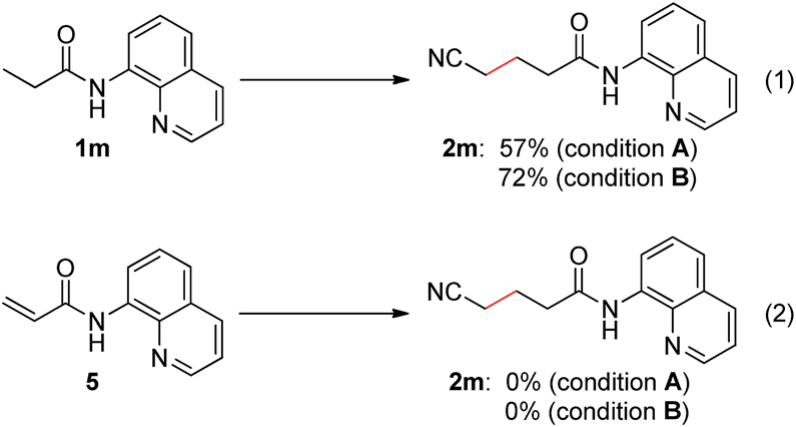
Control experiments on the reaction mechanism. Condition A: **1m** or **5** (0.3 mmol), Pd(OPiv)_2_ (0.036 mmol), **L2** (0.12 mmol), Cu(O_2_C^*n*^Pr)_2_ (0.36 mmol), Ag_2_CO_3_ (0.6 mmol), CsOPiv (0.36 mmol), MeCN (1.5 mL), heptane (1.5 mL), air (1 atm), 130 °C, 15 h. Condition B: **1m** or **5** (0.3 mmol), Pd(MeCN)_2_Cl_2_ (0.06 mmol), **L2** (0.12 mmol), Cu(OAc)_2_·H_2_O (0.09 mmol), Ag_2_CO_3_ (0.6 mmol), KOPiv (0.09 mmol), MeCN (2.0 mL), air (1 atm), 130 °C, 1 h.

To provide some insights into the catalytic cycle, we carried out mechanistic studies into this process. It has been reported that aliphatic esters and nitriles could undergo dehydrogenation to form the corresponding α,β-unsaturated derivatives.^16^ Therefore, a sequential dehydrogenation/1,4-addition process could potentially occur in this reaction and could provide the desired products. To clarify this, *N*-(quinolin-8-yl)acrylamide (**5**) was prepared and subjected to the reaction conditions (Scheme 1). It turned out that no desired product (**2m**) was obtained, and thus the dehydrogenation/1,4-addition process could be excluded.

**Scheme 2:**
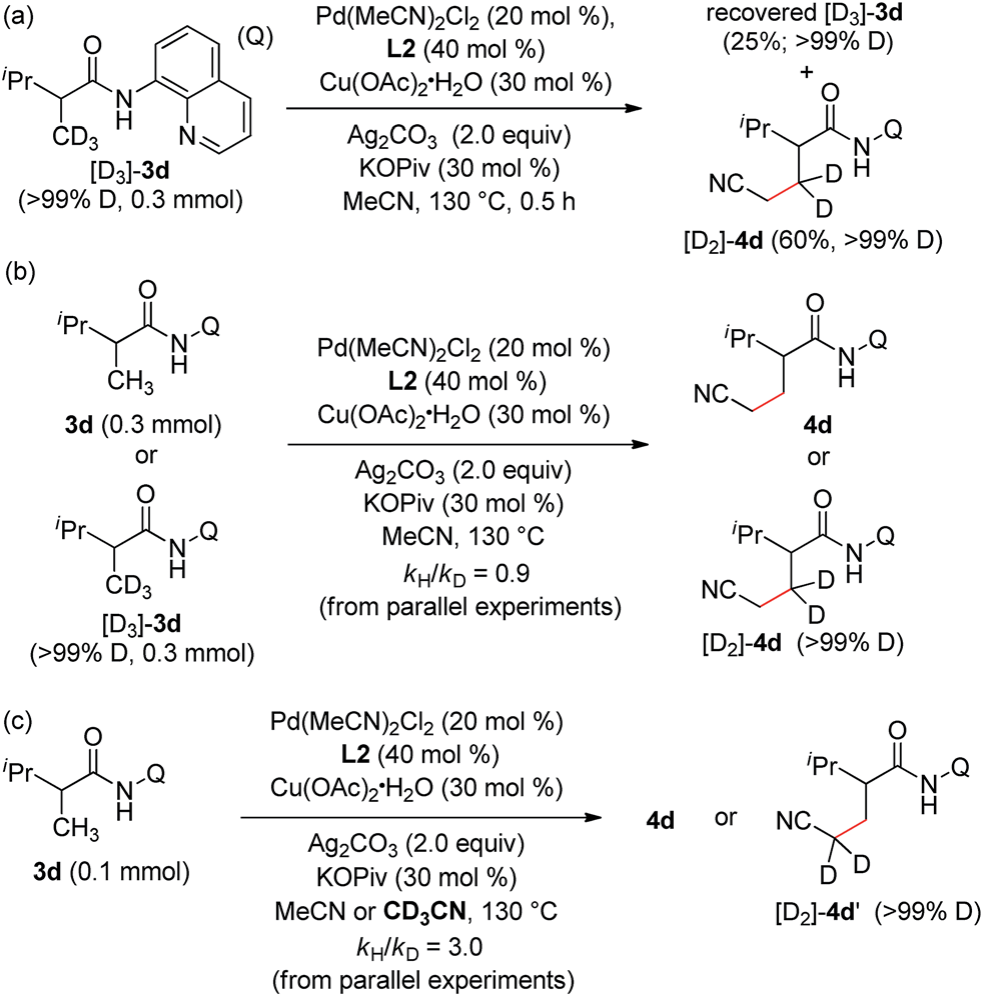
Deuterium-labelling experiments.

To further probe the reaction mechanism, a series of deuterium-labelling experiments were carried out. As shown in Scheme 2, no apparent H/D exchange was observed with deuterium-labelled 2,3-dimethyl-*N*-(quinolin-8-yl)butanamide (D_3_-**3d**) (Scheme 2a), indicating that the sp^3^ C–H bond cleavage is an irreversible step under the current reaction conditions. Furthermore, no obvious kinetic isotope effect was observed for **3d** (*vs.* D_3_-**3d**) based on the early relative rate of the parallel reactions (Scheme 2b), whereas a primary isotope effect with regard to acetonitrile (MeCN *vs.* CD_3_CN) was obtained (Scheme 2c), suggesting that the sp^3^ C–H bond cleavage of acetonitrile is the rate-limiting step in the catalytic process.

**Scheme 3:**
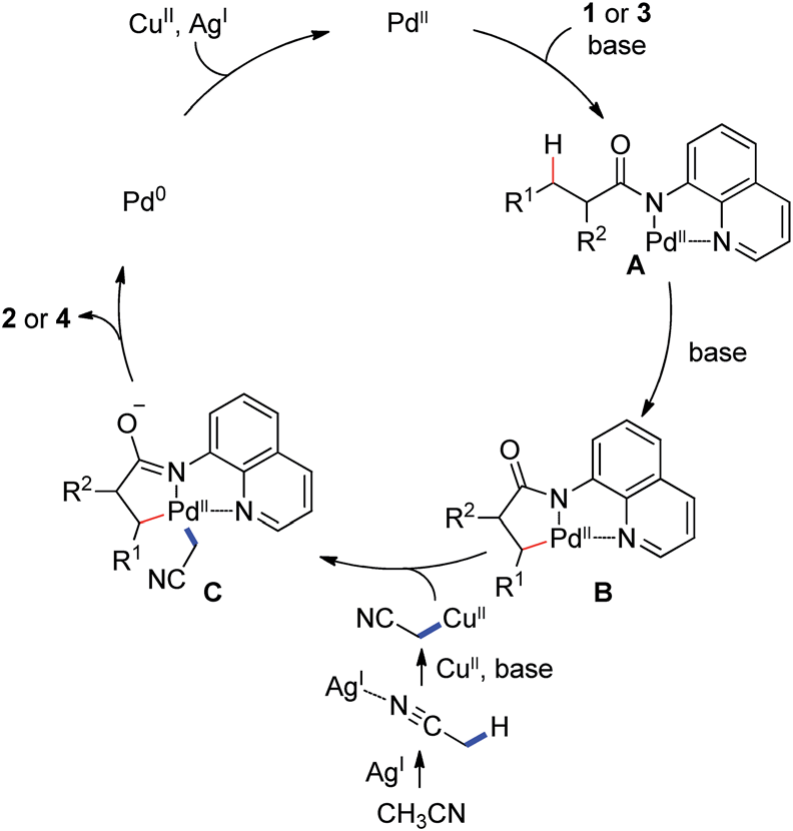
Proposed reaction mechanism.

On the basis of the abovementioned observations and the previous studies,^11^–^14^ a plausible reaction mechanism is proposed (Scheme 3), involving the coordination of amide **1** or **3** to a Pd^II^ species, followed by a ligand exchange process, giving rise to the palladium intermediate **A**. Irreversible sp^3^ C–H bond activation of this intermediate under basic conditions generates the cyclometalated palladium(ii) complex **B**. Transmetalation of the complex **B** with the cyanomethyl copper(ii) species, possibly from a silver-promoted process of acetonitrile, affords the dialkyl palladium intermediate **C**, which provides the final product **2** or **4** upon reductive elimination.

**Scheme 4:**
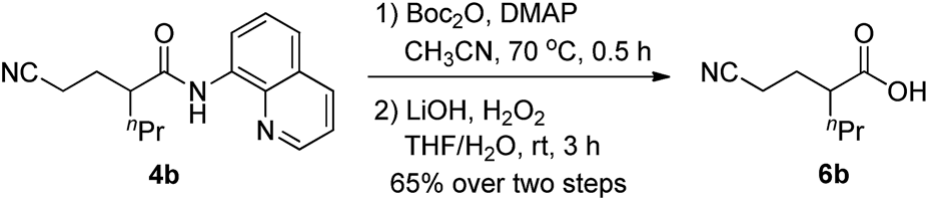
Removal of the directing group.

To further broaden the synthetic application of this methodology, removal of the 8-quinolylamino directing group of **4b** was carried out based on the reported two-step process,11*l* and the C–N bond of amide was selectively cleaved to deliver the desired acid product **6b** in a 65% yield without affecting the cyano group (Scheme 4).

## Conclusions

In summary, a highly regioselective cyanomethylation of aliphatic amides with an 8-aminoquinolinyl group as the directing moiety was developed *via* a palladium-catalyzed cross dehydrogenative coupling process. This process exhibited a predominant preference for methyl C–H bonds over methylene C–H bonds with good functional group tolerance. Mechanistic studies were carried out that excluded the possibly sequential dehydrogenation/Michael addition process. Detailed mechanistic studies of this reaction and expansion of the substrate scope^17^ are currently ongoing in our laboratory.

## Supplementary Material

Supplementary information
