# Supplementary material for: Palladium-catalyzed ligand-promoted site-selective cyanomethylation of unactivated C(sp3)–H bonds with acetonitrile
Source: Chem Sci. 2016 Jan 6;7(4):2804–8. doi: 10.1039/c5sc04066c (PMC5477045; doi:10.1039/c5sc04066c)
Supplement: Supplementary file 1 [file SC-007-C5SC04066C-s001.pdf]

Electronic Supplementary Material (ESI) for Chemical Science.
This journal is © The Royal Society of Chemistry 2016

Palladium-Catalyzed Ligand-Promoted Site-Selective Cyanomethylation of Unactivated $C(sp^3)-H$ Bonds with Acetonitrile

Yongbing Liu, Ke Yang, and Haibo Ge

Table of Contents

General Information S2

Structures of Starting Materials S2

General Procedure A for the Preparation of Starting Materials (1a-1g, 1k-1m, 3a-3h, 3k, [D$_3$]-3d and 5) S4

General Procedure B for the Preparation of Starting Materials (1h-1j, 3i and 3j) S4

Analytical Data of Starting Materials (1b, 1c, 1h-1j, 1l, 3c, 3i, 3j, [D$_3$]-3d) S5

General Procedure for Palladium-Catalyzed Site-Selective Cyanomethylation of Linear Aliphatic Amides S9

General Procedure for Palladium-Catalyzed Site-Selective Cyanomethylation of α-Substituted Aliphatic Amides S9

Analytical Data of Products S10

Experimental Date of Further Optimization Studies S20

Control Experiments on Cyanomethylation of 1a S22

Deuterium Labeling Experiment S22

Parallel KIE Experiments S24

Experimental Procedure for the Removal of the Directing Group of 4b S28

References S28

Copies of NMR Spectrum S29

General Information

^{1}H and ^{13}C NMR were recorded on a Bruker 500 MHz NMR Fourier transform spectrometer (500 MHz and 125 MHz, respectively) using tetramethylsilane as an internal reference, and chemical shifts (*δ*) and coupling constants (*J*) were expressed in ppm and Hz, respectively. Infrared spectra were obtained using a Thermo Nicolet IR 330 spectrometer. Mass (MS) analysis was obtained using Agilent 1100 series LC/MSD system with Electrospray Ionization (ESI). All the solvents and commercially available reagents were purchased from commercial sources and used directly. Starting materials **1a**-1**g**, **1k-1m**, **3a-3h**, **3k**, [D_3]-**3d** and **5** were prepared according to literature procedures.[1] **1h-1j**, **3i** and **3j** were prepared based on reported reaction protocol.[2]

Structures of Starting Materials

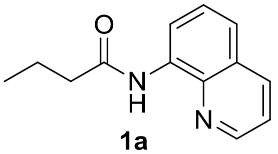

1a

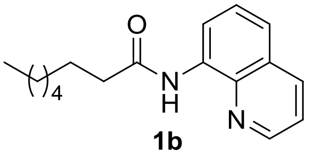

1b

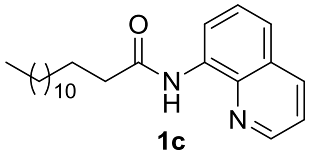

1c

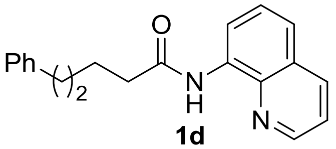

1d

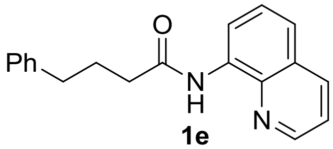

1e

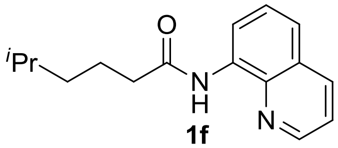

1f

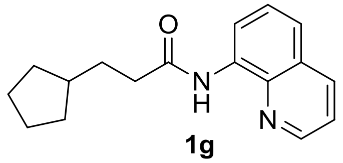

1g

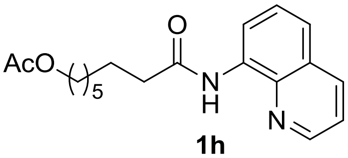

1h

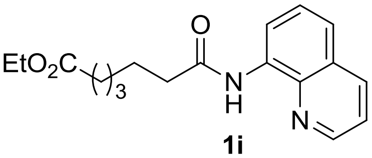

1i

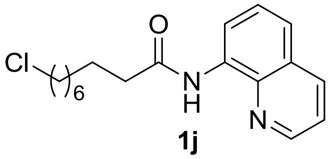

1j

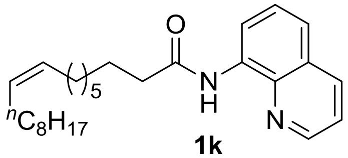

1k

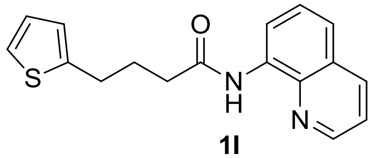

1l

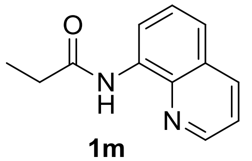

1m

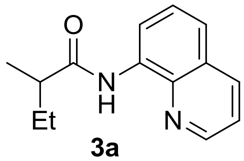

3a

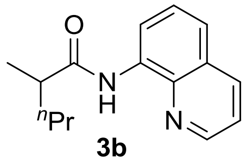

3b

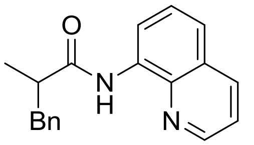

3c

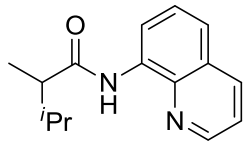

3d

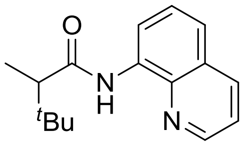

3e

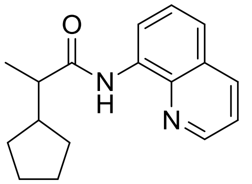

3f

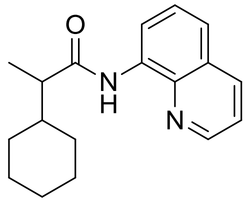

3g

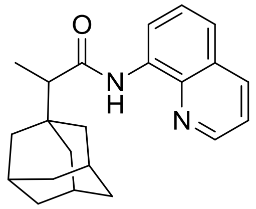

3h

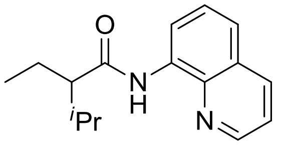

3i

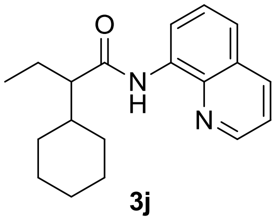

3j

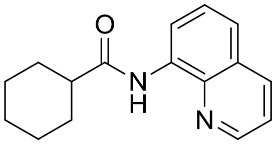

3k

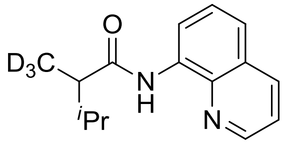

[D_3]-**3d**

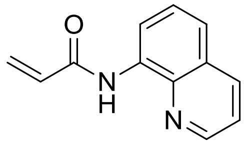

5

General Procedure A for the Preparation of Starting Materials (1a-1g, 1k-1m, 3a-3h, 3k, [D_3]-3d and 5)

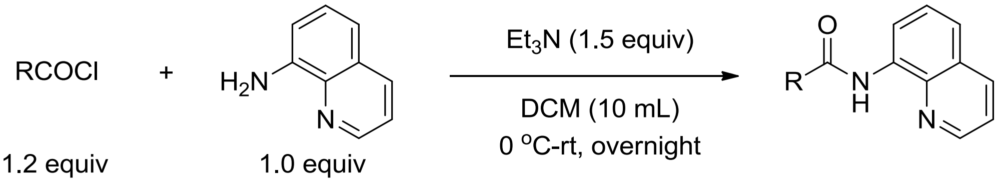

The acid chloride (6.0 mmol) was added dropwise to a solution of 8-aminoquinoline (0.721 g, 5.0 mmol) and Et_3N (1.0 mL, 7.5 mmol) in CH_2Cl_2 (10 mL) at 0 °C under nitrogen. The resulting mixture was stirred overnight at room temperature. Then the mixture was diluted with CH_2Cl_2 (10 mL), washed successively with water, saturated aqueous $NaHCO_3$, and brine. The organic layer was dried over anhydrous Na_2SO_4 and concentrated under reduced pressure. The residue was purified by flash column chromatography on silica gel eluting with EtOAc/hexanes (1/30-1/5, v/v) to afford corresponding 8-aminoquinolinyl amide.[1]

General Procedure B for the Preparation of Starting Materials (1h-1j, 3i and 3j)

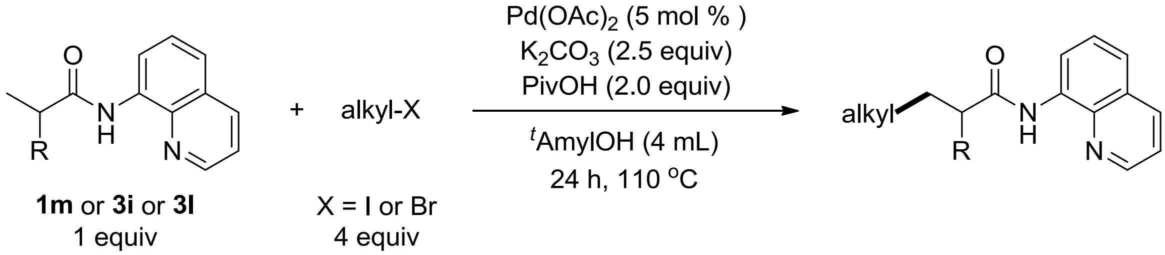

A 35 mL tube was charged with acetamide **1m** or **3i** or **3l** (601 mg, 3.0 mmol), alkyl halide (12 mmol) $Pd(OAc)_2$ (33.6 mg, 0.15 mmol), K_2CO_3 (1.037 g, 7.5 mmol), PivOH (613 mg, 6.0 mmol) and *tert*-Amyl alcohol (4.0 mL). The reaction mixture was stirred at 110 ˚C for 24 h. Then the mixture was cooled to room temperature, diluted with EtOAc (10 mL), filtered through a celite pad, and concentrated in vacuo. The residue was purified by chromatography on silica gel eluting with EtOAc/hexanes to give the desired product.[2]

Analytical Data of Starting Materials (1b, 1c, 1h-1j, 1l, 3c, 3i, 3j, [D_3]-3d)

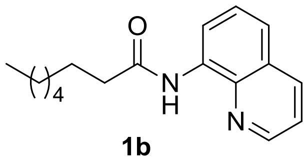

1b

Compound **1b**, pale yellow oil. ^{1}H NMR (500 MHz, $CDCl_3$) δ 0.86 (t, J = 6.9 Hz, 3H), 1.20-1.45 (m, 8H), 1.72-1.86 (m, 2H), 2.52 (t, J = 7.6 Hz, 2H), 7.38 (dd, J = 8.3, 4.2 Hz, 1H), 7.43 (dd, J = 8.2, 1.3 Hz, 1H), 7.45-7.54 (m, 1H), 8.08 (dd, J = 8.3, 1.6 Hz, 1H), 8.75 (dd, J = 4.2, 1.6 Hz, 1H), 8.78 (dd, J = 7.6, 1.2 Hz, 1H), 9.78 (brs, 1H); ^{13}C NMR (125 MHz, $CDCl_3$) δ 14.1, 22.6, 25.7, 29.1, 29.3, 31.7, 38.3, 116.4, 121.3, 121.5, 127.4, 127.9, 134.6, 136.3, 138.3, 148.1, 171.9; IR (neat) v 3356, 3044, 2953, 2927, 2854, 1689, 1525, 1486, 1424, 1386, 1325, 1163, 826, 791 cm^{-1}; Ms (ESI): m/z = 271.2 $[M+H]^+$.

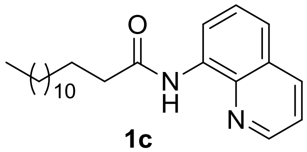

1c

Compound **1c**, white solid. ^{1}H NMR (500 MHz, $CDCl_3$) δ 0.86 (t, J = 7.0 Hz, 3H), 1.18-1.44 (m, 20H), 1.77-1.85 (m, 2H), 2.52 (t, J = 7.5 Hz, 2H), 7.37 (dd, J = 8.2, 4.2 Hz, 1H), 7.41 (dd, J = 8.3, 1.3 Hz, 1H), 7.44-7.51 (m, 1H), 8.07 (dd, J = 8.3, 1.7 Hz, 1H), 8.74 (dd, J = 4.2, 1.7 Hz, 1H), 8.78 (dd, J = 7.6, 1.1 Hz, 1H), 9.78 (brs, 1H); ^{13}C NMR (125 MHz, $CDCl_3$) δ 14.1, 22.7, 25.7, 29.33, 29.38, 29.44, 29.53, 29.65, 29.67, 29.70, 31.9, 38.2, 116.4, 121.3, 121.5, 127.4, 127.9, 134.6, 136.3, 138.3, 148.0, 171.8; IR (neat) v 3358, 3048, 2959, 2852, 1697, 1521, 1486, 1424, 1386, 1325, 1165, 826, 791, 679 cm^{-1}; Ms (ESI): m/z = 355.2 $[M+H]^+$.

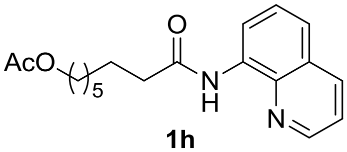

1h

Compound **1h**, colorless oil. ^{1}H NMR (500 MHz, $CDCl_3$) δ 1.33-1.49 (m, 6H), 1.57-1.71 (m, 2H), 1.78-1.88 (m, 2H), 2.03 (s, 3H), 2.56 (t, J = 7.5 Hz, 2H), 4.05 (t,

J = 6.7 Hz, 2H), 7.45 (dd, *J* = 8.3, 4.3 Hz, 1H), 7.49 (dd, *J* = 8.2, 1.3 Hz, 1H), 7.51-7.56 (m, 1H), 8.16 (dd, *J* = 8.3, 1.5 Hz, 1H), 8.73-8.84 (m, 2H), 9.80 (brs, 1H); ^{13}C NMR (125 MHz, $CDCl_3$) δ 21.1, 25.7, 25.9, 28.7, 29.2, 29.3, 38.3, 64.7, 116.6, 121.5, 121.7, 127.6, 128.1, 134.7, 136.5, 138.5, 148.2, 171.4, 171.9; IR (neat) ν 3356, 3047, 2933, 2857, 1735, 1689, 1525, 1486, 1425, 1386, 1325, 1242, 1164, 1041, 827, 793, 680 cm^{-1}; Ms (ESI): *m*/*z* = 329.1 $[M+H]^+$.

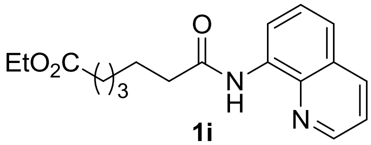

1i

Compound **1i**, pale yellow solid. ^{1}H NMR (500 MHz, $CDCl_3$) δ 1.20 (t, *J* = 7.1 Hz, 3H), 1.37-1.49 (m, 2H), 1.60-1.72 (m, 2H), 1.73-1.85 (m, 2H), 2.28 (t, *J* = 7.5 Hz, 2H), 2.52 (t, *J* = 7.5 Hz, 2H), 4.08 (q, *J* = 7.1 Hz, 2H), 7.38 (dd, *J* = 8.2, 4.2 Hz, 1H), 7.42 (dd, *J* = 8.2, 1.3 Hz, 1H), 7.44-7.53 (m, 1H), 8.08 (dd, *J* = 8.3, 1.6 Hz, 1H), 8.63-8.80 (m, 2H), 9.76 (brs, 1H); ^{13}C NMR (125 MHz, $CDCl_3$) δ 14.3, 24.7, 25.3, 28.7, 34.2, 37.9, 60.2, 116.4, 121.4, 121.6, 127.4, 127.9, 134.5, 136.3, 138.3, 148.1, 171.5, 173.6; IR (neat) ν 3354, 2978, 2937, 2865, 1732, 1684, 1524, 1486, 1387, 1163, 1031, 829, 795 cm^{-1}; Ms (ESI): *m*/*z* = 315.1 $[M+H]^+$.

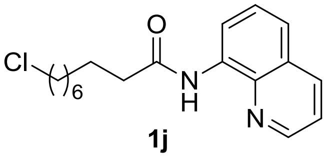

1j

Compound **1j**, pale yellow solid. ^{1}H NMR (500 MHz, $CDCl_3$) δ 1.30-1.45 (m, 8H), 1.68-1.87 (m, 4H), 2.55 (t, *J* = 7.5 Hz, 2H), 3.51 (t, *J* = 6.8 Hz, 2H), 7.44 (dd, *J* = 8.1, 4.1 Hz, 1H), 7.46-7.56 (m, 2H), 8.14 (d, *J* = 8.2 Hz, 1H), 8.69-8.85 (m, 2H), 9.80 (brs, 1H); ^{13}C NMR (125 MHz, $CDCl_3$) δ 25.7, 26.9, 28.8, 29.25, 29.31, 32.7, 38.3, 45.2, 116.5, 121.4, 121.7, 127.5, 128.0, 134.6, 136.5, 138.4, 148.2, 171.9; IR (neat) ν 3355, 2930, 1688, 1525, 1485, 1424, 1385, 1325, 1260, 1166, 826, 792, 758, 680 cm^{-1}; Ms (ESI): *m*/*z* = 319.2 $[M+H]^+$.

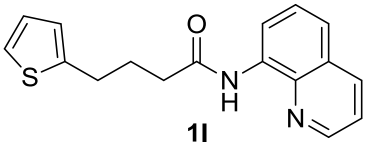

1l

Compound **1l**, pale yellow solid. ^{1}H NMR (500 MHz, $CDCl_3$) δ 2.13-2.24 (m, 2H), 2.63 (t, *J* = 7.4 Hz, 2H), 3.00 (t, *J* = 7.3 Hz, 2H), 6.78-6.89 (m, 1H), 6.94 (dd, *J* = 5.1, 3.4 Hz, 1H), 7.14 (dd, *J* = 5.1, 1.2 Hz, 1H), 7.46 (dd, *J* = 8.2, 4.2 Hz, 1H), 7.50 (dd, *J* = 8.3, 1.5 Hz, 1H), 7.52-7.63 (m, 1H), 8.16 (dd, *J* = 8.3, 1.6 Hz, 1H), 8.65-8.90 (m, 2H), 9.80 (brs, 1H); ^{13}C NMR (125 MHz, $CDCl_3$) δ 27.5, 29.3, 37.1, 116.6, 121.5, 121.7, 123.4, 124.8, 126.9, 127.5, 128.0, 134.6, 136.5, 138.4, 144.4, 148.2, 171.3; IR (neat) ν 3351, 3066, 2934, 2851, 1685, 1522, 1485, 1424, 1385, 1259, 1163, 826, 792, 696 cm^{-1}; Ms (ESI): *m*/*z* = 297.1 $[M+H]^{+}$.

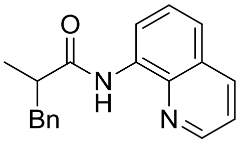

3c

Compound **3c**, colorless oil. ^{1}H NMR (500 MHz, $CDCl_3$) δ 1.32 (d, *J* = 6.8 Hz, 3H), 2.79 (dd, *J* = 13.3, 7.4 Hz, 1H), 2.82-2.92 (m, 1H), 3.20 (dd, *J* = 13.3, 6.9 Hz, 1H), 7.07-7.15 (m, 1H), 7.15-7.25 (m, 4H), 7.29 (dd, *J* = 8.1, 4.1 Hz, 1H), 7.37 (d, *J* = 8.2 Hz, 1H), 7.40-7.51 (m, 1H), 7.98 (d, *J* = 8.2 Hz, 1H), 8.60-8.72 (m, 1H), 8.73-8.87 (m, 1H), 9.77 (brs, 1H); ^{13}C NMR (125 MHz, $CDCl_3$) δ 17.7, 40.2, 44.7, 116.3, 121.3, 121.4, 126.2, 127.2, 127.7, 128.3, 129.0, 134.4, 136.1, 138.2, 139.5, 147.9, 174.3; IR (neat) ν 3354, 3027, 2969, 2931, 1684, 1522, 1485, 1454, 1387, 1324, 1240, 1161, 1079, 912, 856, 792 cm^{-1}; Ms (ESI): *m*/*z* = 291.1 $[M+H]^{+}$.

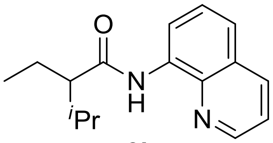

3i

Compound **3i**, colorless oil. ^{1}H NMR (500 MHz, $CDCl_3$) δ 0.99 (t, *J* =7.4 Hz, 3H), 1.03 (d, *J* = 6.7 Hz, 3H), 1.05 (d, *J* = 6.7 Hz, 3H), 1.68-1.84 (m, 2H), 1.95-2.06 (m, 1H), 2.08-2.15 (m, 1H), 7.44 (dd, *J* = 8.3, 4.2 Hz, 1H), 7.48 (dd, *J* = 8.2, 1.4 Hz, 1H), 7.52-7.57 (m, 1H), 8.15 (dd, *J* = 8.3, 1.6 Hz, 1H), 8.81 (dd, *J* = 4.3, 1.7 Hz,

1H), 8.85 (dd, *J* = 7.5, 1.4 Hz, 1H), 9.82 (brs, 1H); ^{13}C NMR (125 MHz, $CDCl_3$) δ 14.5, 20.6, 21.1, 23.5, 31.1, 58.4, 116.5, 121.4, 121.6, 127.6, 128.1, 134.6, 136.4, 138.6, 148.3, 174.5; IR (neat) ν 3359, 2962, 2932, 2873, 1684, 1525, 1485, 1424, 1386, 1324, 1172, 826, 792, 757, 669, 603 cm^{-1}; Ms (ESI): *m*/*z* = 257.1 $[M+H]^+$.

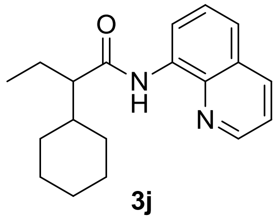

3j

Compound **3j**, colorless oil. ^{1}H NMR (500 MHz, $CDCl_3$) δ 0.98 (t, *J* =7.4 Hz, 3H), 1.02-1.32 (m, 5H), 1.59-1.84 (m, 7H), 1.87-1.97 (m, 1H), 2.09-2.19 (m, 1H), 7.42 (dd, *J* = 8.1, 4.1 Hz, 1H), 7.47 (d, *J* = 8.0, 1H), 7.49-7.57 (m, 1H), 8.12 (d, *J* = 8.2 Hz, 1H), 8.80 (d, *J* = 3.1 Hz, 1H), 8.86 (dd, *J* = 7.4, 0.8 Hz, 1H), 9.82 (brs, 1H); ^{13}C NMR (125 MHz, $CDCl_3$) δ 12.4, 23.1, 26.4, 26.50, 26.53, 31.0, 31.4, 40.6, 57.5, 116.5, 121.4, 121.6, 127.5, 128.0, 134.5, 136.4, 138.5, 148.2, 174.5; IR (neat) ν 3359, 2927, 2851, 1684, 1524, 1485, 1424, 1384, 1323, 1259, 1165, 826, 792, 757, 676 cm^{-1}; Ms (ESI): *m*/*z* = 297.1 $[M+H]^+$.

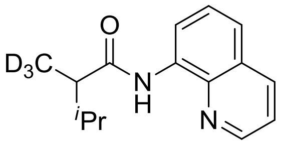

[D_3]-**3d**

Compound [D_3]-**3d**, white solid. ^{1}H NMR (500 MHz, $CDCl_3$) δ 1.02 (d, *J* =6.8 Hz, 3H), 1.04 (d, *J* =6.8 Hz, 3H), 1.97-2.11 (m, 1H), 2.32 (d, *J* =7.9 Hz, 1H), 7.44 (dd, *J* = 8.3, 4.2 Hz, 1H), 7.48 (dd, *J* = 8.2, 1.3 Hz, 1H), 7.50-7.59 (m, 1H), 8.07-8.20 (m, 1H), 8.71-8.88 (m, 2H), 9.83 (brs, 1H); ^{13}C NMR (125 MHz, $CDCl_3$) δ 19.7, 21.3, 31.8, 49.8, 116.5, 121.4, 121.7, 127.6, 128.1, 134.7, 136.5, 138.6, 148.3, 175.3; IR (neat) ν 3357, 2961, 2872, 1685, 1524, 1485, 1424, 1385, 1324, 1168, 826, 791 cm^{-1}; Ms (ESI): *m*/*z* = 246.1 $[M+H]^+$.

General Procedure for Palladium-Catalyzed Site-Selective Cyanomethylation of Linear Aliphatic Amides

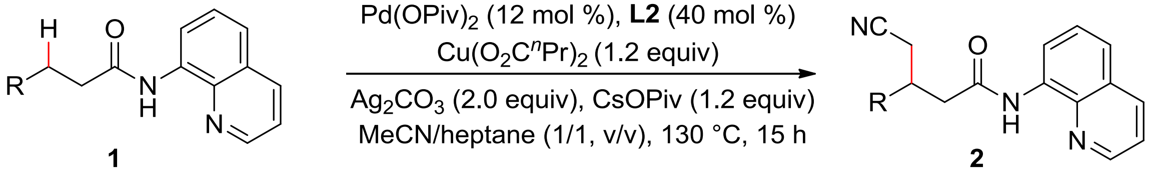

A 35 mL sealed tube was charged with amide **1** (0.3 mmol), $Pd(OPiv)_2$ (11.1 mg, 0.036 mmol), 5,5'-dimethyl-2,2'-bipyridine (**L2**) (22.1 mg, 0.12 mmol), $Cu(O_2C^nPr)_2$ (85.6 mg, 0.36 mmol), Ag_2CO_3 (165.5 mg, 0.6 mmol), CsOPiv (84.3 mg, 0.36 mmol), MeCN (1.5 mL) and heptane (1.5 mL). After sealed, the reaction mixture was stirred at 130 ˚C for 15 h. Then the mixture was cooled to room temperature, quenched with aqueous NaOH (1 M, 2 mL), and extracted with EtOAc (3×10 mL). The combined organic phase was dried over anhydrous Na_2SO_4 and concentrated under reduced pressure. The residue was purified by chromatography on silica gel eluting with EtOAc/hexanes (1/10-1/3, v/v) to provide the desired product **2**.

General Procedure for Palladium-Catalyzed Site-Selective Cyanomethylation of α-Substituted Aliphatic Amides

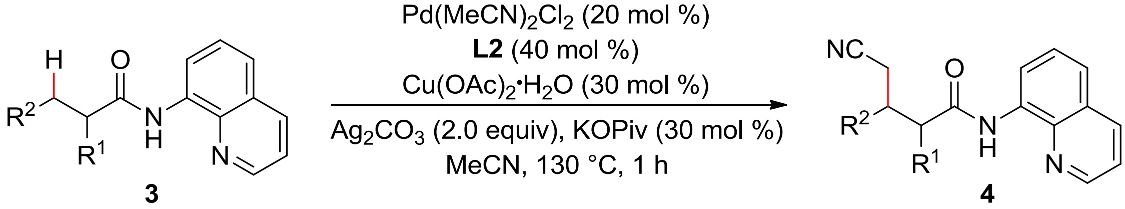

A 35 mL sealed tube was charged with amide **3** (0.3 mmol), $Pd(MeCN)_2Cl_2$ (15.6 mg, 0.06 mmol), 5,5'-dimethyl-2,2'-bipyridine (**L2**) (22.1 mg, 0.12 mmol), $Cu(OAc)_2{\cdot}H_2O$ (18.0 mg, 0.09 mmol), Ag_2CO_3 (165.5 mg, 0.6 mmol), KOPiv (12.6 mg, 0.09 mmol), and MeCN (2.0 mL). After sealed, the reaction mixture was stirred at 130 ˚C for 1 h. Then the mixture was cooled to room temperature, quenched with aqueous NaOH (1 M, 2 mL), and extracted with EtOAc (3×10 mL). The combined organic phase was dried over anhydrous Na_2SO_4 and concentrated under reduced pressure. The residue was purified by chromatography on silica gel eluting with EtOAc/hexanes (1/15-1/6, v/v) to provide the desired product **4**.

Analytical Data of Products

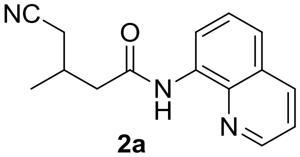

2a

Compound **2a**, colorless oil, yield: 72%. ^{1}H NMR (500 MHz, $CDCl_3$) δ 1.25 (d, J = 6.0 Hz, 3H), 2.53-2.72 (m, 5H), 7.47 (dd, J = 8.3, 4.2 Hz, 1H), 7.50-7.58 (m, 2H), 8.11-8.23 (m, 1H), 8.67-8.77 (m, 1H), 8.77-8.87 (m, 1H), 9.85 (brs, 1H); ^{13}C NMR (125 MHz, $CDCl_3$) δ 19.6, 24.2, 27.9, 43.4, 116.7, 118.4, 121.9, 122.0, 127.5, 128.1, 134.3, 136.5, 138.4, 148.4, 169.3; IR (neat) ν 3347, 3049, 2964, 2931, 2245, 1684, 1527, 1486, 1423, 1327, 1166, 827, 793, 759, 685 cm^{-1}; Ms (ESI): m/z = 254.1 [M+H]$^+$.

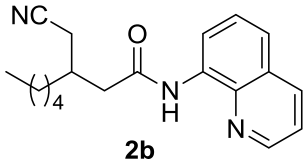

2b

Compound **2b**, colorless oil, yield: 52%. ^{1}H NMR (500 MHz, $CDCl_3$) δ 0.89 (t, J = 7.0 Hz, 3H), 1.27-1.48 (m, 6H), 1.51-1.61 (m, 2H), 2.39-2.50 (m, 1H), 2.57-2.67 (m, 3H), 2.73 (dd, J = 15.4, 5.6 Hz, 1H), 7.46 (dd, J = 8.3, 4.2 Hz, 1H), 7.49-7.57 (m, 2H), 8.17 (dd, J = 8.3, 1.6 Hz, 1H), 8.67-8.77 (m, 1H), 8.81 (dd, J = 4.2, 1.6 Hz, 1H), 9.87 (brs, 1H); ^{13}C NMR (125 MHz, $CDCl_3$) δ 14.1, 21.8, 22.6, 26.5, 31.8, 32.3, 33.4, 41.4, 116.7, 118.5, 121.8, 121.9, 127.5, 128.1, 134.3, 136.5, 138.4, 148.4, 169.5; IR (neat) ν 3347, 2955, 2929, 2857, 2244, 1685, 1527, 1489, 1425, 1386, 1326, 1164, 827, 793, 758, 685 cm^{-1}; Ms (ESI): m/z = 310.2 [M+H]$^+$.

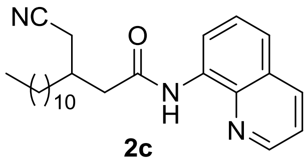

2c

Compound **2c**, white solid, yield: 50%. ^{1}H NMR (500 MHz, $CDCl_3$) δ 0.88 (t, J = 7.0 Hz, 3H), 1.17-1.44 (m, 18H), 1.50-1.61 (m, 2H), 2.38-2.51 (m, 1H), 2.55-2.68 (m, 3H), 2.73 (dd, J = 15.4, 5.6 Hz, 1H), 7.46 (dd, J = 8.3, 4.2 Hz, 1H), 7.49-7.57 (m, 2H), 8.16 (dd, J = 8.2, 1.6 Hz, 1H), 8.67-8.77 (m, 1H), 8.81 (dd, J = 4.2, 1.7 Hz, 1H), 9.87 (brs, 1H); ^{13}C NMR (125 MHz, $CDCl_3$) δ 14.2, 21.8, 22.8, 26.9, 29.5,

29.60, 29.61, 29.67, 29.73, 29.74, 32.0, 32.3, 33.5, 41.5, 116.6, 118.5, 121.8, 121.9, 127.4, 128.1, 134.3, 136.5, 138.4, 148.4, 169.5; IR (neat) *v* 3342, 3044, 2925, 2854, 2246, 1685, 1527, 1486, 1465, 1425, 1388, 1326, 1164, 827, 792, 757 cm^{-1}; Ms (ESI): *m*/*z* = 394.2 $[M+H]^+$.

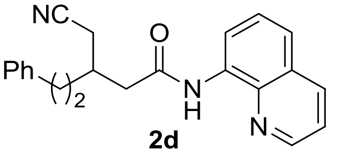

2d

Compound **2d**, white solid, yield: 70%. ^1H NMR (500 MHz, $CDCl_3$) δ 1.85-2.00 (m, 2H), 2.44-2.56 (m, 1H), 2.60-2.85 (m, 6H), 7.15-7.25 (m, 3H), 7.26-7.32 (m, 2H), 7.47 (dd, *J* = 8.2, 4.2 Hz, 1H), 7.50-7.59 (m, 2H), 8.17 (dd, *J* = 8.3, 1.6 Hz, 1H), 8.67-8.77 (m, 1H), 8.82 (dd, *J* = 4.2, 1.6 Hz, 1H), 9.88 (brs, 1H); ^{13}C NMR (125 MHz, $CDCl_3$) δ 21.8, 31.9, 33.2, 35.1, 41.3, 116.7, 118.3, 121.9, 122.0, 126.3, 127.4, 128.1, 128.4, 128.7, 134.2, 136.5, 138.4, 141.0, 148.4, 169.2; IR (neat) *v* 3345, 3055, 3026, 2927, 2858, 2244, 1684, 1526, 1486, 1425, 1388, 1326, 1166, 827, 793, 754 cm^{-1}; Ms (ESI): *m*/*z* = 344.1 $[M+H]^+$.

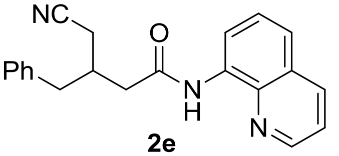

2e

Compound **2e**, white solid, yield: 54%. ^1H NMR (500 MHz, $CDCl_3$) δ 2.47 (dd, *J* = 16.9, 4.1 Hz, 1H), 2.58 (dd, *J* = 17.0, 4.3 Hz, 1H), 2.65-2.83 (m, 4H), 2.87-2.98 (m, 1H), 7.20-7.28 (m, 3H), 7.28-7.35 (m, 2H), 7.46 (dd, *J* = 8.3, 4.3 Hz, 1H), 7.49-7.59 (m, 2H), 8.16 (dd, *J* = 8.3, 1.6 Hz, 1H), 8.67-8.77 (m, 1H), 8.81 (dd, *J* = 4.3, 1.7 Hz, 1H), 9.86 (brs, 1H); ^{13}C NMR (125 MHz, $CDCl_3$) δ 21.3, 34.2, 39.5, 40.8, 116.7, 118.3, 121.9, 122.0, 127.0, 127.4, 128.1, 128.9, 129.3, 134.3, 136.5, 138.3, 138.4, 148.4, 169.2; IR (neat) *v* 3346, 3060, 3022, 2925, 2851, 2246, 1684, 1527, 1485, 1424, 1325, 1261, 1160, 827, 792, 748, 702 cm^{-1}; Ms (ESI): *m*/*z* = 330.1 $[M+H]^+$.

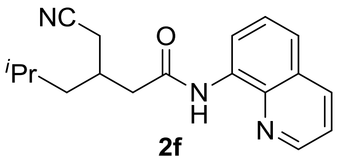

2f

Compound **2f**, colorless oil, yield: 66%. ^{1}H NMR (500 MHz, $CDCl_3$) δ 0.95 (d, J = 6.8 Hz, 3H), 0.96 (d, J = 6.8 Hz, 3H), 1.35-1.51 (m, 2H), 1.64-1.76 (m, 1H), 2.48-2.75 (m, 5H), 7.46 (dd, J = 8.3, 4.3 Hz, 1H), 7.49-7.59 (m, 2H), 8.10-8.20 (m, 1H), 8.68-8.77 (m, 1H), 8.77-8.85 (m, 1H), 9.87 (brs, 1H); ^{13}C NMR (125 MHz, $CDCl_3$) δ 21.9, 22.3, 22.9, 25.2, 29.9, 41.4, 42.6, 116.6, 118.4, 121.8, 121.9, 127.4, 128.0, 134.2, 136.5, 138.3, 148.4, 169.4; IR (neat) v 3347, 2957, 2870, 2244, 1684, 1527, 1486, 1425, 1326, 1166, 827, 793, 758, 684 cm^{-1}; Ms (ESI): m/z = 296.1 $[M+H]^+$.

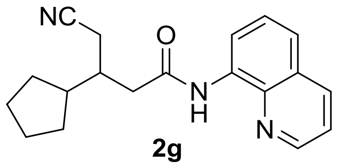

2g

Compound **2g**, colorless oil, yield: 40%. ^{1}H NMR (500 MHz, $CDCl_3$) δ 1.15-1.30 (m, 2H), 1.57-1.73 (m, 4H), 1.87-2.07 (m, 3H), 2.14-2.31 (m, 1H), 2.59-2.70 (m, 2H), 2.74 (dd, J = 17.1, 4.6 Hz, 1H), 2.87 (dd, J = 15.6, 3.9 Hz, 1H), 7.47 (dd, J = 8.2, 4.2 Hz, 1H), 7.50-7.60 (m, 2H), 8.17 (dd, J = 8.3, 1.7 Hz, 1H), 8.68-8.77 (m, 1H), 8.82 (dd, J = 4.3, 1.7 Hz, 1H), 9.89 (brs, 1H); ^{13}C NMR (125 MHz, $CDCl_3$) δ 21.4, 25.34, 25.37, 30.9, 31.1, 37.7, 40.3, 43.4, 116.7, 118.7, 121.86, 121.92, 127.5, 128.1, 134.4, 136.5, 138.4, 148.5, 169.8; IR (neat) v 3346, 2951, 2868, 2238, 1685, 1527, 1485, 1425, 1388, 1326, 827, 793 cm^{-1}; Ms (ESI): m/z = 308.3 $[M+H]^+$.

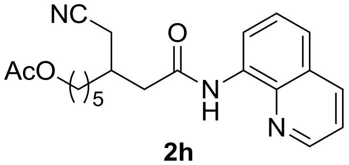

2h

Compound **2h**, pale yellow oil, yield: 61%. ^{1}H NMR (500 MHz, $CDCl_3$) δ 1.35-1.46 (m, 4H), 1.55-1.67 (m, 4H), 2.04 (s, 3H), 2.40-2.51 (m, 1H), 2.57-2.67 (m, 2H), 2.69 (dd, J = 15.6, 8.1 Hz, 1H), 2.77 (dd, J = 15.6, 5.6 Hz, 1H), 4.05 (t, J = 6.6 Hz, 2H), 7.54-7.66 (m, 3H), 8.33 (dd, J = 8.2, 1.5 Hz, 1H), 8.64-8.75 (m, 1H), 8.93 (dd, J = 4.4, 1.5 Hz, 1H), 9.94 (brs, 1H); ^{13}C NMR (125 MHz, $CDCl_3$) δ 21.0, 21.8, 25.9, 26.4, 28.5, 32.2, 33.3, 41.0, 64.4, 118.5, 119.9, 121.8, 122.8, 128.0,

128.4, 133.0, 136.8, 139.0, 147.6, 170.0, 171.1; IR (neat) *ν* 3345, 2933, 2859, 2241, 1734, 1684, 1527, 1486, 1425, 1387, 1325, 1243, 1163, 1135, 1043, 828, 794, 761 cm^{-1}; Ms (ESI): *m*/*z* = 368.1 $[M+H]^+$.

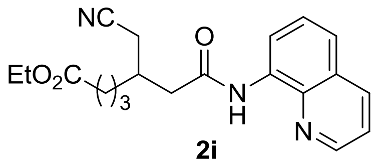

2i

Compound **2i**, pale yellow oil, yield: 63%. ^1H NMR (500 MHz, $CDCl_3$) *δ* 1.23 (t, *J* = 7.1 Hz, 3H), 1.54-1.64 (m, 2H), 1.65-1.77 (m, 2H), 2.33 (t, *J* = 7.2 Hz, 2H), 2.40-2.50 (m, 1H), 2.57-2.67 (m, 3H), 2.73 (dd, *J* = 15.5, 5.4 Hz, 1H), 4.11 (q, *J* = 7.1 Hz, 2H), 7.41-7.46 (m, 1H), 7.47-7.54 (m, 2H), 8.11-8.17 (m, 1H), 8.66-8.73 (m, 1H), 8.75-8.83 (m, 1H), 9.85 (brs, 1H); ^{13}C NMR (125 MHz, $CDCl_3$) *δ* 14.3, 21.7, 22.1, 32.0, 32.8, 34.0, 41.0, 60.5, 116.6, 118.2, 121.8, 121.9, 127.3, 128.0, 134.2, 136.4, 138.3, 148.4, 169.2, 173.2; IR (neat) *ν* 3346, 2981, 2936, 2869, 2245, 1732, 1684, 1526, 1486, 1425, 1326, 1181, 1030, 953, 828, 794, 761 cm^{-1}; Ms (ESI): *m*/*z* = 354.1 $[M+H]^+$.

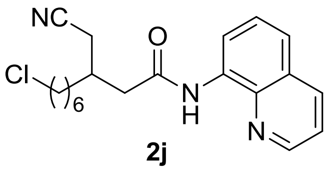

2j

Compound **2j**, pale yellow solid, yield: 70%. ^1H NMR (500 MHz, $CDCl_3$) *δ* 1.31-1.50 (m, 6H), 1.53-1.62 (m, 2H), 1.70-1.82 (m, 2H), 2.38-2.51 (m, 1H), 2.56-2.68 (m, 3H), 2.73 (dd, *J* = 15.4, 5.7 Hz, 1H), 3.51 (t, *J* = 6.7 Hz, 2H), 7.47 (dd, *J* = 8.2, 4.2 Hz, 1H), 7.50-7.62 (m, 2H), 8.17 (dd, *J* = 8.3, 1.6 Hz, 1H), 8.64-8.77 (m, 1H), 8.81 (dd, *J* = 4.1, 1.5 Hz, 1H), 9.87 (brs, 1H); ^{13}C NMR (125 MHz, $CDCl_3$) *δ* 21.9, 26.75, 26.84, 28.9, 32.3, 32.6, 33.4, 41.4, 45.1, 116.7, 118.4, 121.9, 122.0, 127.5, 128.1, 134.3, 136.5, 138.4, 148.4, 169.4; IR (neat) *ν* 3344, 2931, 2856, 2244, 1684, 1523, 1425, 1425, 1387, 1324, 1162, 826, 792, 758, 641 cm^{-1}; Ms (ESI): *m*/*z* = 358.2 $[M+H]^+$.

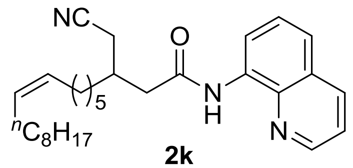

2k

Compound **2k**, colorless oil, yield: 52%. ^{1}H NMR (500 MHz, $CDCl_3$) δ 0.85 (t, *J* = 7.0 Hz, 3H), 1.20-1.41 (m, 18H), 1.51-1.62 (m, 2H), 1.85-2.10 (m, 4H), 2.39-2.49 (m, 1H), 2.55-2.68 (m, 3H), 2.73 (dd, *J* = 15.5, 5.6 Hz, 1H), 5.27-5.41 (m, 2H), 7.46 (dd, *J* = 8.2, 4.2 Hz, 1H), 7.49-7.58 (m, 2H), 8.16 (dd, *J* = 8.3, 1.6 Hz, 1H), 8.73 (dd, *J* = 6.4, 2.6 Hz, 1H), 8.81 (dd, *J* = 4.2, 1.6 Hz, 1H), 9.87 (brs, 1H); ^{13}C NMR (125 MHz, $CDCl_3$) δ 14.2, 21.8, 22.8, 26.8, 27.2, 27.4, 29.2, 29.4, 29.5, 29.6, 29.7, 29.9, 32.0, 32.3, 33.4, 41.4, 116.6, 118.4, 121.8, 121.9, 127.4, 128.1, 129.6, 130.3, 134.3, 136.5, 138.4, 148.4, 169.5; IR (neat) *v* 3348, 3004, 2926, 2854, 2245, 1688, 1527, 1486, 1425, 1388, 1326, 1164, 827, 792, 757, 689 cm^{-1}; Ms (ESI): *m*/*z* = 448.2 $[M+H]^+$.

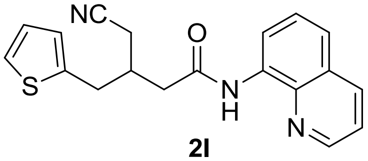

2l

Compound **2l**, colorless oil, yield: 41%. ^{1}H NMR (500 MHz, $CDCl_3$) δ 2.53-2.61 (m, 1H), 2.61-2.68 (m, 1H), 2.68-2.84 (m, 3H), 3.07 (dd, *J* = 14.8, 7.5 Hz, 1H), 3.16 (dd, *J* = 14.8, 5.7 Hz, 1H), 6.90-6.94 (m, 1H), 6.96 (dd, *J* = 5.1, 3.4 Hz, 1H), 7.20 (dd, *J* = 5.1, 1.2 Hz, 1H), 7.47 (dd, *J* = 8.3, 4.2 Hz, 1H), 7.50-7.59 (m, 2H), 8.17 (dd, *J* = 8.3, 1.7 Hz, 1H), 8.69-8.77 (m, 1H), 8.81 (dd, *J* = 4.2, 1.7 Hz, 1H), 9.87 (brs, 1H); ^{13}C NMR (125 MHz, $CDCl_3$) δ 21.2, 33.5, 34.5, 40.4, 116.7, 118.1, 121.9, 122.0, 124.7, 126.6, 127.3, 127.4, 128.1, 134.2, 136.5, 138.4, 140.3, 148.4, 168.9; IR (neat) *v* 3343, 3066, 2924, 2851, 2245, 1658, 1527, 1486, 1388, 1326, 1261, 1164, 827, 792, 757, 700 cm^{-1}; Ms (ESI): *m*/*z* = 336.0 $[M+H]^+$.

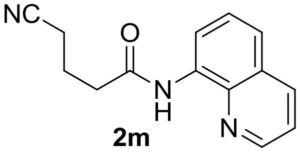

2m

Compound **2m**, colorless oil, yield: 57%. ^{1}H NMR (500 MHz, $CDCl_3$) δ 2.10-2.22 (m, 2H), 2.57 (t, *J* = 7.0 Hz, 2H), 2.76 (t, *J* = 7.0 Hz, 2H), 7.46 (dd, *J* = 8.2, 4.1 Hz,

1H), 7.49-7.59 (m, 2H), 8.16 (d, J = 8.3 Hz, 1H), 8.67-8.77 (m, 1H), 8.77-8.84 (m, 1H), 9.85 (brs, 1H); ^{13}C NMR (125 MHz, $CDCl_3$) δ 16.8, 21.2, 35.7, 116.6, 119.4, 121.8, 121.9, 127.4, 128.1, 134.3, 136.5, 138.4, 148.4, 169.6; IR (neat) ν 3347, 3049, 2941, 2246, 1685, 1527, 1486, 1425, 1387, 1325, 1160, 827, 792, 759, 681 cm^{-1}; Ms (ESI): m/z = 240.1 $[M+H]^+$.

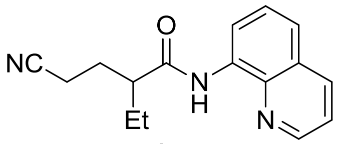

4a

Compound **4a**, colorless oil, yield: 51%. ^{1}H NMR (500 MHz, $CDCl_3$) δ 1.04 (t, J = 7.4 Hz, 3H), 1.64-1.77 (m, 1H), 1.81-1.95 (m, 2H), 2.12-2.25 (m, 1H), 2.37-2.56 (m, 2H), 2.58-2.69 (m, 1H), 7.46 (dd, J = 8.3, 4.2 Hz, 1H), 7.50-7.59 (m, 2H), 8.16 (dd, J = 8.3, 1.7 Hz, 1H), 8.70-8.80 (m, 1H), 8.82 (dd, J = 4.2, 1.6 Hz, 1H), 9.96 (brs, 1H); ^{13}C NMR (125 MHz, $CDCl_3$) δ 11.8, 15.5, 26.2, 27.9, 48.9, 116.7, 119.5, 121.8, 122.0, 127.4, 128.0, 134.2, 136.5, 138.5, 148.5, 172.7; IR (neat) ν 3347, 3049, 2965, 2934, 2876, 2245, 1684, 1525, 1486, 1425, 1379, 1324, 1162, 827, 759, 686, 605 cm^{-1}; Ms (ESI): m/z = 268.2 $[M+H]^+$.

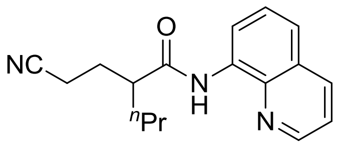

4b

Compound **4b**, colorless oil, yield: 55%. ^{1}H NMR (500 MHz, $CDCl_3$) δ 0.96 (t, J = 7.3 Hz, 3H), 1.40-1.52 (m, 2H), 1.53-1.65 (m, 1H), 1.77-1.86 (m, 1H), 1.87-1.97 (m, 1H), 2.13-2.25 (m, 1H), 2.38-2.56 (m, 2H), 2.65-2.75 (m, 1H), 7.47 (dd, J = 8.3, 4.2 Hz, 1H), 7.49-7.58 (m, 2H), 8.16 (dd, J = 8.3, 1.6 Hz, 1H), 8.70-8.80 (m, 1H), 8.83 (dd, J = 4.2, 1.6 Hz, 1H), 9.96 (brs, 1H); ^{13}C NMR (125 MHz, $CDCl_3$) δ 14.2, 15.5, 20.7, 28.2, 35.3, 47.4, 116.7, 119.5, 121.9, 122.0, 127.4, 128.0, 134.2, 136.5, 138.5, 148.5, 172.8; IR (neat) ν 3347, 2958, 2932, 2872, 2241, 1684, 1526, 1486, 1425, 1324, 1162, 827, 792, 758, 685, 607 cm^{-1}; Ms (ESI): m/z = 282.1 $[M+H]^+$.

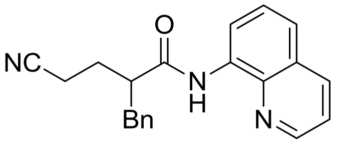

4c

Compound **4c**, pale yellow oil, yield: 50%. ^{1}H NMR (500 MHz, $CDCl_3$) δ 1.87-1.98 (m, 1H), 2.17-2.28 (m, 1H), 2.38-2.47 (m, 1H), 2.47-2.56 (m, 1H), 2.85-3.03 (m, 2H), 3.16 (dd, J = 13.1, 7.4 Hz, 1H), 7.10-7.18 (m, 1H), 7.19-7.30 (m, 4H), 7.43 (dd, J = 8.3, 4.2 Hz, 1H), 7.48-7.58 (m, 2H), 8.14 (dd, J = 8.3, 1.6 Hz, 1H), 8.65-8.80 (m, 2H), 9.78 (brs, 1H); ^{13}C NMR (125 MHz, $CDCl_3$) δ 15.5, 27.7, 39.4, 49.4, 116.8, 119.3, 121.8, 122.1, 126.9, 127.4, 128.0, 128.8, 129.1, 134.0, 136.4, 138.3, 138.4, 148.3, 171.9; IR (neat) ν 3344, 3061, 2928, 2856, 2246, 1684, 1527, 1486, 1425, 1324, 1161, 827, 792, 730, 701, 607 cm^{-1}; Ms (ESI): m/z = 330.1 $[M+H]^{+}$.

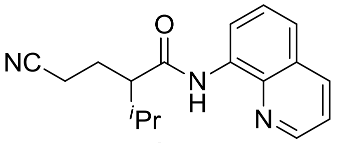

4d

Compound **4d**, colorless oil, yield: 66%. ^{1}H NMR (500 MHz, $CDCl_3$) δ 1.06 (d, J = 7.3 Hz, 3H), 1.08 (d, J = 7.3 Hz, 3H), 1.90-2.01 (m, 1H), 2.01-2.12 (m, 1H), 2.14-2.26 (m, 1H), 2.33-2.57 (m, 3H), 7.46 (dd, J = 8.3, 4.2 Hz, 1H), 7.49-7.59 (m, 2H), 8.16 (dd, J = 8.2, 1.7 Hz, 1H), 8.71-8.80 (m, 1H), 8.82 (dd, J = 4.2, 1.6 Hz, 1H), 9.94 (brs, 1H); ^{13}C NMR (125 MHz, $CDCl_3$) δ 15.8, 20.2, 20.8, 25.6, 31.3, 54.3, 116.7, 119.6, 121.9, 122.0, 127.4, 128.1, 134.1, 136.5, 138.5, 148.5, 172.2; IR (neat) ν 3348, 2963, 2874, 2245, 1683, 1526, 1486, 1425, 1388, 1323, 1161, 827, 792, 758, 697, 606 cm^{-1}; Ms (ESI): m/z = 282.1 $[M+H]^{+}$.

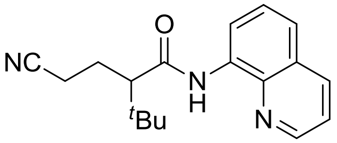

4e

Compound **4e**, colorless oil, yield: 60%. ^{1}H NMR (500 MHz, $CDCl_3$) δ 1.11 (s, 9H), 1.86-2.00 (m, 1H), 2.22-2.36 (m, 2H), 2.36-2.45 (m, 1H), 2.45-2.56 (m, 1H), 7.47 (dd, J = 8.2, 4.2 Hz, 1H), 7.49-7.59 (m, 2H), 8.17 (dd, J = 8.2, 1.6 Hz, 1H), 8.72-8.81 (m, 1H), 8.83 (dd, J = 4.2, 1.7 Hz, 1H), 9.94 (brs, 1H); ^{13}C NMR (125

MHz, $CDCl_3$) δ 16.3, 24.1, 28.2, 33.7, 58.0, 116.7, 119.6, 121.9, 122.0, 127.4, 128.1, 134.1, 136.5, 138.6, 148.6, 171.8; IR (neat) *v* 3351, 3049, 2926, 2872, 2245, 1683, 1526, 1486, 1425, 1370, 1324, 1157, 827, 758, 652, 605 cm^{-1}; Ms (ESI): *m*/*z* = 296.1 $[M+H]^+$.

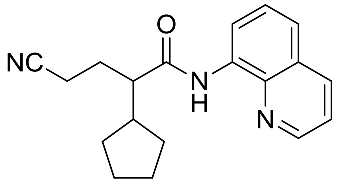

4f

Compound **4f**, colorless oil, yield: 60%. ^1H NMR (500 MHz, $CDCl_3$) δ 1.24-1.42 (m, 2H), 1.47-1.75 (m, 4H), 1.77-1.87 (m, 1H), 1.88-2.06 (m, 2H), 2.09-2.26 (m, 2H), 2.35-2.57 (m, 3H), 7.47 (dd, *J* = 8.3, 4.2 Hz, 1H), 7.50-7.60 (m, 2H), 8.17 (dd, *J* = 8.3, 1.7 Hz, 1H), 8.71-8.80 (m, 1H), 8.83 (dd, *J* = 4.2, 1.7 Hz, 1H), 9.96 (brs, 1H); ^{13}C NMR (125 MHz, $CDCl_3$) δ 15.7, 24.95, 25.04, 27.5, 30.9, 31.4, 43.3, 53.4, 116.8, 119.6, 121.9, 122.1, 127.4, 128.1, 134.2, 136.5, 138.5, 148.6, 172.7; IR (neat) *v* 3348, 2953, 2869, 2245, 1684, 1525, 1486, 1425, 1387, 1324, 1162, 827, 793, 758, 688 cm^{-1}; Ms (ESI): *m*/*z* = 208.1 $[M+H]^+$.

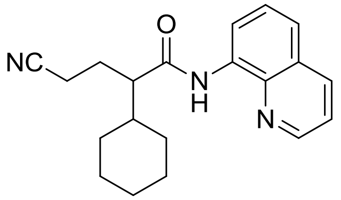

4g

Compound **4g**, colorless oil, yield: 63%. ^1H NMR (500 MHz, $CDCl_3$) δ 1.05-1.33 (m, 5H), 1.60-1.82 (m, 5H), 1.87-1.95 (m, 1H), 1.96-2.06 (m, 1H), 2.13-2.24 (m, 1H), 2.33-2.43 (m, 1H), 2.43-2.55 (m, 2H), 7.47 (dd, *J* = 8.2, 4.2 Hz, 1H), 7.50-7.59 (m, 2H), 8.17 (dd, *J* = 8.3, 1.7 Hz, 1H), 8.73-8.81 (m, 1H), 8.83 (dd, *J* = 4.2, 1.7 Hz, 1H), 9.93 (brs, 1H); ^{13}C NMR (125 MHz, $CDCl_3$) δ 15.7, 25.6, 26.3, 26.4, 30.8, 31.2, 40.7, 53.8, 116.7, 119.6, 121.9, 122.0, 127.4, 128.1, 134.1, 136.5, 138.5, 148.6, 172.4; IR (neat) *v* 3348, 2928, 2852, 2241, 1684, 1526, 1486, 1424, 1380, 1323, 1160, 827, 792, 758 cm^{-1}; Ms (ESI): *m*/*z* = 322.2 $[M+H]^+$.

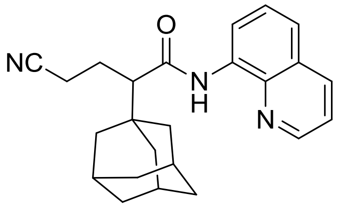

4h

Compound **4h**, colorless oil, yield: 59%. ^{1}H NMR (500 MHz, $CDCl_3$) δ 1.57-1.70 (m, 9H), 1.81-1.90 (m, 3H), 1.92-2.05 (m, 4H), 2.21-2.34 (m, 3H), 2.45-2.55 (m, 1H), 7.47 (dd, *J* = 8.3, 4.2 Hz, 1H), 7.51-7.59 (m, 2H), 8.17 (dd, *J* = 8.2, 1.6 Hz, 1H), 8.75-8.83 (m, 1H), 8.84 (dd, *J* = 4.2, 1.7 Hz, 1H), 9.92 (brs, 1H); ^{13}C NMR (125 MHz, $CDCl_3$) δ 16.3, 22.5, 28.7, 35.6, 36.9, 40.3, 59.4, 116.7, 119.6, 121.9, 122.0, 127.4, 128.1, 134.1, 136.5, 138.6, 148.6, 171.4; IR (neat) ν 3348, 3048, 2904, 2848, 2241, 1683, 1526, 1485, 1425, 1323, 1157, 827, 792, 754 cm^{-1}; Ms (ESI): *m*/*z* = 374.3 $[M+H]^{+}$.

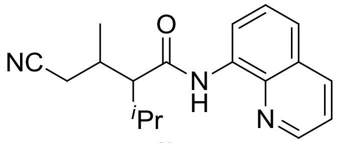

4i

Compound **4i**, colorless oil, yield: 40%. ^{1}H NMR (500 MHz, $CDCl_3$) δ 1.04 (d, *J* = 6.6 Hz, 3H), 1.10 (d, *J* = 6.8 Hz, 3H), 1.26 (d, *J* = 6.8 Hz, 3H), 2.08-2.21 (m, 1H), 2.23-2.31 (m, 1H), 2.39-2.51 (m, 1H), 2.57 (dd, *J* = 17.0, 8.6 Hz, 1H), 2.64 (dd, *J* = 17.0, 4.1 Hz, 1H), 7.46 (dd, *J* = 8.2, 4.2 Hz, 1H), 7.49-7.58 (m, 2H), 8.16 (dd, *J* = 8.3, 1.6 Hz, 1H), 8.71-8.79 (m, 1H), 8.81 (dd, *J* = 4.2, 1.6 Hz, 1H), 9.88 (brs, 1H); ^{13}C NMR (125 MHz, $CDCl_3$) δ 18.2, 19.4, 21.1, 21.5, 28.1, 30.5, 59.5, 116.7, 119.4, 121.8, 122.0, 127.4, 128.1, 134.0, 136.5, 138.5, 148.5, 171.8; IR (neat) ν 3350, 2963, 2933, 2876, 2244, 1680, 1526, 1486, 1425, 1378, 1324, 1167, 827, 793, 758, 679 cm^{-1}; Ms (ESI): *m*/*z* = 296.1 $[M+H]^{+}$.

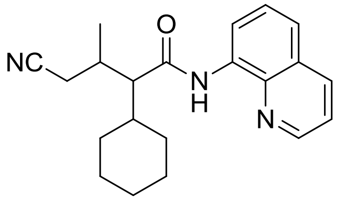

4j

Compound **4j**, colorless oil, yield: 45%. ^{1}H NMR (500 MHz, $CDCl_3$) δ 1.06-1.34 (m, 8H), 1.60-1.90 (m, 6H), 2.25-2.35 (m, 1H), 2.40-2.53 (m, 1H), 2.55-2.68 (m, 2H), 7.47 (dd, J = 8.3, 4.3 Hz, 1H), 7.50-7.60 (m, 2H), 8.17 (dd, J = 8.2, 1.5 Hz, 1H), 8.70-8.79 (m, 1H), 8.81 (dd, J = 4.1, 1.4 Hz, 1H), 9.87 (brs, 1H); ^{13}C NMR (125 MHz, $CDCl_3$) δ 18.3, 21.2, 26.36, 26.42, 26.6, 29.6, 30.1, 31.5, 37.6, 58.9, 116.7, 119.5, 121.8, 122.0, 127.4, 128.1, 134.0, 136.5, 138.5, 148.5, 172.0; IR (neat) v 3350, 3054, 2929, 2852, 2249, 1679, 1525, 1485, 1425, 1325, 1165, 827, 792 cm^{-1}; Ms (ESI): m/z = 336.1 $[M+H]^+$.

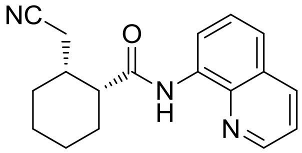

4k

Compound **4k**, colorless oil, yield: 41%. ^{1}H NMR (500 MHz, $CDCl_3$) δ 1.32-1.48 (m, 3H), 1.61-1.71 (m, 1H), 1.77-1.92 (m, 2H), 1.93-2.06 (m, 1H), 2.07-2.27 (m, 2H), 2.32-2.54 (m, 3H), 7.46 (dd, J = 8.3, 4.2 Hz, 1H), 7.50-7.60 (m, 2H), 8.16 (dd, J = 8.3, 1.7 Hz, 1H), 8.70-8.79 (m, 1H), 8.82 (dd, J = 4.2, 1.7 Hz, 1H), 9.94 (brs, 1H); ^{13}C NMR (125 MHz, $CDCl_3$) δ 22.9, 25.35, 25.39, 30.87, 30.90, 35.7, 51.3, 116.7, 118.4, 121.9, 122.0, 127.4, 128.1, 134.3, 136.5, 138.5, 148.5, 172.9; IR (neat) v 3346, 3042, 2931, 2857, 2244, 1683, 1526, 1486, 1425, 1388, 1325, 1163, 931, 827, 792, 668, 600 cm^{-1}; Ms (ESI): m/z = 294.1 $[M+H]^+$.

Experimental Date of Further Optimization Studies

Table S1 Experimental date of further optimization studies for **1a**

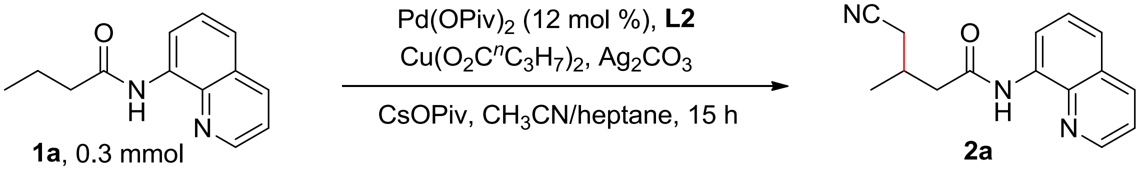

entry	[Cu] (equiv)	[Ag] (equiv)	[Cs] (equiv)	**L2** (mol %)	CH_3CN/heptane (mL/mL)	temp (°C)	yield[a] (%)
1	1.2	2.0	1.2	40	1.5/1.5	130	76
2	2.0	2.0	1.2	40	1.5/1.5	130	62
3	0.5	2.0	1.2	40	1.5/1.5	130	8
4	1.2	3.0	1.2	40	1.5/1.5	130	74
5	1.2	1.0	1.2	40	1.5/1.5	130	50
6	1.2	2.0	2.0	40	1.5/1.5	130	29
7	1.2	2.0	0.5	40	1.5/1.5	130	64
8	1.2	2.0	1.2	50	1.5/1.5	130	76
9	1.2	2.0	1.2	30	1.5/1.5	130	59
10	1.2	2.0	1.2	40	2.0/1.0	130	74
11	1.2	2.0	1.2	40	1.0/2.0	130	73
12	1.2	2.0	1.2	40	1.5/1.5	150	74
13	1.2	2.0	1.2	40	1.5/1.5	110	61
14	1.2	2.0	1.2	40	0.8/0.8	130	61
15	1.2	2.0	1.2	40	3.0/3.0	130	75
16[b]	40	2.0	1.2	40	1.5/1.5	130	67
17[c]	40	2.0	1.2	40	1.5/1.5	130	85

[a] Yields are based on **1a**, determined by ^{1}H-NMR using dibromomethane as the internal standard. [b] $Pd(OPiv)_2$ (10 mol %). [c] $Pd(OPiv)_2$ (15 mol %).

Table S2 Experimental date of further optimization studies for **3b**

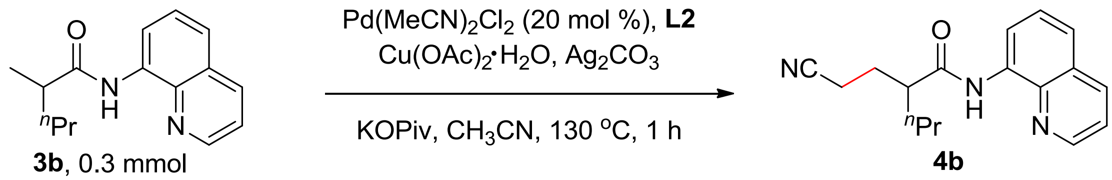

entry	[Cu] (equiv)	[Ag] (equiv)	[K] (equiv)	**L2** (mol %)	CH_3CN (mL)	temp (°C)	yield[a] (%)
1	0.3	2.0	0.3	40	2	130	60
2	0.4	2.0	0.3	40	2	130	56
3	0.2	2.0	0.3	40	2	130	50
4	0	2.0	0.2	40	2	130	trace
5	0.3	3.0	0.3	40	2	130	59
6	0.3	1.5	0.3	40	2	130	54
7	0.3	2.0	0.4	40	2	130	56
8	0.3	2.0	0.1	40	2	130	58
9	0.3	2.0	0	40	2	130	52
10	0.3	2.0	0.3	50	2	130	60
11	0.3	2.0	0.3	30	2	130	50
12	0.3	2.0	0.3	40	2	150	51
13	0.3	2.0	0.3	40	2	110	29
14	0.3	2.0	0.3	40	4	130	42
15	0.3	2.0	0.3	40	1	130	60
16[b]	0.3	2.0	0.3	40	2	130	59

[a] Yields are based on **1a**, determined by ^{1}H-NMR using dibromomethane as the internal standard.
[b] 15 h.

Control Experiments on Cyanomethylation of 1a

Table S3 Control Experiments on Cyanomethylation of **1a**

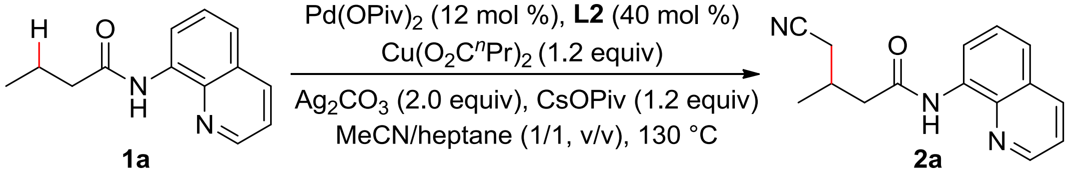

entry	change from the 'standard conditions'[a]	yield of **2a** (%)[b]
1	none	76
2	no $Pd(OPiv)_2$	0
3	no $Cu(O_2C^nPr)_2$	trace
4	0.5 equiv $Cu(O_2C^nPr)_2$	8
5	0.5 equiv $Cu(O_2C^nPr)_2$, O_2 (1 atm)	8
6	no Ag_2CO_3	trace
7	no Ag_2CO_3, 2 equiv $Cu(O_2C^nPr)_2$	trace
8	no Ag_2CO_3, O_2 (1 atm)	trace
9	0.3 equiv Ag_2CO_3, O_2 (1 atm)	12

[a] **1a** (0.3 mmol), $Pd(OPiv)_2$ (0.036 mmol), **L2** (0.12 mmol), $Cu(O_2C^nPr)_2$ (0.36 mmol), Ag_2CO_3 (0.6 mmol), CsOPiv (0.36 mmol), MeCN (1.5 mL), heptane (1.5 mL), air (1 atm), 130 °C, 15 h. [b] Yields are based on **1a**, determined by ^{1}H-NMR using dibromomethane as the internal standard.

Deuterium Labeling Experiment

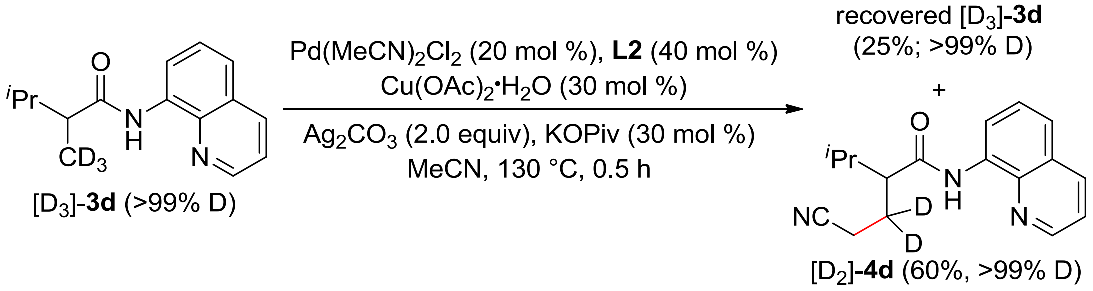

A 35 mL sealed tube was charged with amide [D_3]-**3d** (73.6 mg, 0.3 mmol), $Pd(MeCN)_2Cl_2$ (15.6 mg, 0.06 mmol), 5,5'-dimethyl-2,2'-bipyridine (**L2**) (22.1 mg, 0.12 mmol), $Cu(OAc)_2 \cdot H_2O$ (18.0 mg, 0.09 mmol), Ag_2CO_3 (165.5 mg, 0.6 mmol), KOPiv (12.6 mg, 0.09 mmol), and MeCN (2.0 mL). After sealed, the reaction

mixture was stirred at 130 ˚C for 0.5 h. Then the mixture was cooled to room temperature, quenched with aqueous NaOH (1 M, 2 mL), and extracted with EtOAc (3×10 mL). The combined organic phase was dried over anhydrous Na_2SO_4 and concentrated under reduced pressure. The residue was purified by chromatography on silica gel eluting with EtOAc/hexanes (1/10-1/6) to provide the recovered [D_3]-**3d** and desired product [D_2]-**4d**. The ratio of deuterium was determined by ^{1}H NMR.

[D_2]-**4d**, colorless oil, yield: 60%. ^{1}H NMR (500 MHz, $CDCl_3$) δ 1.06 (d, J = 6.5 Hz, 3H), 1.07 (d, J = 6.5 Hz, 3H), 2.00-2.11 (m, 1H), 2.38 (d, J = 17.0 Hz, 1H), 2.44 (d, J = 7.2 Hz, 1H), 2.49 (d, J = 17.0 Hz, 1H), 7.46 (dd, J = 8.3, 4.2 Hz, 1H), 7.48-7.59 (m, 2H), 8.16 (dd, J = 8.2, 1.4 Hz, 1H), 8.72-8.80 (m, 1H), 8.82 (dd, J = 4.2, 1.6 Hz, 1H), 9.95 (brs, 1H); ^{13}C NMR (125 MHz, $CDCl_3$) δ 15.6 (t, J = 5.6 Hz), 20.2 (d, J = 2.9 Hz), 20.7 (d, J = 2.7 Hz), 31.2, 54.1 (d, J = 4.7 Hz), 116.8, 119.6, 121.8, 122.0, 127.4, 128.0, 134.1, 136.5, 138.4, 148.4, 172.3; IR (neat) ν 3348, 2962, 2928, 2874, 2239, 1683, 1526, 1486, 1425, 1387, 1324, 1168, 827, 792 cm^{-1}; Ms (ESI): m/z = 284.1 [M+H]$^+$.

Recovered [D_3]-**4d**, colorless oil, yield: 25%. ^{1}H NMR (500 MHz, $CDCl_3$) δ 1.03 (d, J = 6.7 Hz, 3H), 1.04 (d, J = 6.7 Hz, 3H), 1.97-2.13 (m, 1H), 2.32 (d, J = 7.7 Hz, 1H), 7.44 (dd, J = 8.3, 4.2 Hz, 1H), 7.49 (dd, J = 8.3, 1.4 Hz, 1H), 7.51-7.60 (m, 1H), 8.15 (dd, J = 8.3, 1.7 Hz, 1H), 8.71-8.88 (m, 2H), 9.84 (brs, 1H).

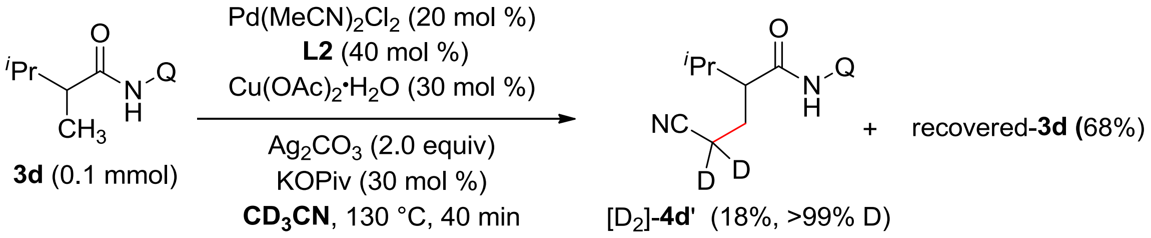

A 10 mL sealed tube was charged with amide **3d** (0.1 mmol), $Pd(MeCN)_2Cl_2$ (5.2 mg, 0.02 mmol), 5,5'-dimethyl-2,2'-bipyridine (**L2**) (7.4 mg, 0.04 mmol), $Cu(OAc)_2\cdot H_2O$ (6.0 mg, 0.03 mmol), Ag_2CO_3 (55.2 mg, 0.2 mmol), KOPiv (4.2 mg, 0.03 mmol), and MeCN or CD_3CN (0.7 mL). After sealed, the reaction mixture was stirred at 130 ˚C for 40 min. Then the mixture was cooled to room temperature, quenched with aqueous NaOH (1 M, 1 mL), and extracted with

EtOAc (3×5 mL). The combined organic phase was dried over anhydrous Na_2SO_4 and concentrated under reduced pressure. The residue was purified by chromatography on silica gel eluting with EtOAc/hexanes (1/8) to provide the recovered **3d** and desired product [D_2]-**4d′**. The ratio of deuterium was determined by ^{1}H NMR.

[D_2]-**4d′**, colorless oil, yield: 18%. ^{1}H NMR (500 MHz, $CDCl_3$) δ 1.07 (d, J = 6.8 Hz, 3H), 1.08 (d, J = 6.8 Hz, 3H), 1.96 (dd, J = 13.6, 2.8 Hz, 1H), 2.00-2.13 (m, 1H), 2.19 (dd, J = 13.4, 11.2 Hz, 1H), 2.39-2.50 (m, 1H), 7.47 (dd, J = 8.3, 4.2 Hz, 1H), 7.50-7.60 (m, 2H), 8.17 (dd, J = 8.2, 1.6 Hz, 1H), 8.71-8.81 (m, 1H), 8.83 (dd, J = 4.2, 1.6 Hz, 1H), 9.94 (brs, 1H); ^{13}C NMR (125 MHz, $CDCl_3$) δ 20.3, 20.8, 25.4, 31.3, 54.3, 116.7, 119.6, 121.9, 122.0, 127.4, 128.1, 134.2, 136.5, 138.6, 148.6, 172.3; IR (neat) ν 3348, 2963, 2246, 1683, 1526, 1486, 1425, 1387, 1324, 1169, 827, 792 cm^{-1}; Ms (ESI): m/z = 284.1 $[M+H]^+$.

Parallel KIE Experiments

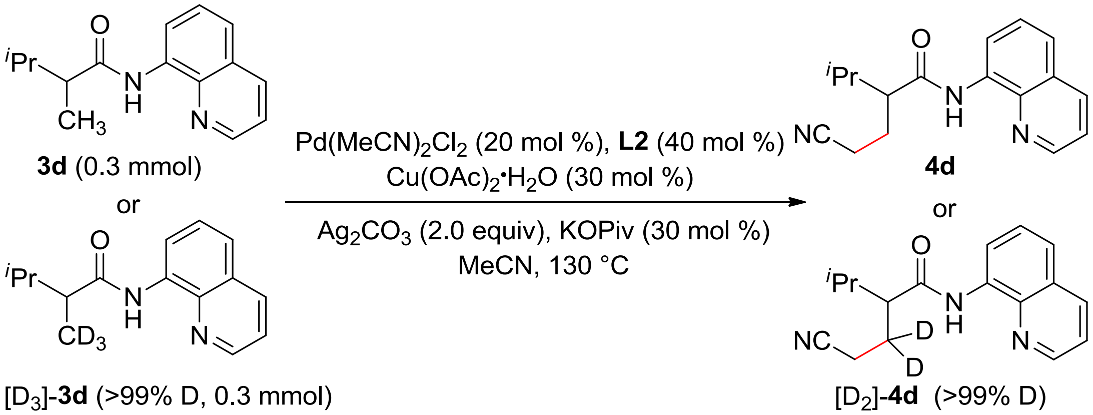

A 35 mL sealed tube was charged with amide **3d** or [D_3]-**3d** (0.3 mmol), $Pd(MeCN)_2Cl_2$ (15.6 mg, 0.06 mmol), 5,5'-dimethyl-2,2'-bipyridine (**L2**) (22.1 mg, 0.12 mmol), $Cu(OAc)_2\cdot H_2O$ (18.0 mg, 0.09 mmol), Ag_2CO_3 (165.5 mg, 0.6 mmol), KOPiv (12.6 mg, 0.09 mmol), and MeCN (2.0 mL). After sealed, the reaction mixture was stirred at 130 ˚C for the indicated time. The reaction was stopped by rapid cooling and analyzed by GC using benzophenone as the internal standard. The average GC yield was calculated after calibrating the response of GC based on three runs of each reaction.

Time (min)	4	5	6	7	8	9	10	11	12
Yield **4d** (%)	5.4	8.2	10.1	12.9	14.8	17.8	21.7	22.8	23.7

Time (min)	4	5	6	7	8	9	10	11	12
Yield of [D_2]-**4d** (%)	5.8	8.5	10.6	13.5	14.9	18.9	23.8	25.2	27.7

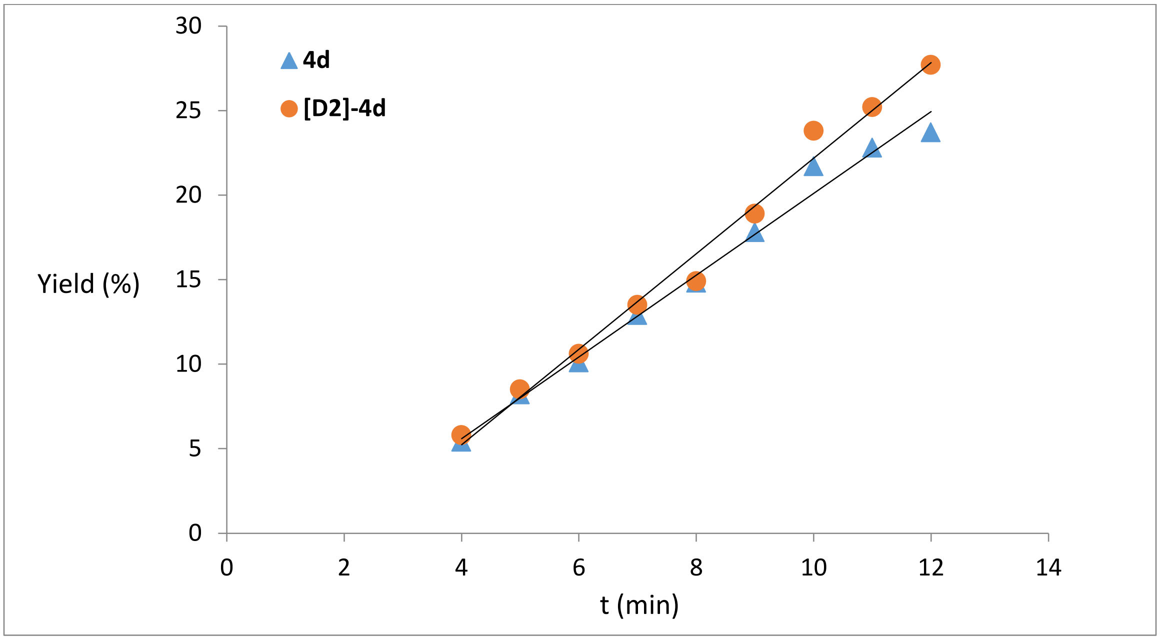

Equation for **4d**: $y = 2.4183x - 4.08$ $R^2 = 0.9871$

Equation for [D_2]-**4d**: $y = 2.825x - 6.0556$ $R^2 = 0.9872$

$k_H/k_D = 2.4183/2.825 \approx 0.9$

KIE value determined from parallel reactions is 0.9.

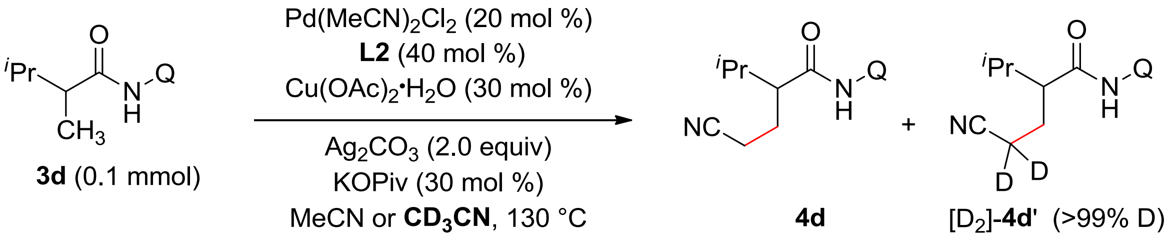

A 10 mL sealed tube was charged with amide **3d** (0.1 mmol), $Pd(MeCN)_2Cl_2$ (5.2 mg, 0.02 mmol), 5,5'-dimethyl-2,2'-bipyridine (**L2**) (7.4 mg, 0.04 mmol), $Cu(OAc)_2 \cdot H_2O$ (6.0 mg, 0.03 mmol), Ag_2CO_3 (55.2 mg, 0.2 mmol), KOPiv (4.2 mg, 0.03 mmol), and MeCN or CD_3CN (0.7 mL). After sealed, the reaction

mixture was stirred at 130 ˚C for the indicated time. The reaction was stopped by rapid cooling and analyzed by GC using benzophenone as the internal standard. The average GC yield was calculated after calibrating the response of GC based on three runs of each reaction.

Time (min)	4	6	8	10	12	14	16	18
Yield **4d** (%)	4.9	5.9	8.6	11.2	15.7	18.2	19.6	22.5

Time (min)	4	6	8	10	12	14	16	18
Yield of [D_2]-**4d′** (%)	3.7	4.2	5.0	6.1	6.7	7.4	9.3	9.8

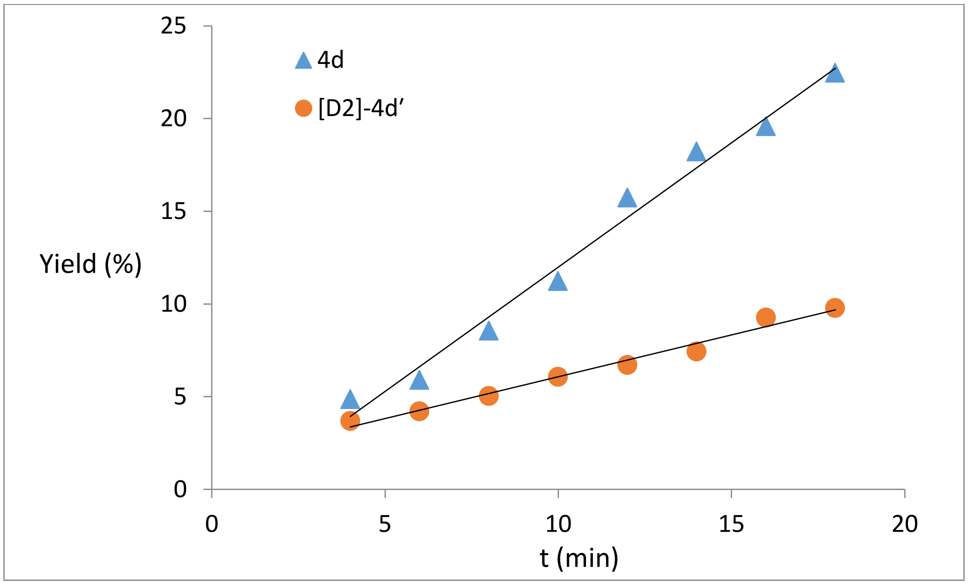

Equation for **4d**: $y = 1.3407 - 1.4259$ $R^2 = 0.9848$

Equation for [D_2]-**4d′**: $y = 0.4508x + 1.5627$ $R^2 = 0.9818$

$k_H/k_D = 1.3407/0.4259 \approx 3.0$

KIE value determined from parallel reactions is 3.0.

Experimental Procedure for the Removal of the Directing Group of 4b

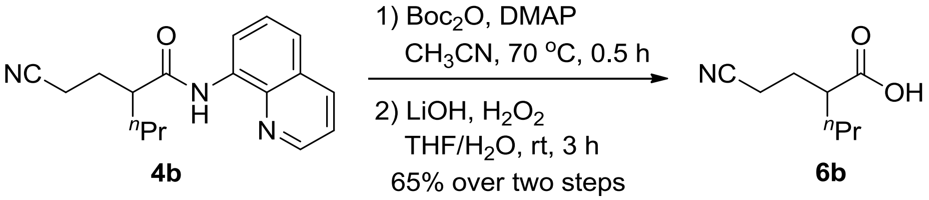

A solution of compound **4b** (84.4 mg, 0.3 mmol), Boc_2O (654 mg, 3 mmol) and DMAP (100 mg, 0.9 mmol) in CH_3CN (2 mL) was stirred at 70 °C for 0.5 h. The resulting mixture was concentrated under reduced pressure and purified by chromatography on silica gel eluting with EtOAc/hexanes (1/3) to provide the boc-protecting amide as yellow oil (108 mg, 94% yield). ^{1}H NMR (500 MHz, $CDCl_3$) δ 0.95 (t, J = 7.3 Hz, 3H), 1.22 (s, 9H), 1.40-1.60 (m, 3H), 1.80-1.91 (m, 1H), 1.94-2.04 (m, 1H), 2.10-2.22 (m, 1H), 2.48-2.69 (m, 2H), 4.00 (brs, 1H), 7.41 (dd, J = 8.3, 4.1 Hz, 1H), 7.49 (dd, J = 7.3, 1.3 Hz, 1H), 7.52-7.61 (m, 1H), 7.82 (dd, J = 8.2, 1.2 Hz, 1H), 8.17 (dd, J = 8.3, 1.6 Hz, 1H), 8.87 (dd, J = 4.1, 1.6 Hz, 1H); ^{13}C NMR (125 MHz, $CDCl_3$) δ 14.3, 15.0, 20.4, 27.7, 28.4, 34.8, 43.8, 83.1, 120.3, 121.7, 126.2, 128.3, 128.7, 129.0, 136.1, 137.2, 144.1, 150.5, 152.8, 178.4; IR (neat) v 2960, 2934, 2245, 1738, 1699, 1596, 1500, 1369, 1292, 1255, 1155, 1125, 1042, 854, 792 cm^{-1}; Ms (ESI): m/z = 382.2 $[M+H]^+$.

To a solution of the boc-protecting amide (76.2 mg, 0.2 mmol) in THF/H_2O (2 mL, 3:1) was added $LiOH{\cdot}H_2O$ (9.6 mg, 0.4 mmol) and 30% H_2O_2 (1.0 mmol) at 0 °C. Ater the reaction was stirred at room temperature for 3 h, Na_2SO_3 (252 mg, 2 mmol) was added. The reaction mixture was diluted with EtOAc (4 mL), acidified with 0.5 M aqueous HCl, and extracted with EtOAc. The organic layer was dried over anhydrous Na_2SO_4 and concentrated under reduced pressure. The resulting residue was purified by chromatography on silica gel eluting with EtOAc/hexanes (1/8-1/1) to provide the acid product 6**b** as pale yellow oil (21.5 mg, 69% yield). ^{1}H NMR (500 MHz, $CDCl_3$) δ 0.93 (t, J = 7.3 Hz, 3H), 1.31-1.44 (m, 2H), 1.46-1.56 (m, 1H), 1.62-1.73 (m, 1H), 1.79-1.89 (m, 1H), 1.95-2.06 (m, 1H), 2.32-2.50 (m, 2H), 2.50-2.61 (m 1H); ^{13}C NMR (125 MHz, $CDCl_3$) δ 13.9, 15.5, 20.3, 27.3, 34.0, 43.9, 119.1, 180.6; IR (neat) v 2960, 2929, 2874, 2248, 1734, 1707, 1528, 1456, 1164; Ms (ESI): m/z = 154.2 $[M-H]^-$.

References

1. X. Wu, Y. Zhao and H. Ge, *J. Am. Chem. Soc.*, 2014, **136**, 1789.
2. E. T. Nadres, G. I. F. Santos, D. Shabashov and O. Daugulis, *J. Org. Chem.*, 2013, **78**, 9689.

Copies of NMR Spectrum

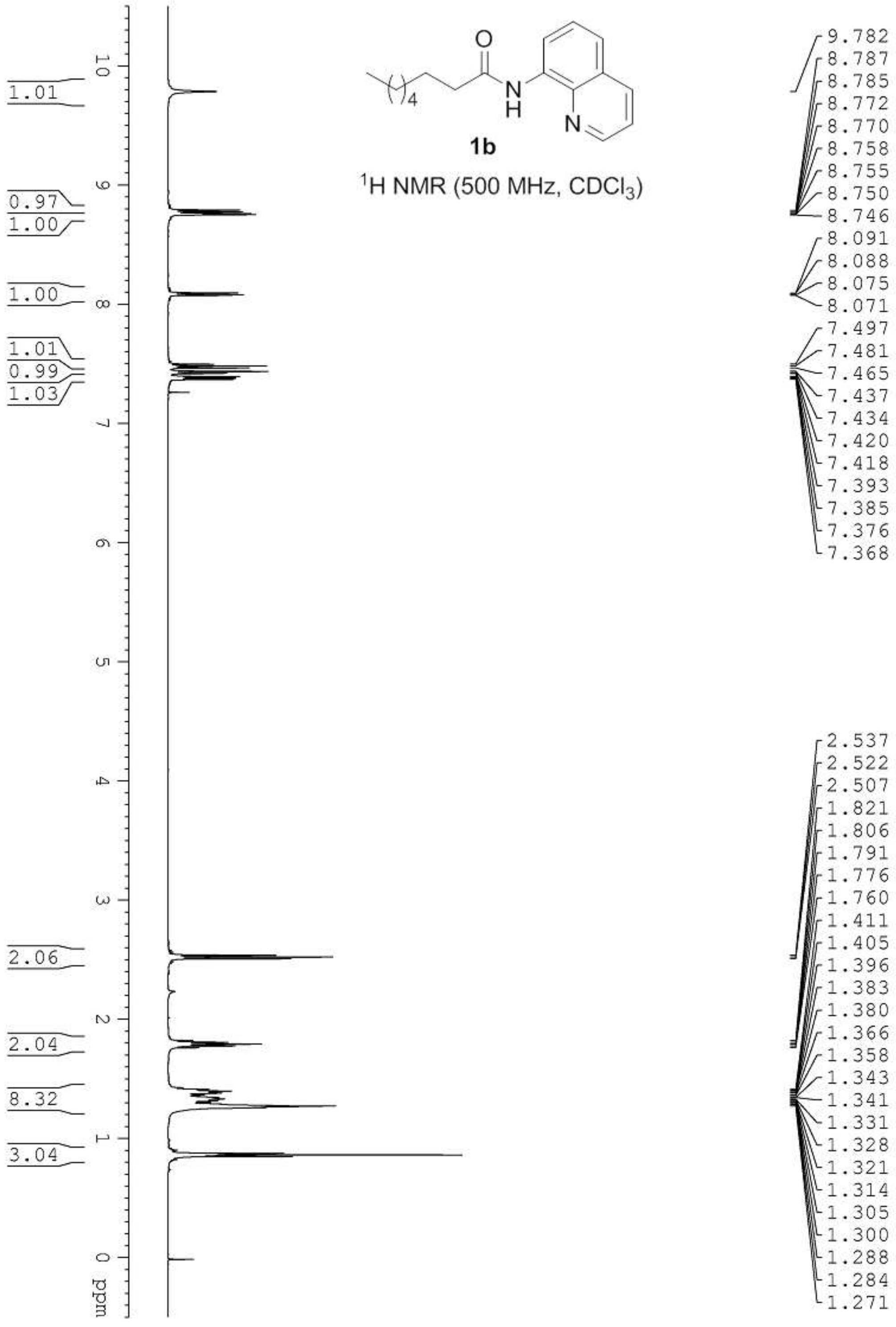

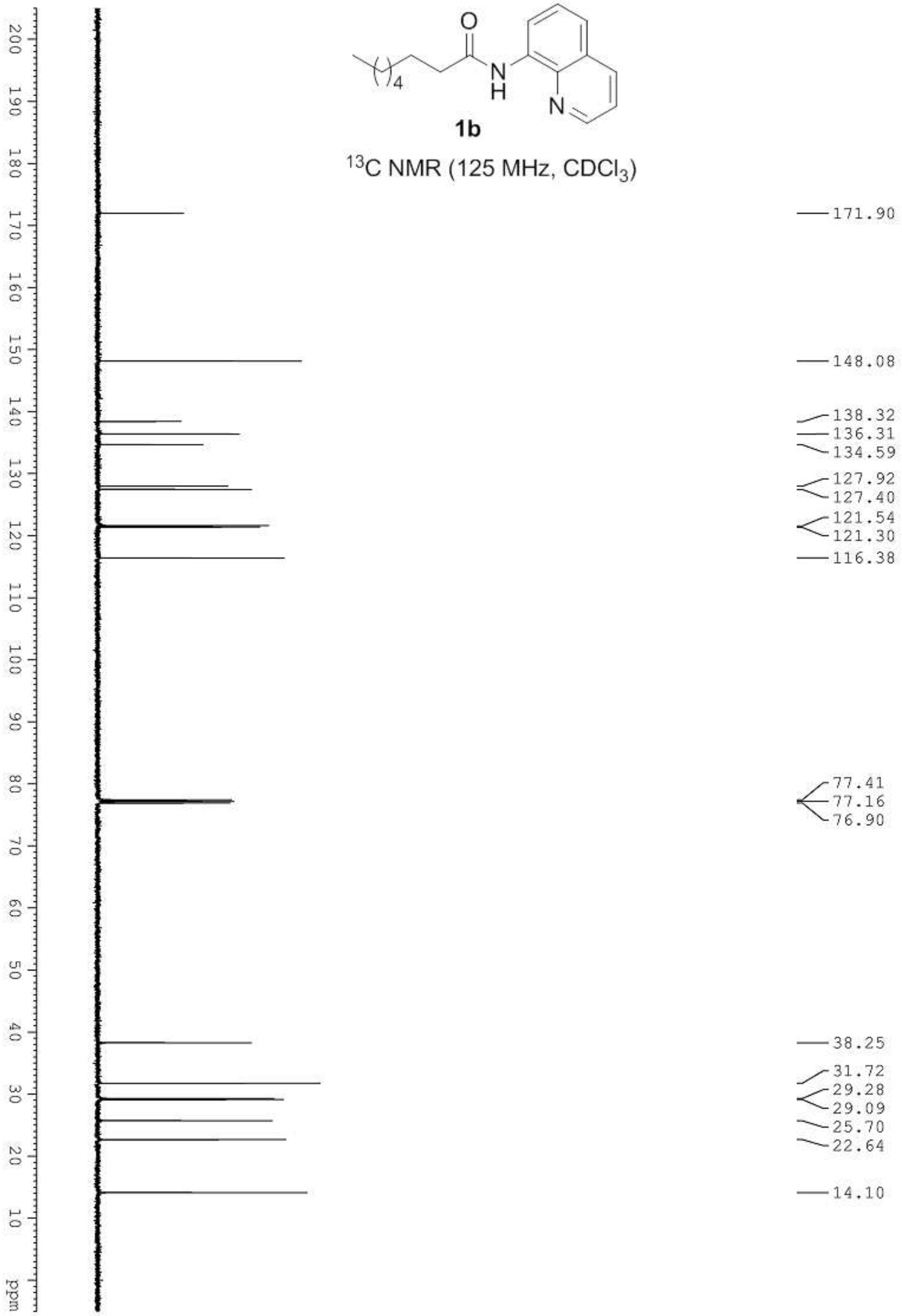
O
N
H
N
4
1b
^{13}C NMR (125 MHz, $CDCl_3$)
171.90
148.08
138.32
136.31
134.59
127.92
127.40
121.54
121.30
116.38
77.41
77.16
76.90
38.25
31.72
29.28
29.09
25.70
22.64
14.10
200
190
180
170
160
150
140
130
120
110
100
90
80
70
60
50
40
30
20
10
ppm

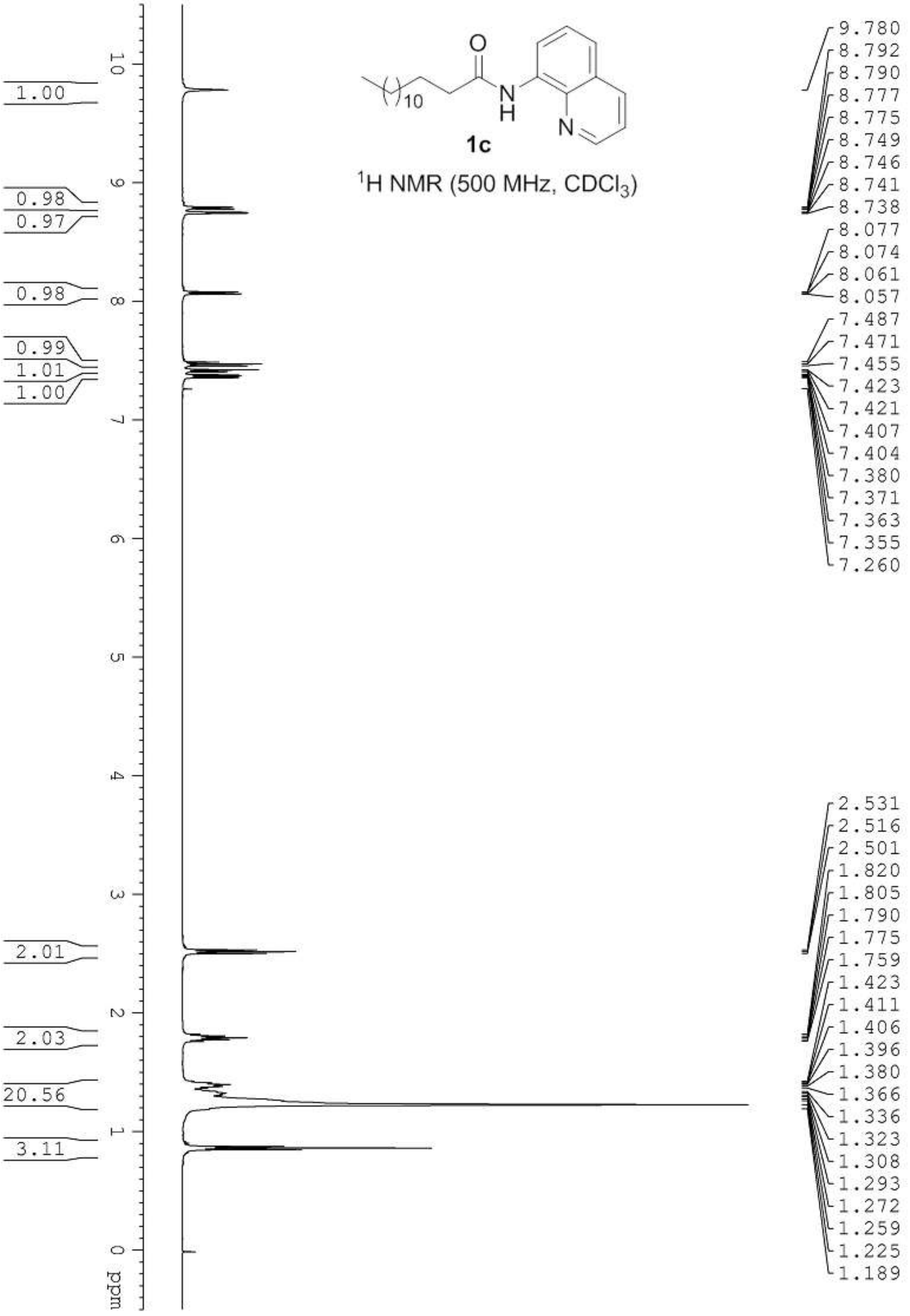
1c
1H NMR (500 MHz, CDCl3)
10
1.00
0.98
0.97
0.98
0.99
1.01
1.00
2.01
2.03
20.56
3.11
9.780
8.792
8.790
8.777
8.775
8.749
8.746
8.741
8.738
8.077
8.074
8.061
8.057
7.487
7.471
7.455
7.423
7.421
7.407
7.404
7.380
7.371
7.363
7.355
7.260
2.531
2.516
2.501
1.820
1.805
1.790
1.775
1.759
1.423
1.411
1.406
1.396
1.380
1.366
1.336
1.323
1.308
1.293
1.272
1.259
1.225
1.189
ppm

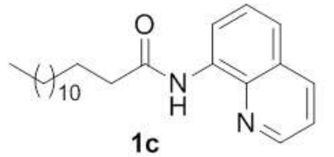

1c

^{13}C NMR (125 MHz, $CDCl_3$)

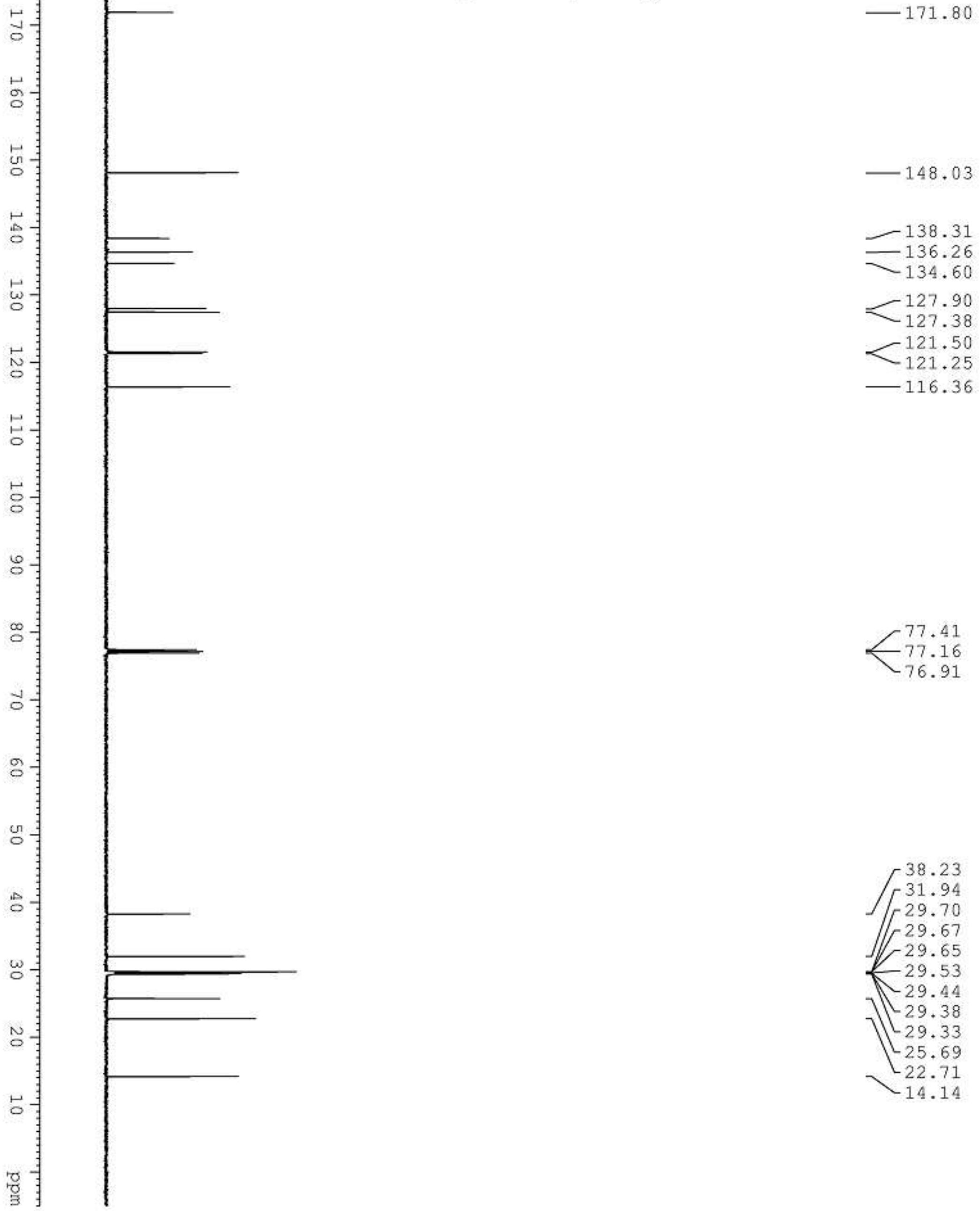

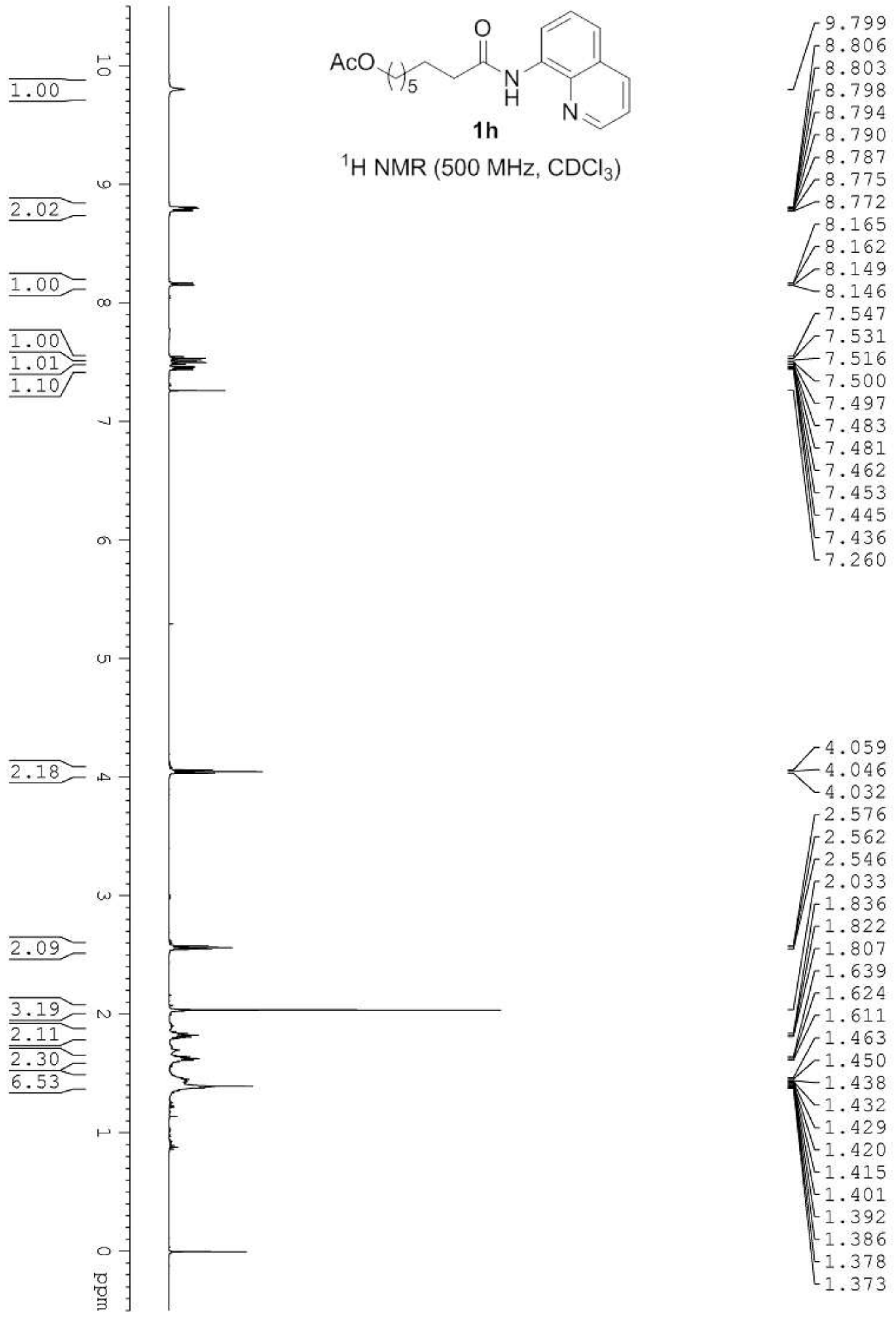
AcO
5
O
N
H
N
1h
¹H NMR (500 MHz, CDCl₃)
1.00
2.02
1.00
1.00
1.01
1.10
2.18
2.09
3.19
2.11
2.30
6.53
9.799
8.806
8.803
8.798
8.794
8.790
8.787
8.775
8.772
8.165
8.162
8.149
8.146
7.547
7.531
7.516
7.500
7.497
7.483
7.481
7.462
7.453
7.445
7.436
7.260
4.059
4.046
4.032
2.576
2.562
2.546
2.033
1.836
1.822
1.807
1.639
1.624
1.611
1.463
1.450
1.438
1.432
1.429
1.420
1.415
1.401
1.392
1.386
1.378
1.373
ppm

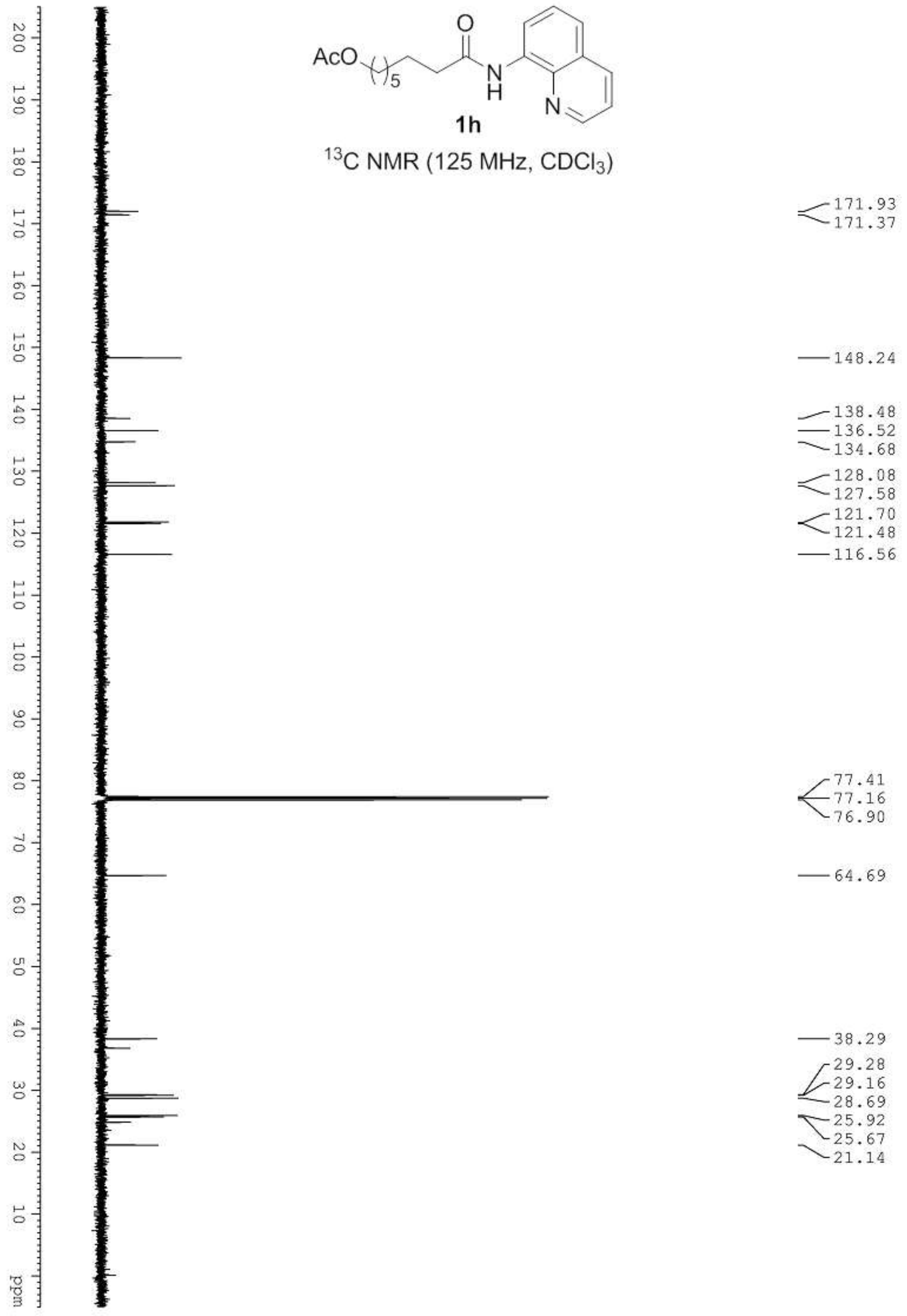
AcO
O
N
H
N
5
1h
^{13}C NMR (125 MHz, $CDCl_3$)
171.93
171.37
148.24
138.48
136.52
134.68
128.08
127.58
121.70
121.48
116.56
77.41
77.16
76.90
64.69
38.29
29.28
29.16
28.69
25.92
25.67
21.14
200
190
180
170
160
150
140
130
120
110
100
90
80
70
60
50
40
30
20
10
ppm

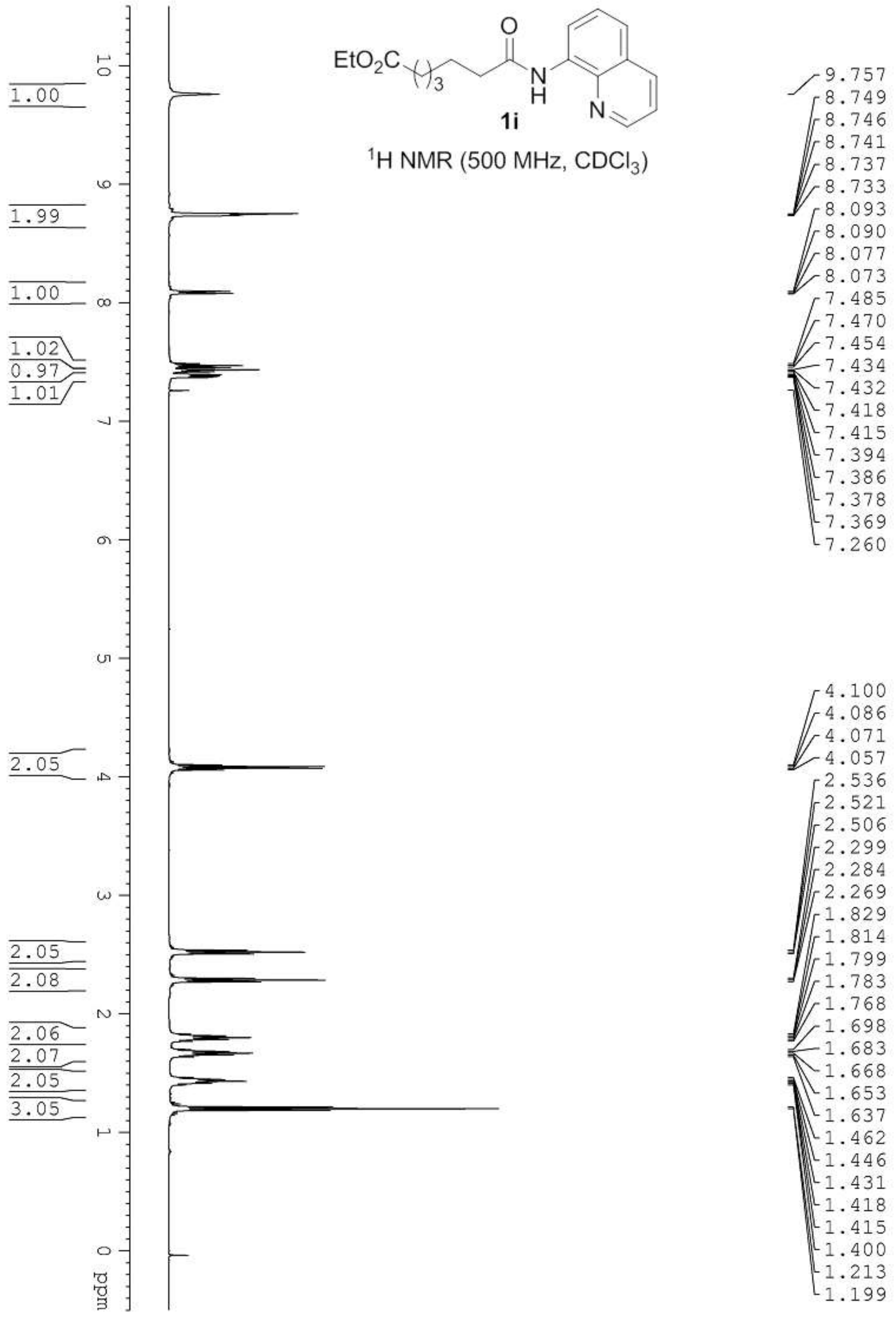

1i

^{1}H NMR (500 MHz, $CDCl_3$)

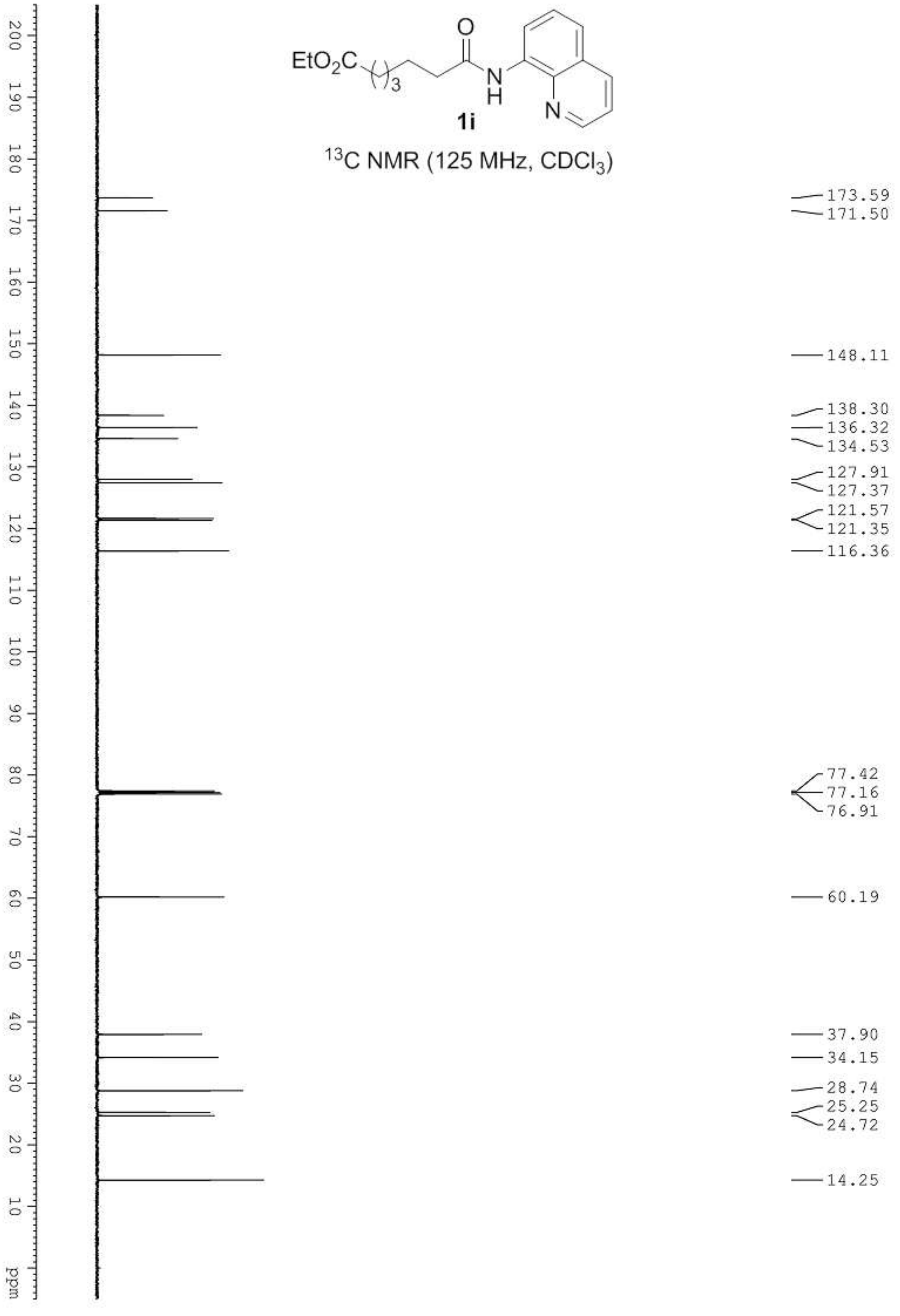
EtO2C
3
O
N
H
N
1i
13C NMR (125 MHz, CDCl3)
173.59
171.50
148.11
138.30
136.32
134.53
127.91
127.37
121.57
121.35
116.36
77.42
77.16
76.91
60.19
37.90
34.15
28.74
25.25
24.72
14.25
ppm

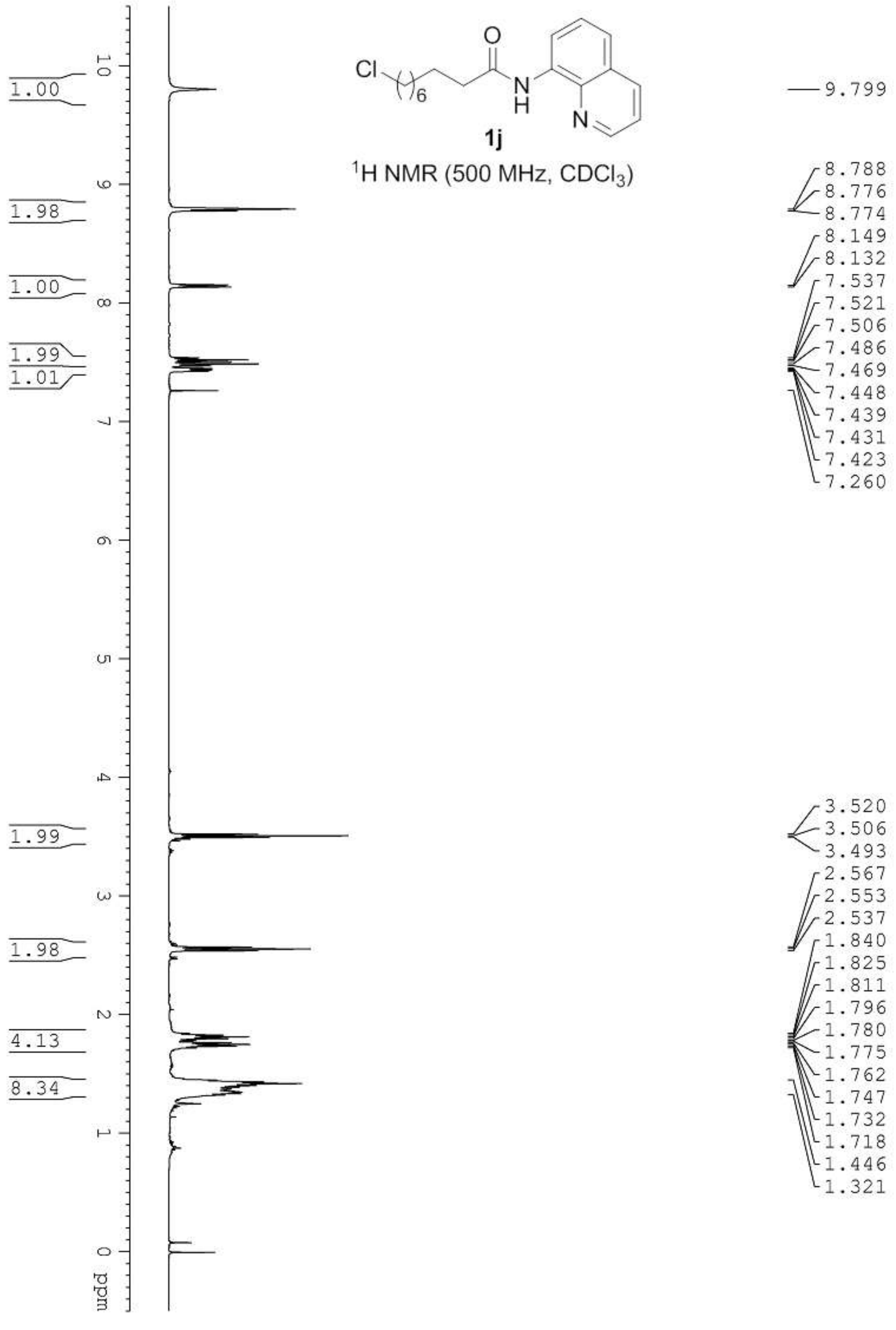
Cl
6
O
N
H
N
1j
^{1}H NMR (500 MHz, CDCl$_3$)
9.799
8.788
8.776
8.774
8.149
8.132
7.537
7.521
7.506
7.486
7.469
7.448
7.439
7.431
7.423
7.260
3.520
3.506
3.493
2.567
2.553
2.537
1.840
1.825
1.811
1.796
1.780
1.775
1.762
1.747
1.732
1.718
1.446
1.321
1.00
1.98
1.00
1.99
1.01
1.99
1.98
4.13
8.34
10
9
8
7
6
5
4
3
2
1
0 ppm

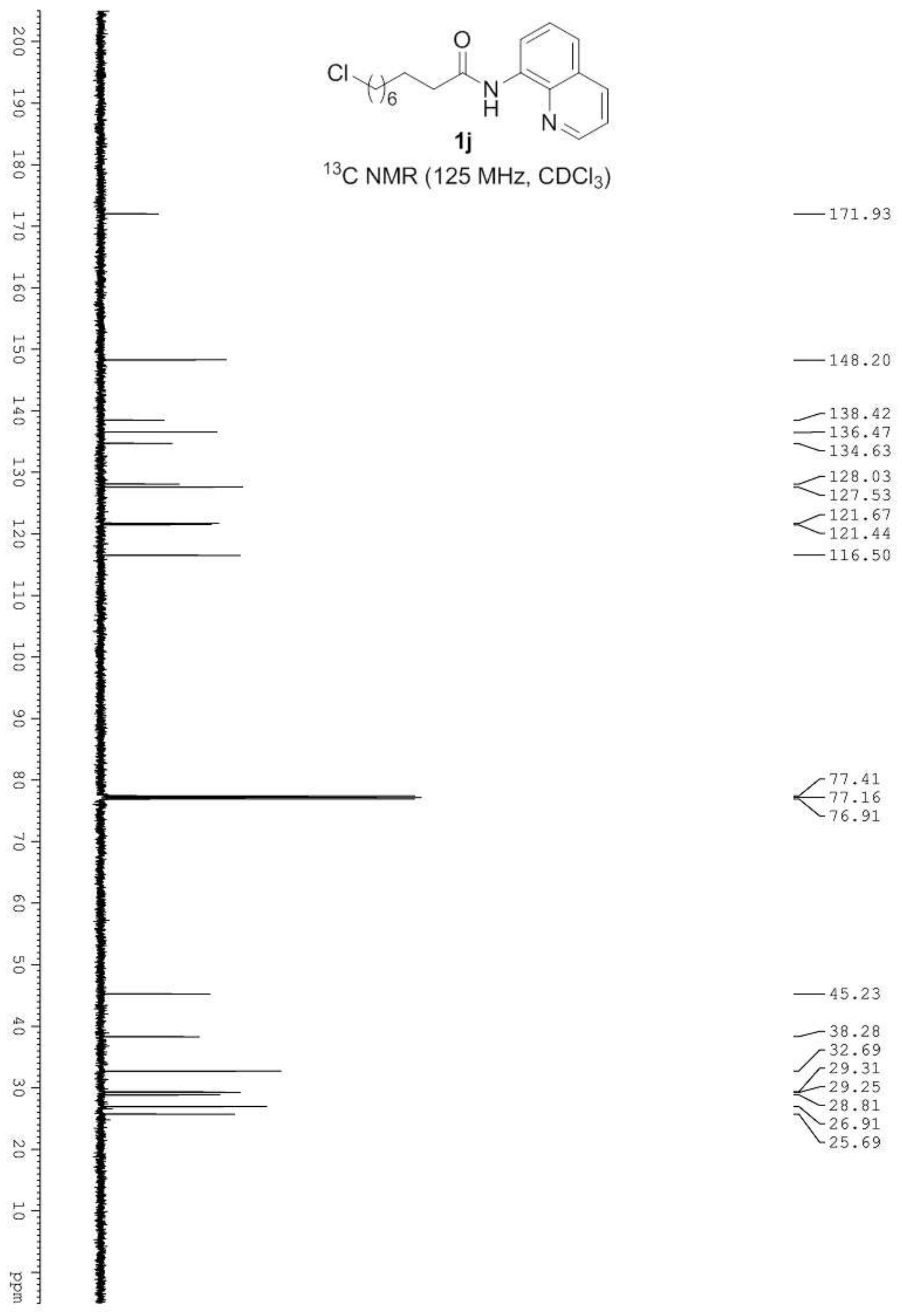

Cl
O
N
H
N
6
1j
13C NMR (125 MHz, CDCl3)
171.93
148.20
138.42
136.47
134.63
128.03
127.53
121.67
121.44
116.50
77.41
77.16
76.91
45.23
38.28
32.69
29.31
29.25
28.81
26.91
25.69
200
190
180
170
160
150
140
130
120
110
100
90
80
70
60
50
40
30
20
10
ppm

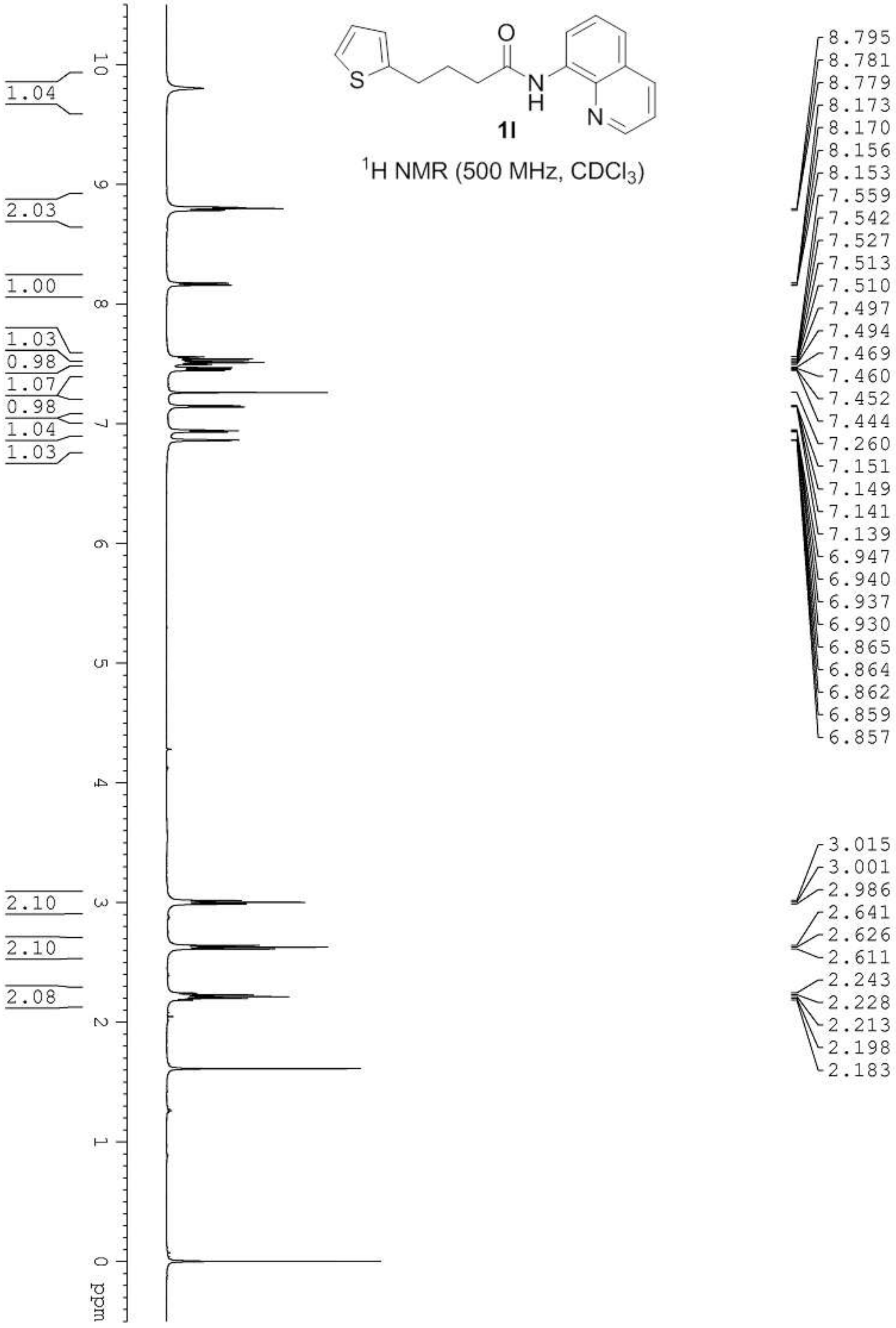
1l
^1H NMR (500 MHz, $CDCl_3$)
8.795
8.781
8.779
8.173
8.170
8.156
8.153
7.559
7.542
7.527
7.513
7.510
7.497
7.494
7.469
7.460
7.452
7.444
7.260
7.151
7.149
7.141
7.139
6.947
6.940
6.937
6.930
6.865
6.864
6.862
6.859
6.857
3.015
3.001
2.986
2.641
2.626
2.611
2.243
2.228
2.213
2.198
2.183
1.04
2.03
1.00
1.03
0.98
1.07
0.98
1.04
1.03
2.10
2.10
2.08
ppm

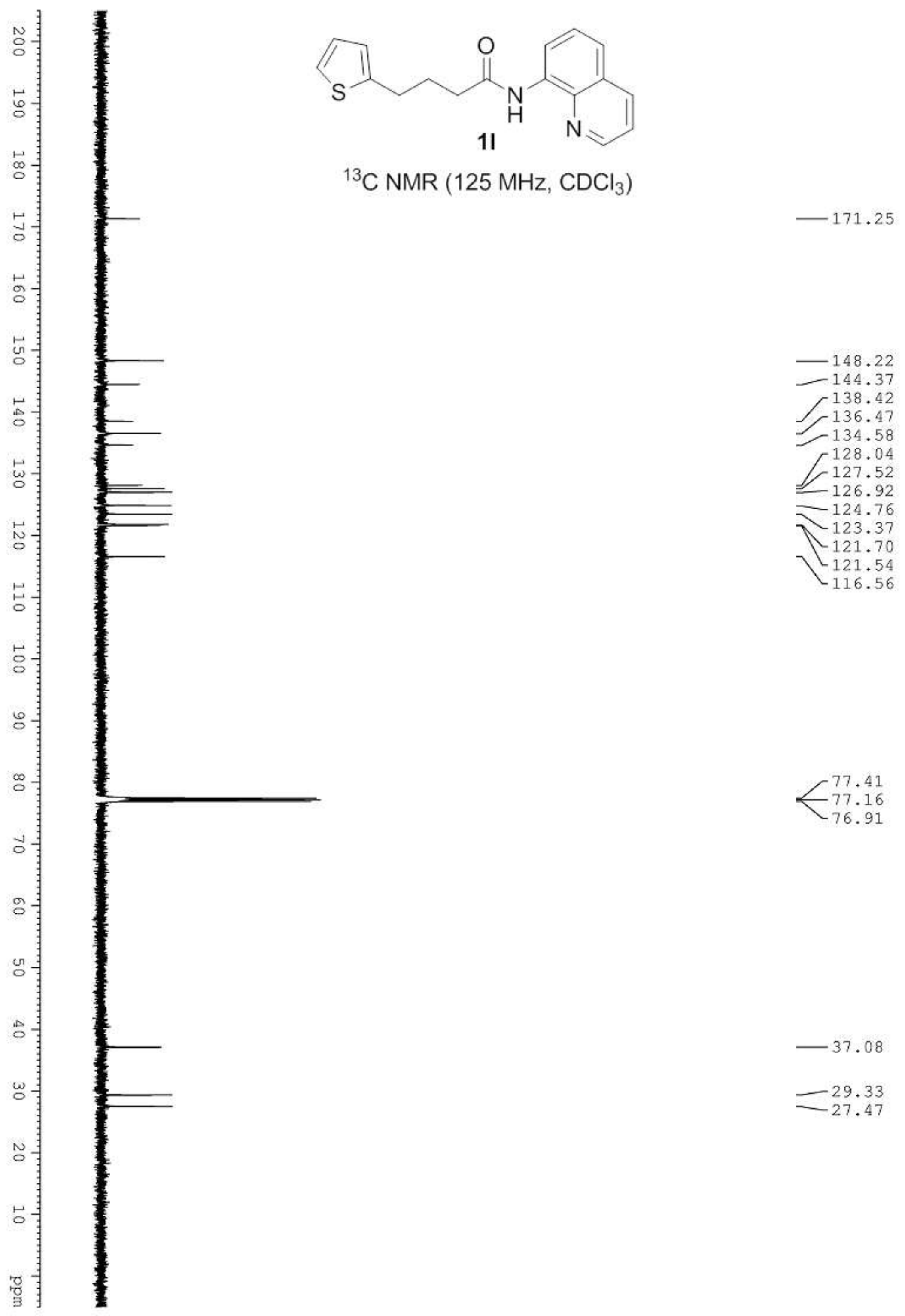
1l
^{13}C NMR (125 MHz, CDCl$_3$)
171.25
148.22
144.37
138.42
136.47
134.58
128.04
127.52
126.92
124.76
123.37
121.70
121.54
116.56
77.41
77.16
76.91
37.08
29.33
27.47
ppm

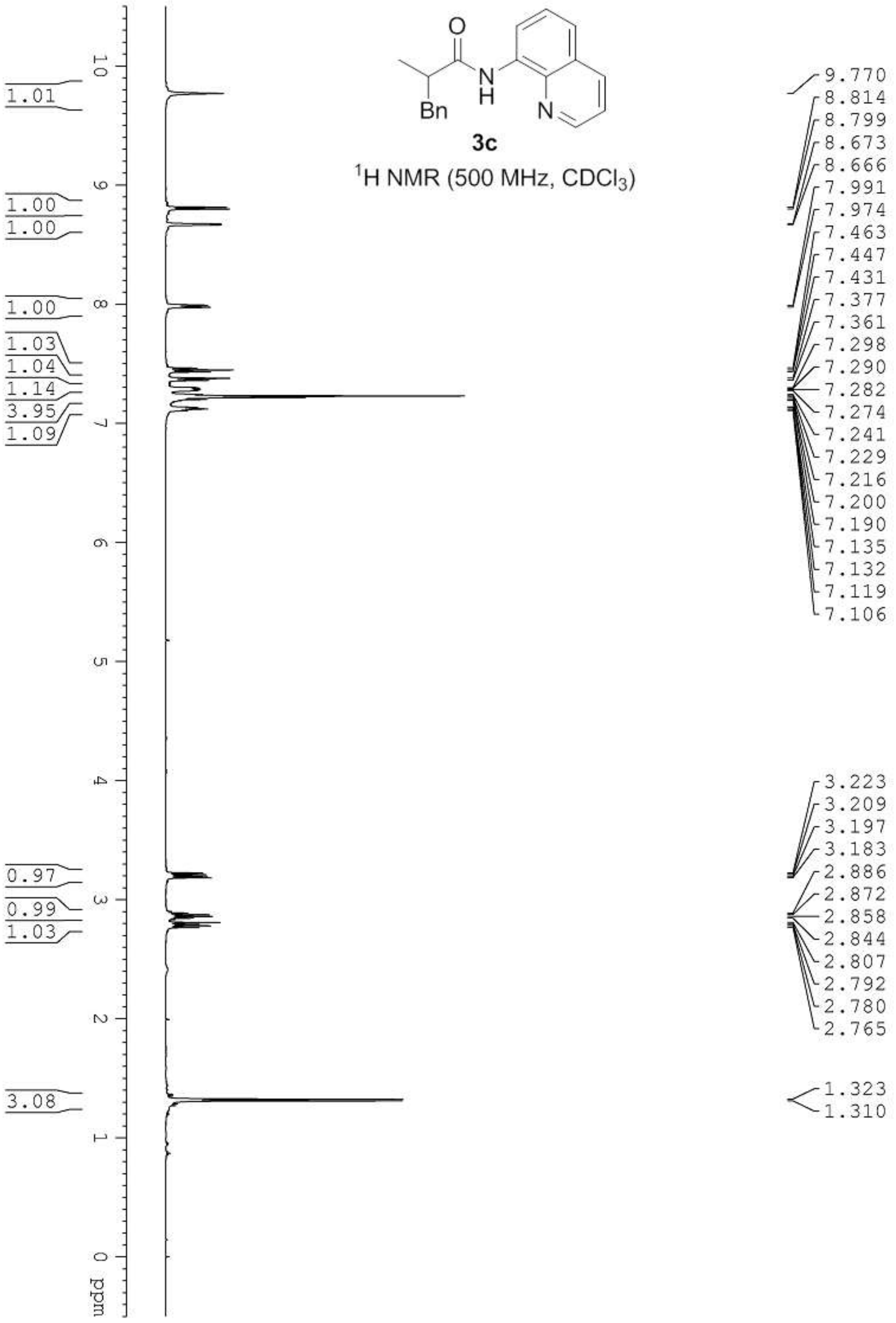
O
N
H
Bn
N
3c
1H NMR (500 MHz, CDCl3)
9.770
8.814
8.799
8.673
8.666
7.991
7.974
7.463
7.447
7.431
7.377
7.361
7.298
7.290
7.282
7.274
7.241
7.229
7.216
7.200
7.190
7.135
7.132
7.119
7.106
3.223
3.209
3.197
3.183
2.886
2.872
2.858
2.844
2.807
2.792
2.780
2.765
1.323
1.310
1.01
1.00
1.00
1.00
1.03
1.04
1.14
3.95
1.09
0.97
0.99
1.03
3.08
ppm

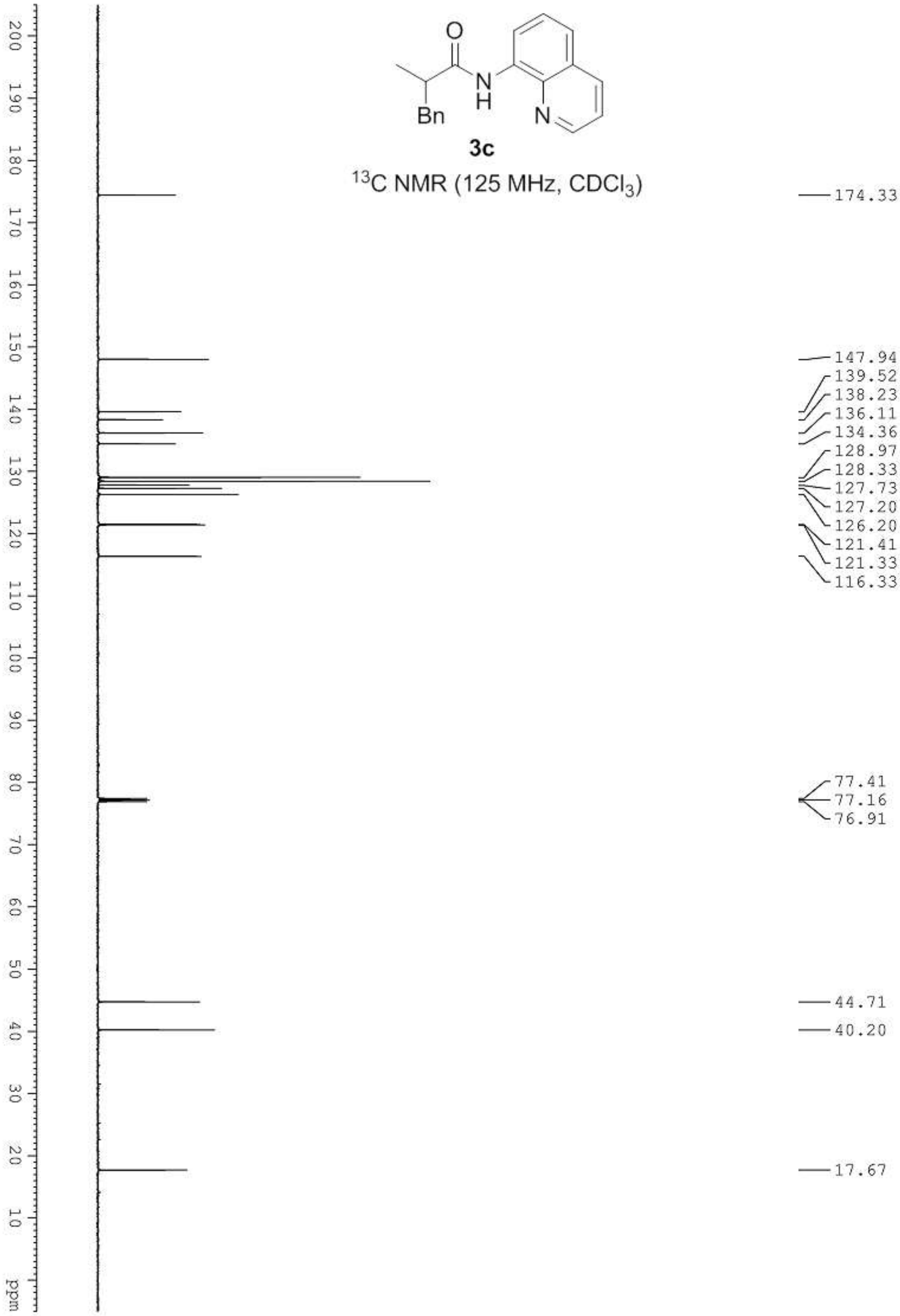

3c

^{13}C NMR (125 MHz, $CDCl_3$)

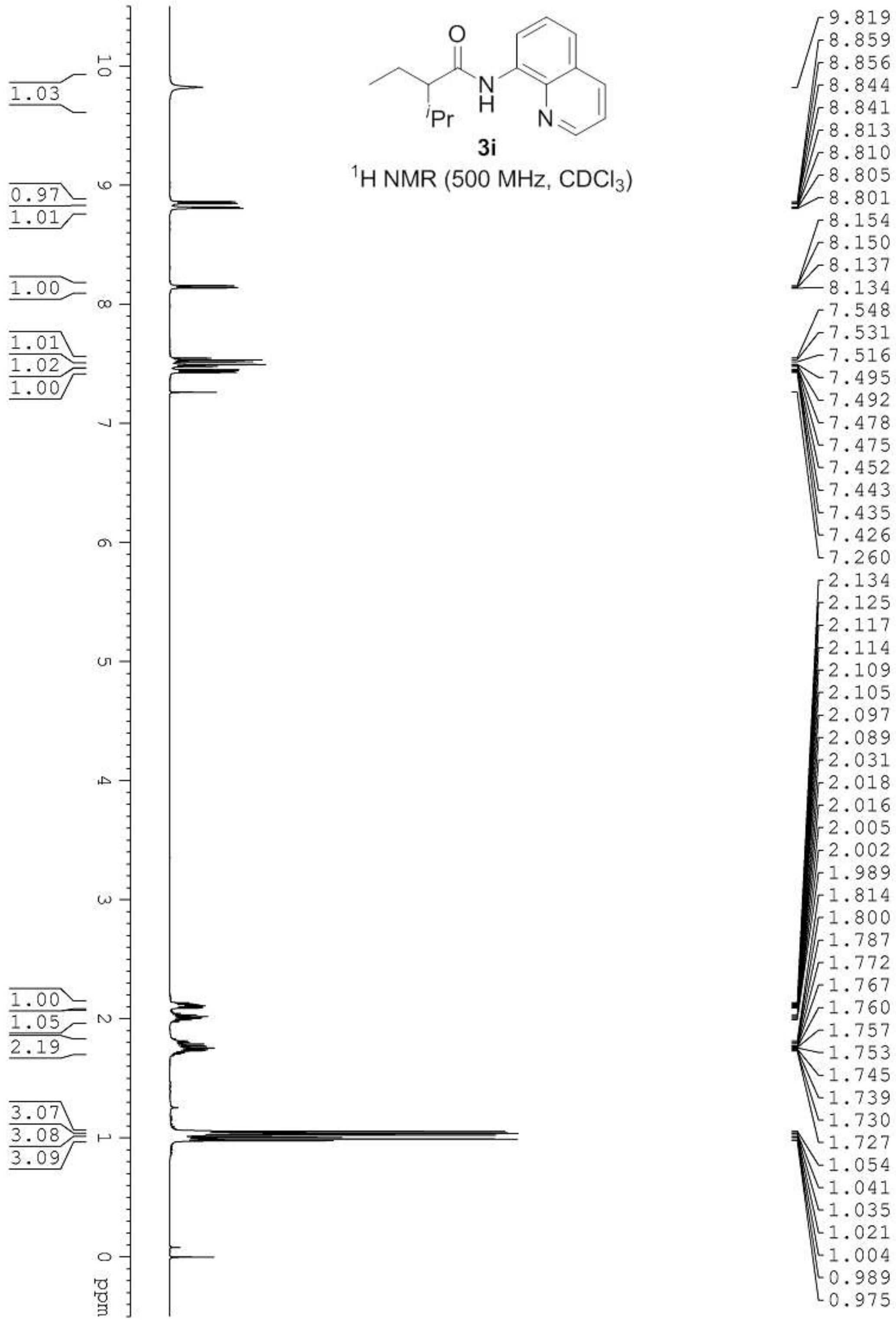
3i
^1H NMR (500 MHz, $CDCl_3$)
9.819
8.859
8.856
8.844
8.841
8.813
8.810
8.805
8.801
8.154
8.150
8.137
8.134
7.548
7.531
7.516
7.495
7.492
7.478
7.475
7.452
7.443
7.435
7.426
7.260
2.134
2.125
2.117
2.114
2.109
2.105
2.097
2.089
2.031
2.018
2.016
2.005
2.002
1.989
1.814
1.800
1.787
1.772
1.767
1.760
1.757
1.753
1.745
1.739
1.730
1.727
1.054
1.041
1.035
1.021
1.004
0.989
0.975
1.03
0.97
1.01
1.00
1.01
1.02
1.00
1.00
1.05
2.19
3.07
3.08
3.09
ppm

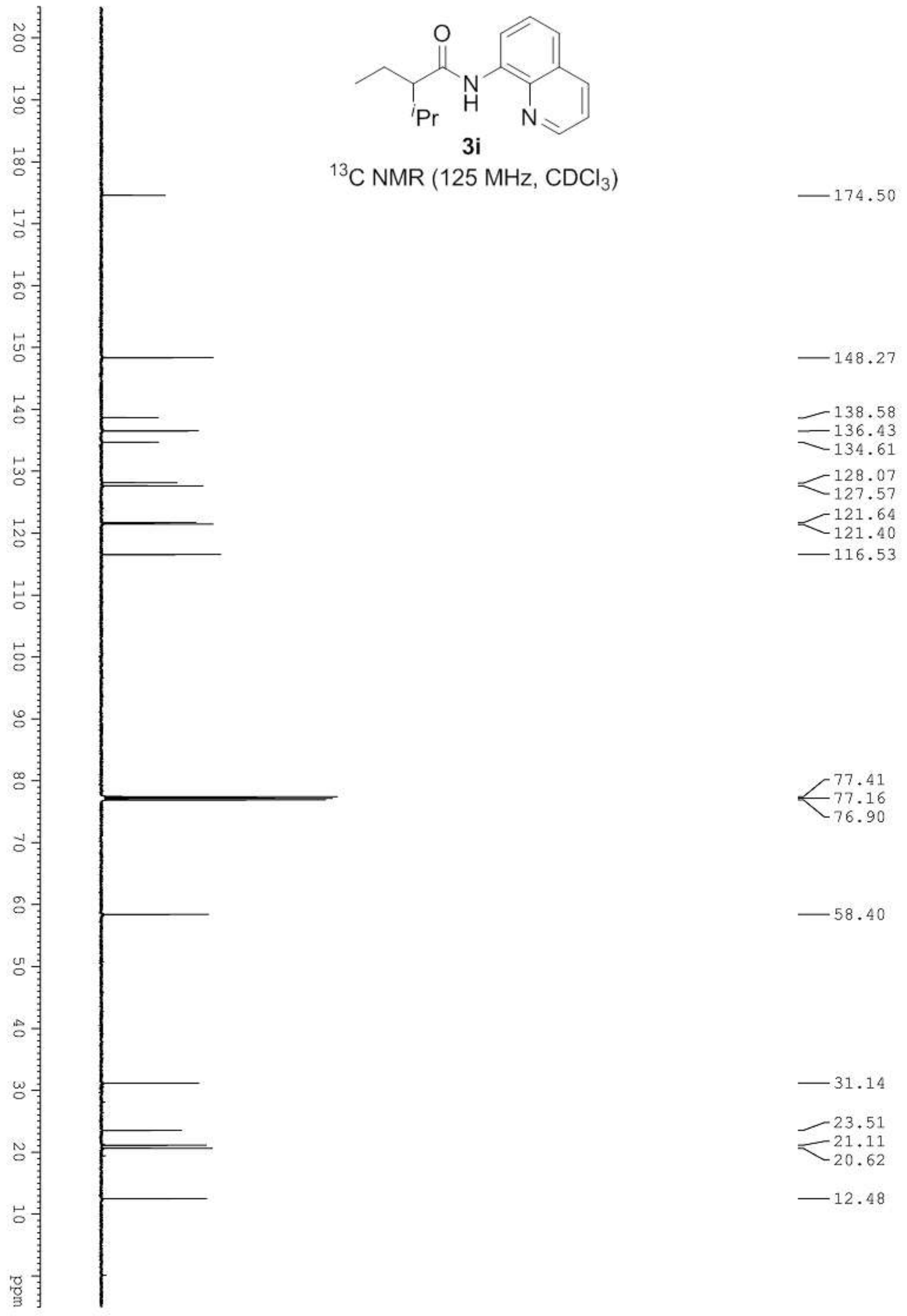
3i
^{13}C NMR (125 MHz, CDCl$_3$)
174.50
148.27
138.58
136.43
134.61
128.07
127.57
121.64
121.40
116.53
77.41
77.16
76.90
58.40
31.14
23.51
21.11
20.62
12.48
ppm

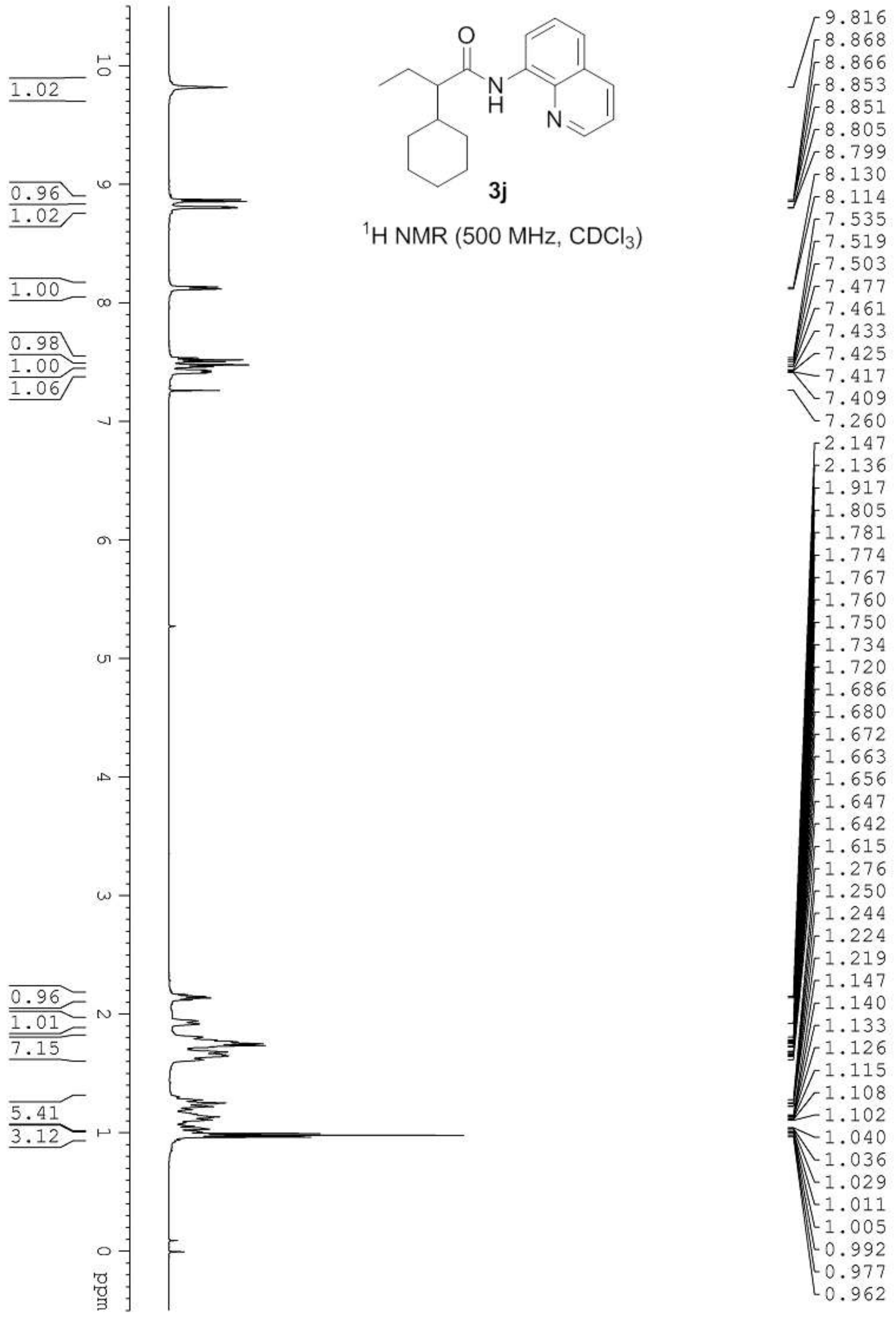

3j

^{1}H NMR (500 MHz, $CDCl_3$)

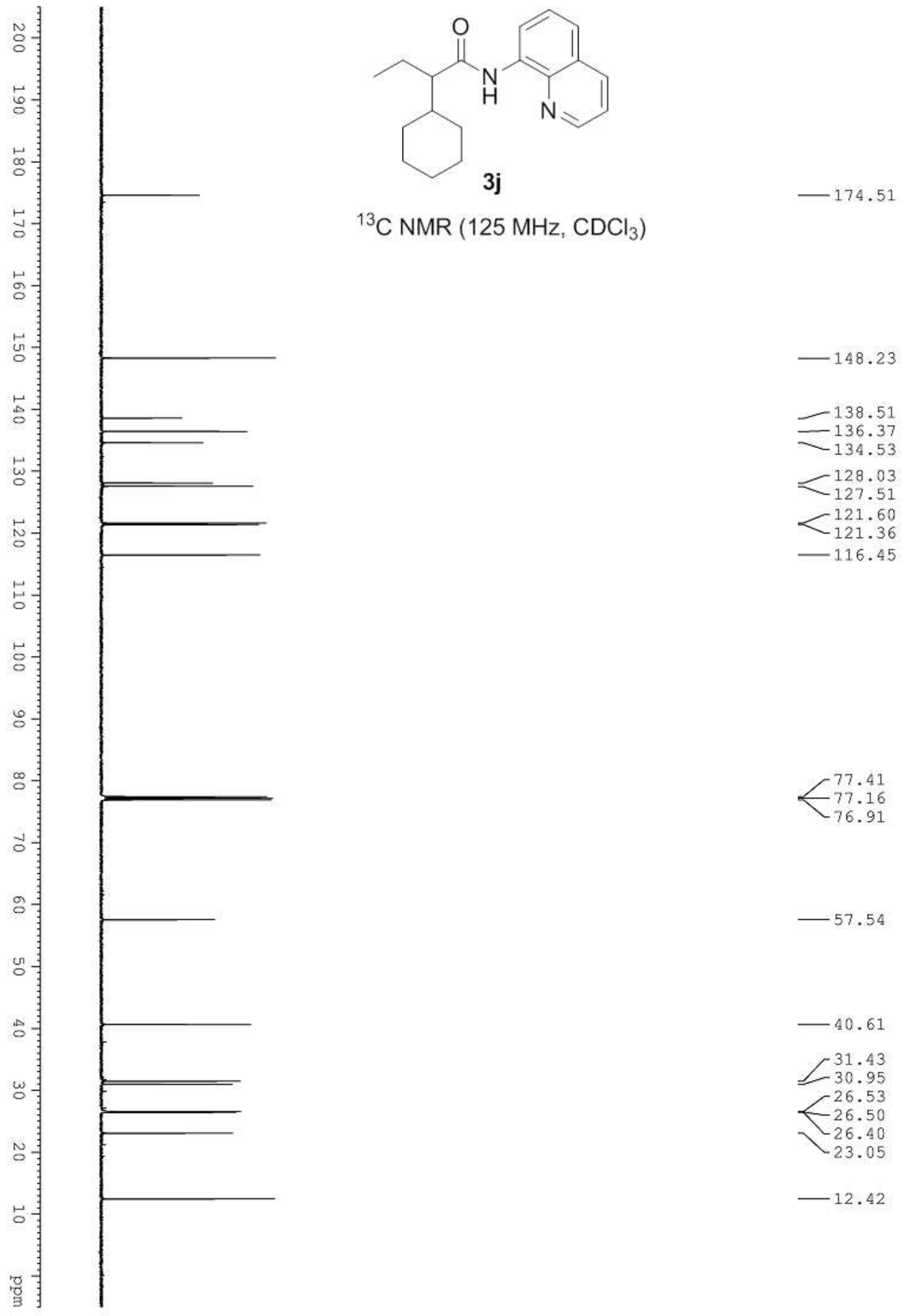
3j
^{13}C NMR (125 MHz, CDCl$_3$)
174.51
148.23
138.51
136.37
134.53
128.03
127.51
121.60
121.36
116.45
77.41
77.16
76.91
57.54
40.61
31.43
30.95
26.53
26.50
26.40
23.05
12.42
200
190
180
170
160
150
140
130
120
110
100
90
80
70
60
50
40
30
20
10
ppm

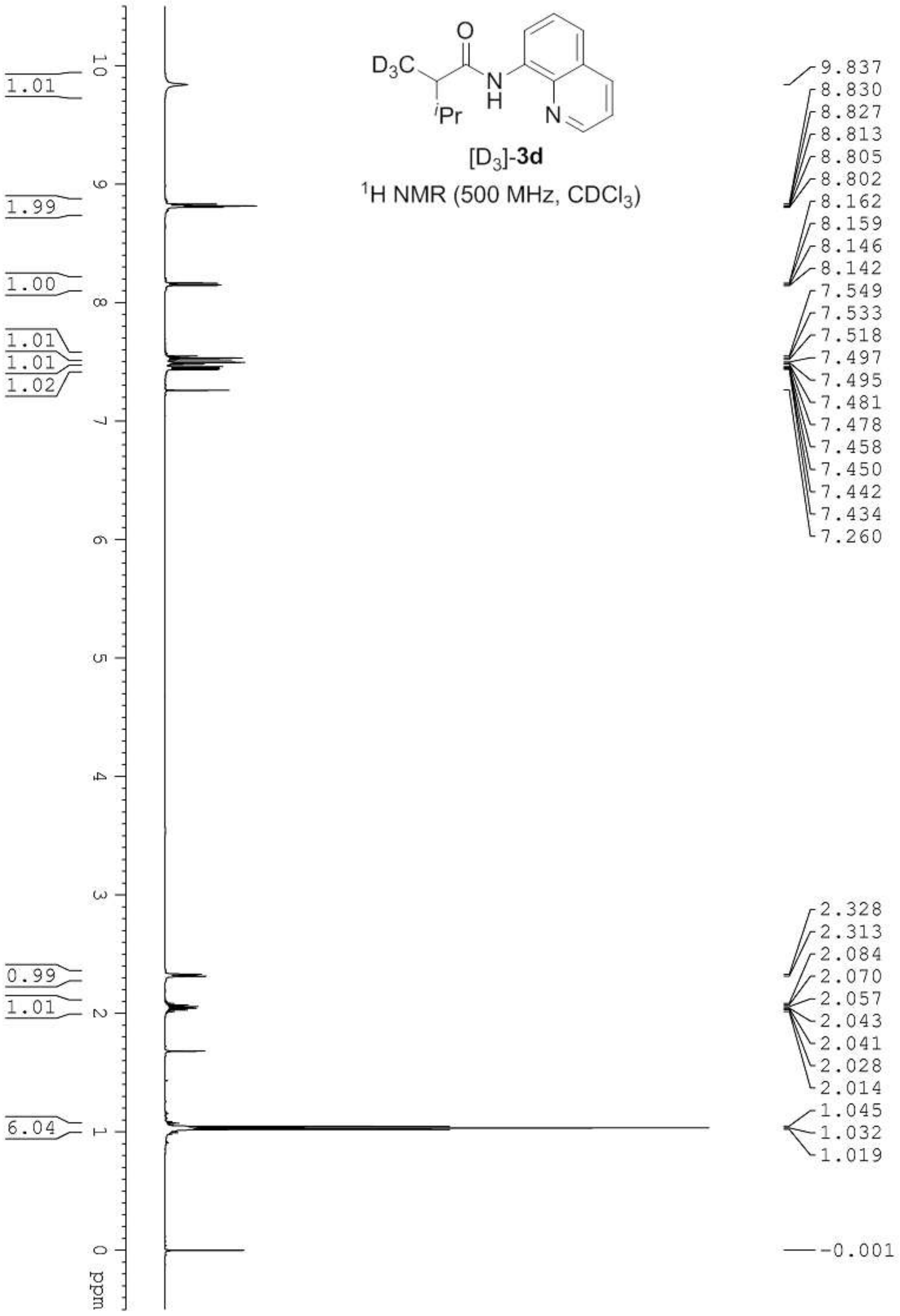

D3C
O
N
H
iPr
N
[D3]-3d
1H NMR (500 MHz, CDCl3)
9.837
8.830
8.827
8.813
8.805
8.802
8.162
8.159
8.146
8.142
7.549
7.533
7.518
7.497
7.495
7.481
7.478
7.458
7.450
7.442
7.434
7.260
2.328
2.313
2.084
2.070
2.057
2.043
2.041
2.028
2.014
1.045
1.032
1.019
-0.001
1.01
1.99
1.00
1.01
1.01
1.02
0.99
1.01
6.04
ppm

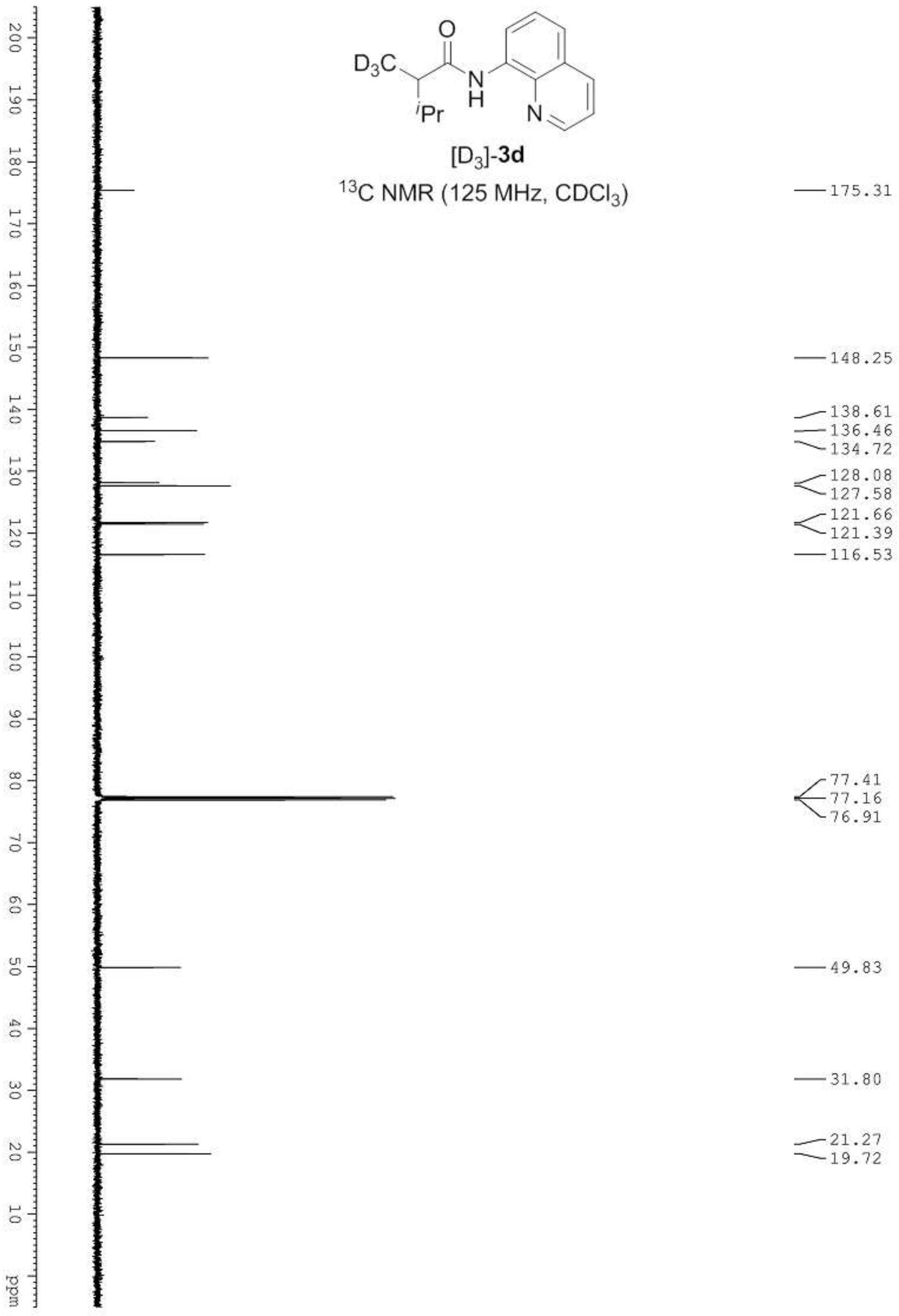
D_3C
O
N
H
^{i}Pr
N
$[D_3]$-**3d**
^{13}C NMR (125 MHz, $CDCl_3$)
175.31
148.25
138.61
136.46
134.72
128.08
127.58
121.66
121.39
116.53
77.41
77.16
76.91
49.83
31.80
21.27
19.72
200
190
180
170
160
150
140
130
120
110
100
90
80
70
60
50
40
30
20
10
ppm

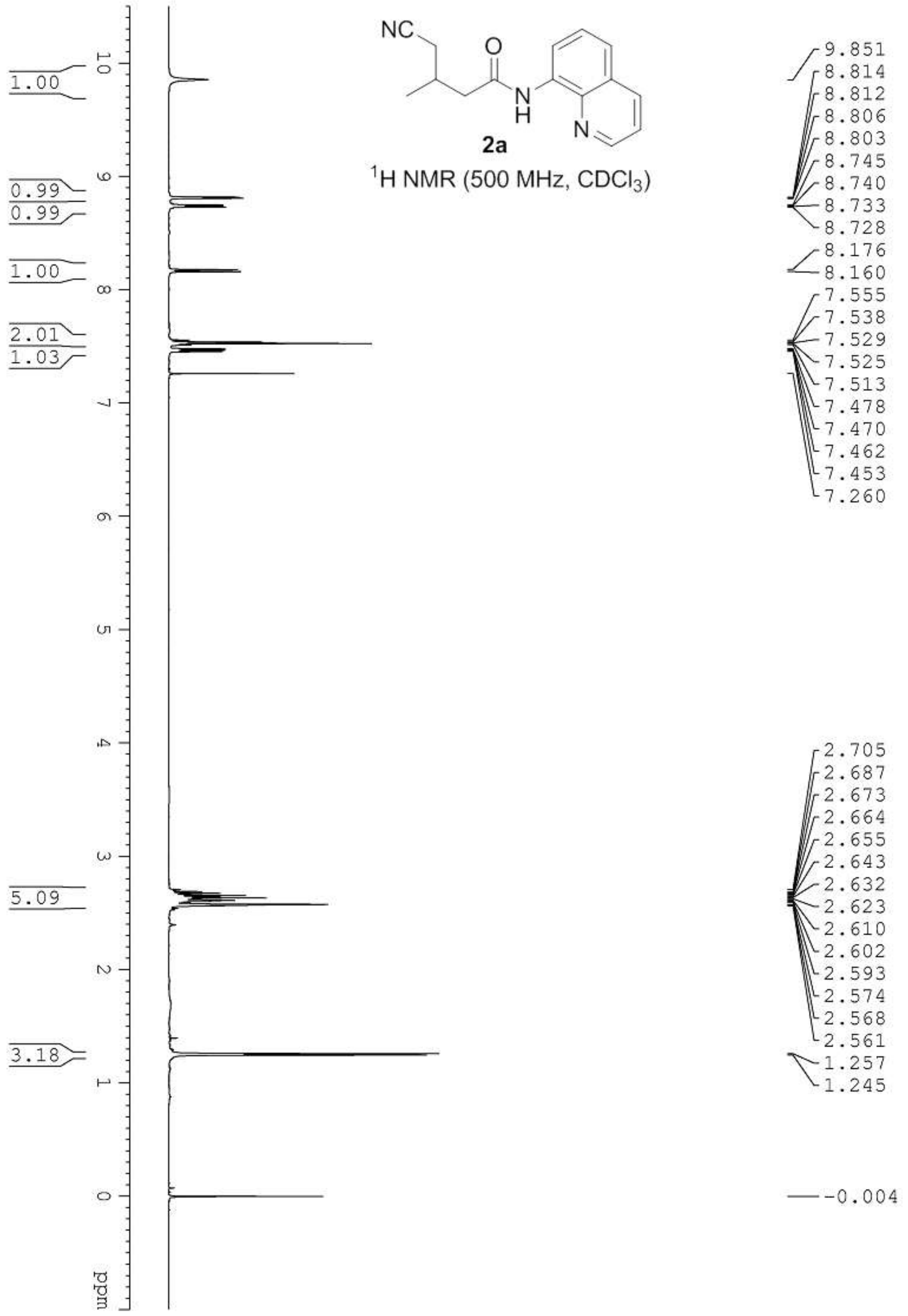
NC
O
N
H
N
2a
1H NMR (500 MHz, CDCl3)
1.00
0.99
0.99
1.00
2.01
1.03
5.09
3.18
10
9
8
7
6
5
4
3
2
1
0
ppm
9.851
8.814
8.812
8.806
8.803
8.745
8.740
8.733
8.728
8.176
8.160
7.555
7.538
7.529
7.525
7.513
7.478
7.470
7.462
7.453
7.260
2.705
2.687
2.673
2.664
2.655
2.643
2.632
2.623
2.610
2.602
2.593
2.574
2.568
2.561
1.257
1.245
-0.004

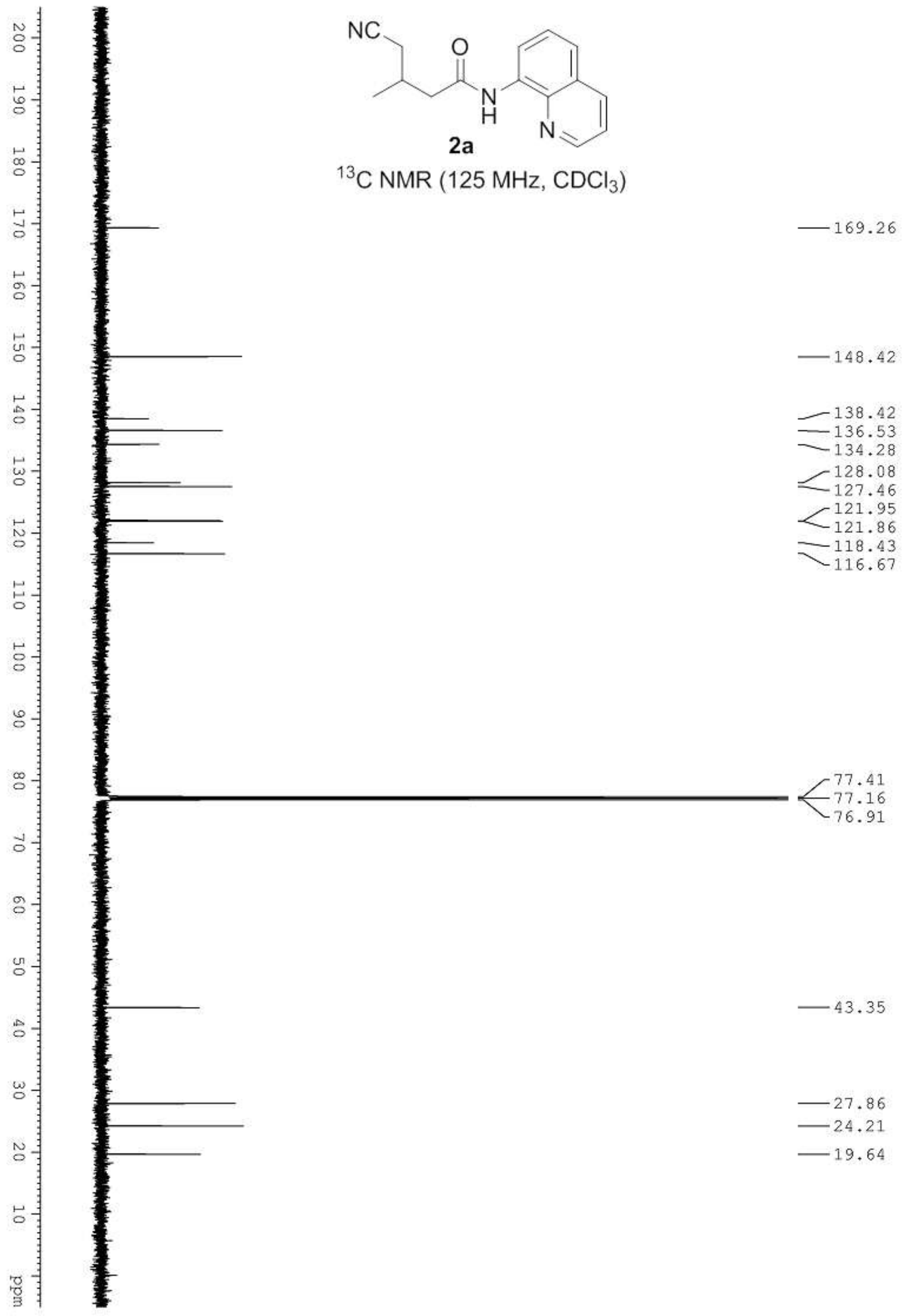
NC
O
N
H
N
2a
^{13}C NMR (125 MHz, CDCl$_3$)
169.26
148.42
138.42
136.53
134.28
128.08
127.46
121.95
121.86
118.43
116.67
77.41
77.16
76.91
43.35
27.86
24.21
19.64
200
190
180
170
160
150
140
130
120
110
100
90
80
70
60
50
40
30
20
10
ppm

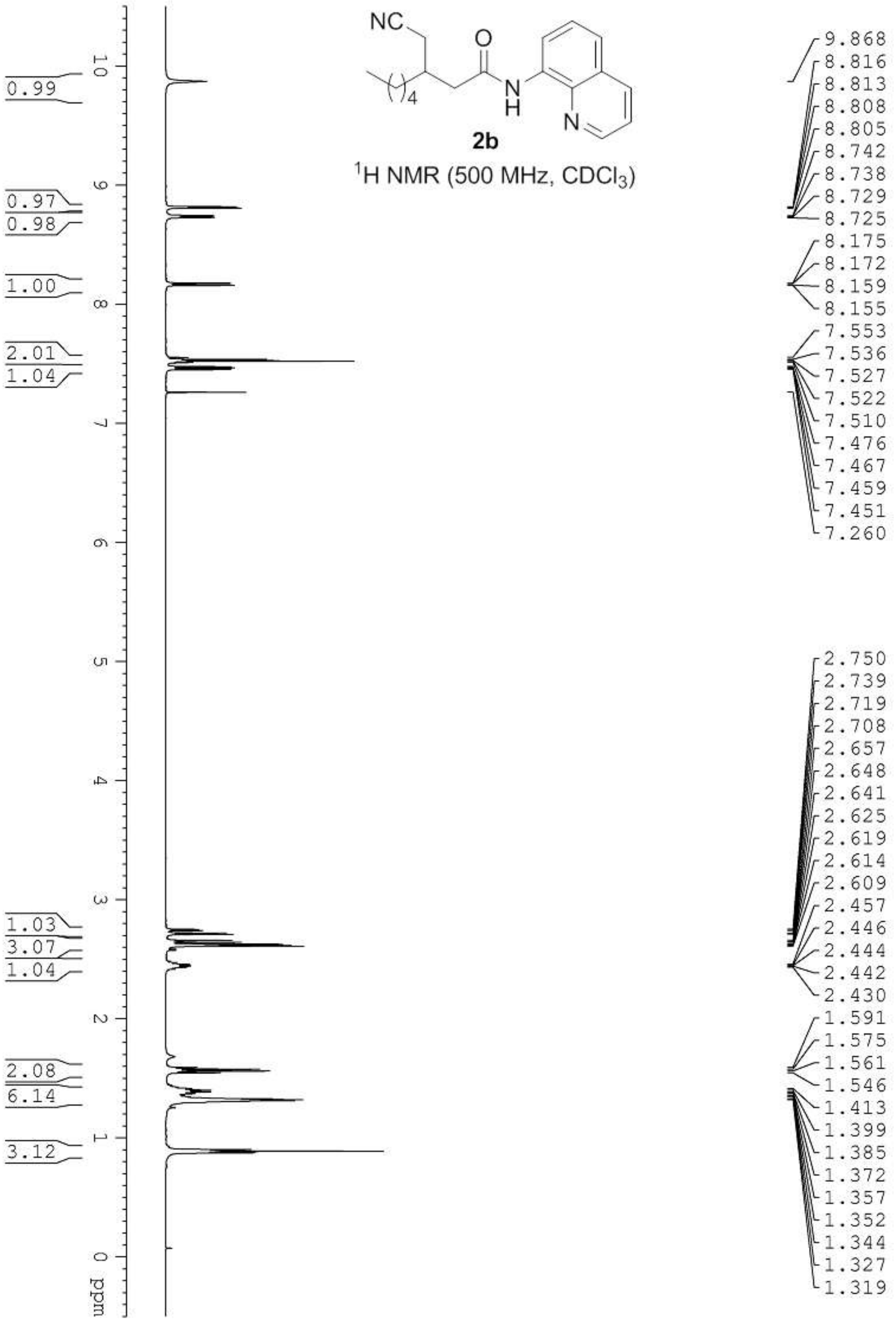
NC
O
N
H
N
4
2b
1H NMR (500 MHz, CDCl3)
9.868
8.816
8.813
8.808
8.805
8.742
8.738
8.729
8.725
8.175
8.172
8.159
8.155
7.553
7.536
7.527
7.522
7.510
7.476
7.467
7.459
7.451
7.260
2.750
2.739
2.719
2.708
2.657
2.648
2.641
2.625
2.619
2.614
2.609
2.457
2.446
2.444
2.442
2.430
1.591
1.575
1.561
1.546
1.413
1.399
1.385
1.372
1.357
1.352
1.344
1.327
1.319
0.99
0.97
0.98
1.00
2.01
1.04
1.03
3.07
1.04
2.08
6.14
3.12
10
9
8
7
6
5
4
3
2
1
0
ppm

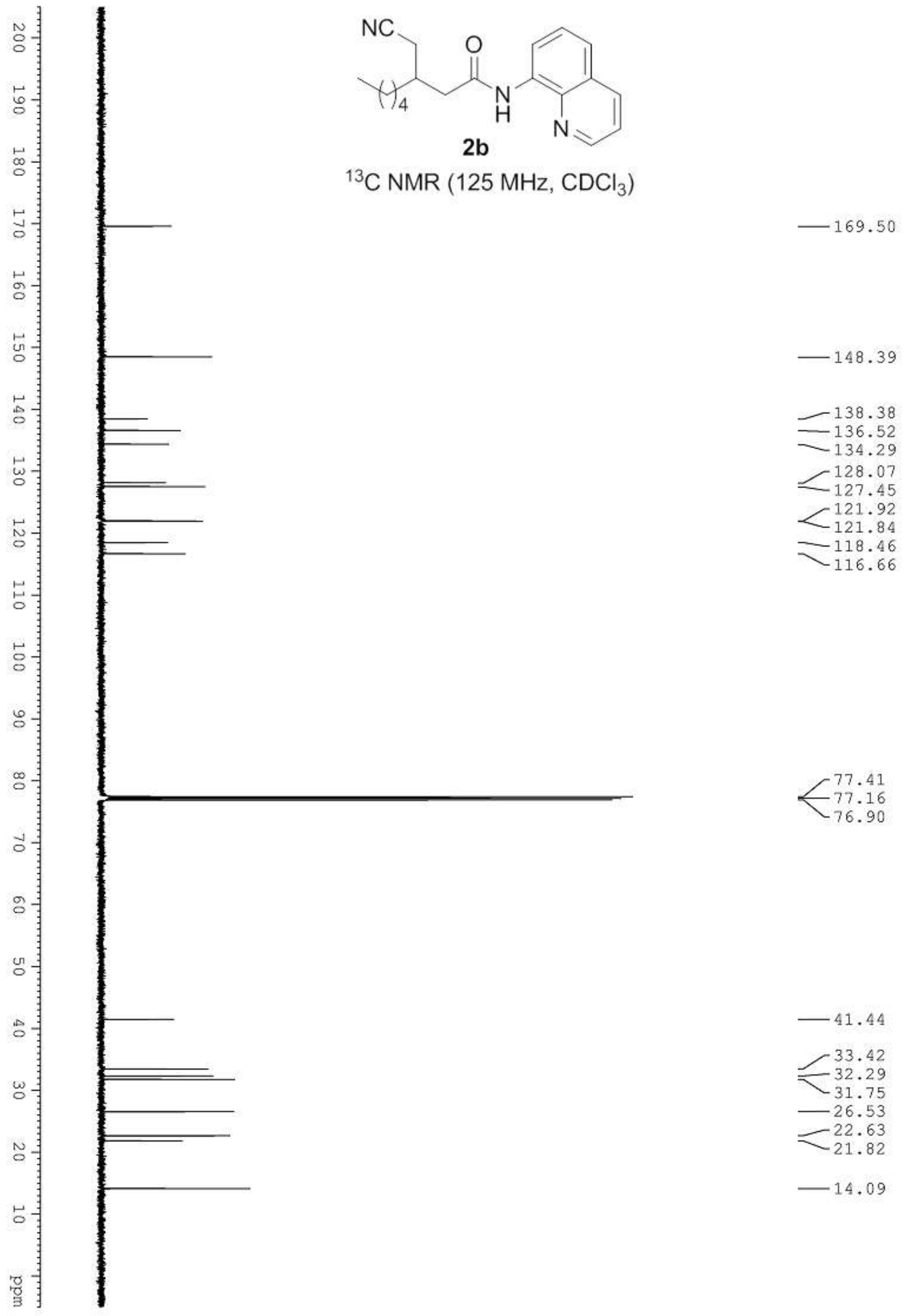

2b

^{13}C NMR (125 MHz, $CDCl_3$)

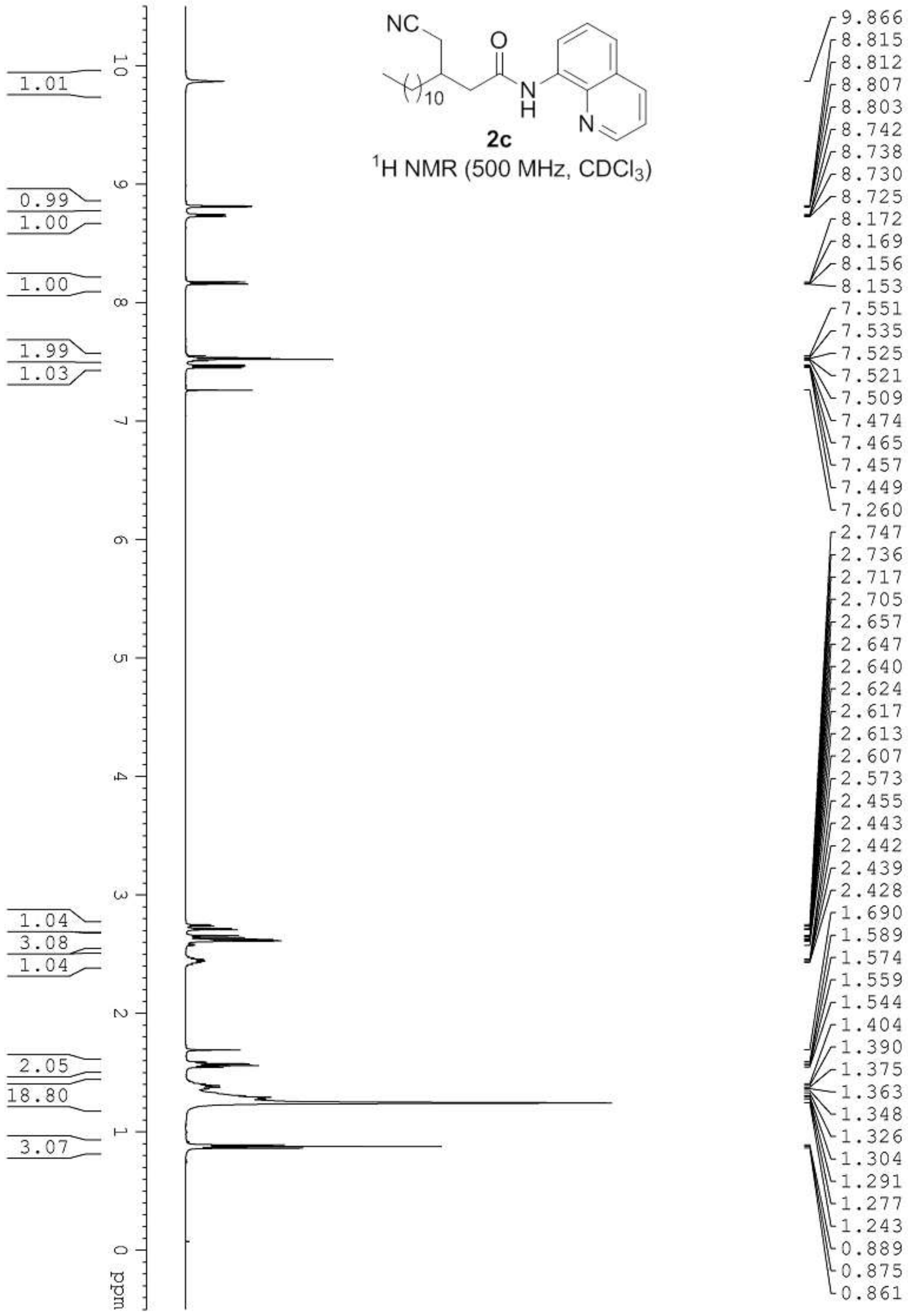
NC
O
N
H
N
10
2c
¹H NMR (500 MHz, CDCl₃)
1.01
0.99
1.00
1.00
1.99
1.03
1.04
3.08
1.04
2.05
18.80
3.07
10
9
8
7
6
5
4
3
2
1
0 ppm
9.866
8.815
8.812
8.807
8.803
8.742
8.738
8.730
8.725
8.172
8.169
8.156
8.153
7.551
7.535
7.525
7.521
7.509
7.474
7.465
7.457
7.449
7.260
2.747
2.736
2.717
2.705
2.657
2.647
2.640
2.624
2.617
2.613
2.607
2.573
2.455
2.443
2.442
2.439
2.428
1.690
1.589
1.574
1.559
1.544
1.404
1.390
1.375
1.363
1.348
1.326
1.304
1.291
1.277
1.243
0.889
0.875
0.861

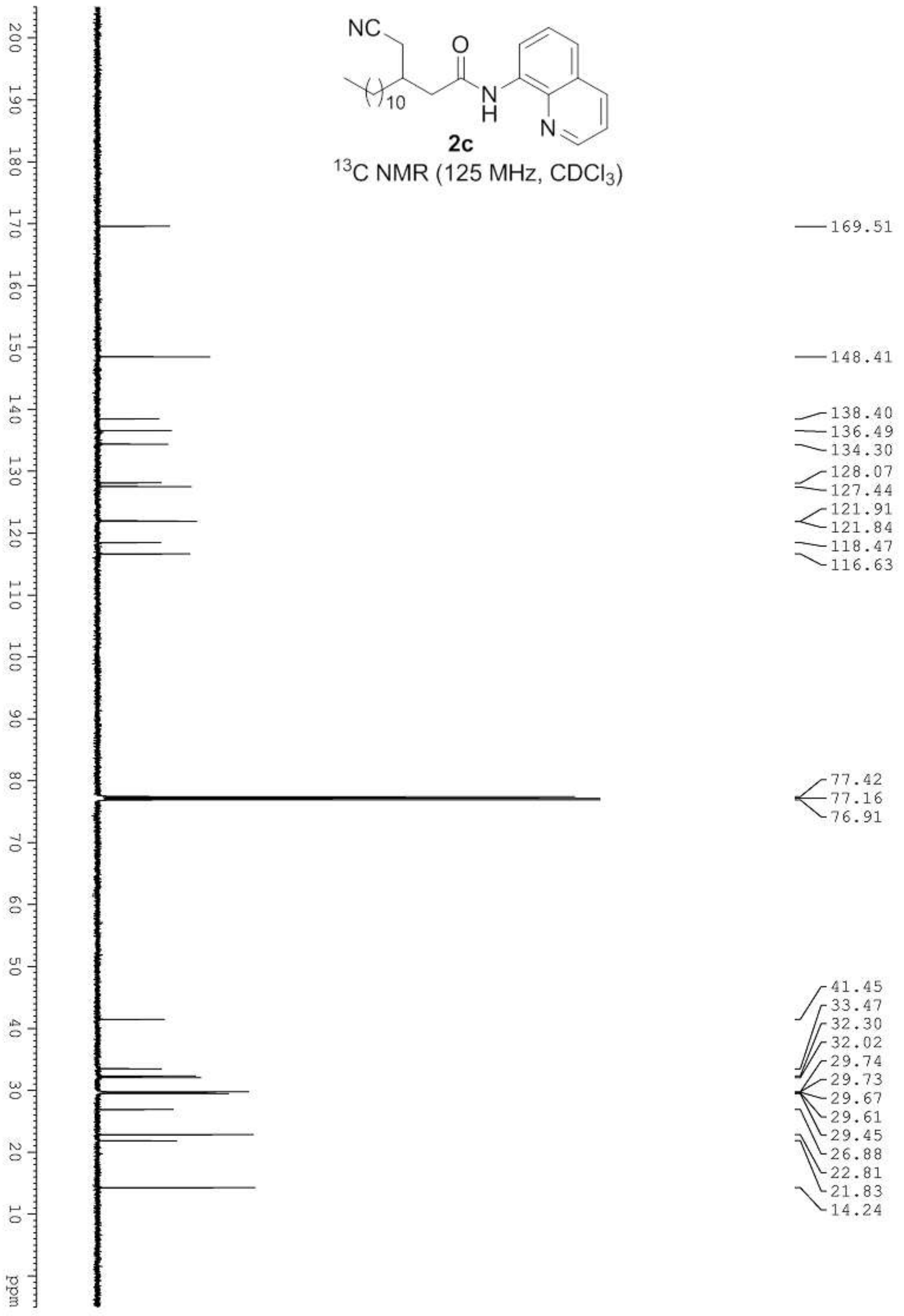
2c
^{13}C NMR (125 MHz, $CDCl_3$)
169.51
148.41
138.40
136.49
134.30
128.07
127.44
121.91
121.84
118.47
116.63
77.42
77.16
76.91
41.45
33.47
32.30
32.02
29.74
29.73
29.67
29.61
29.45
26.88
22.81
21.83
14.24
200
190
180
170
160
150
140
130
120
110
100
90
80
70
60
50
40
30
20
10
ppm

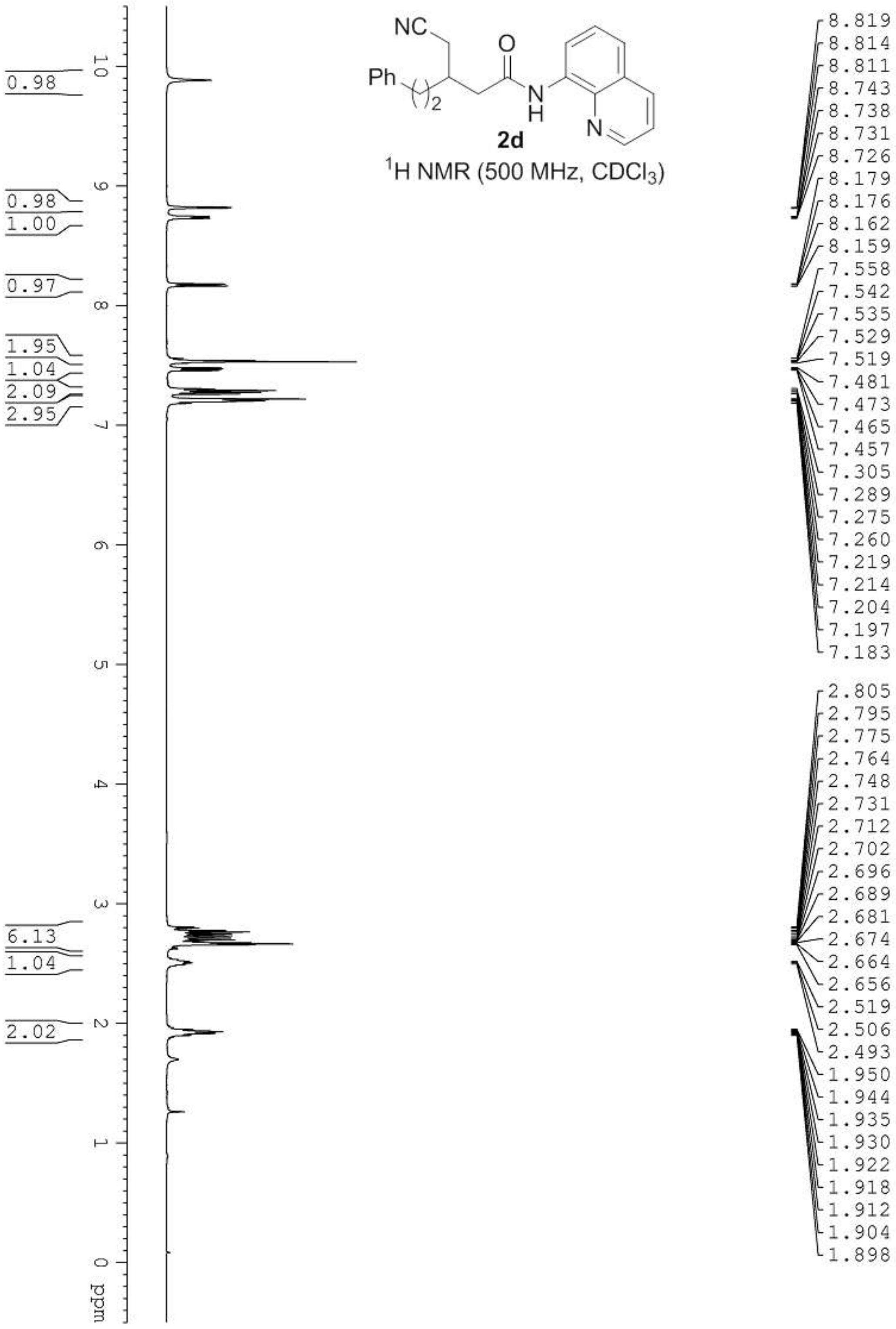
NC
O
Ph
2
N
H
N
2d
^{1}H NMR (500 MHz, CDCl$_3$)
8.819
8.814
8.811
8.743
8.738
8.731
8.726
8.179
8.176
8.162
8.159
7.558
7.542
7.535
7.529
7.519
7.481
7.473
7.465
7.457
7.305
7.289
7.275
7.260
7.219
7.214
7.204
7.197
7.183
2.805
2.795
2.775
2.764
2.748
2.731
2.712
2.702
2.696
2.689
2.681
2.674
2.664
2.656
2.519
2.506
2.493
1.950
1.944
1.935
1.930
1.922
1.918
1.912
1.904
1.898
0.98
0.98
1.00
0.97
1.95
1.04
2.09
2.95
6.13
1.04
2.02
ppm

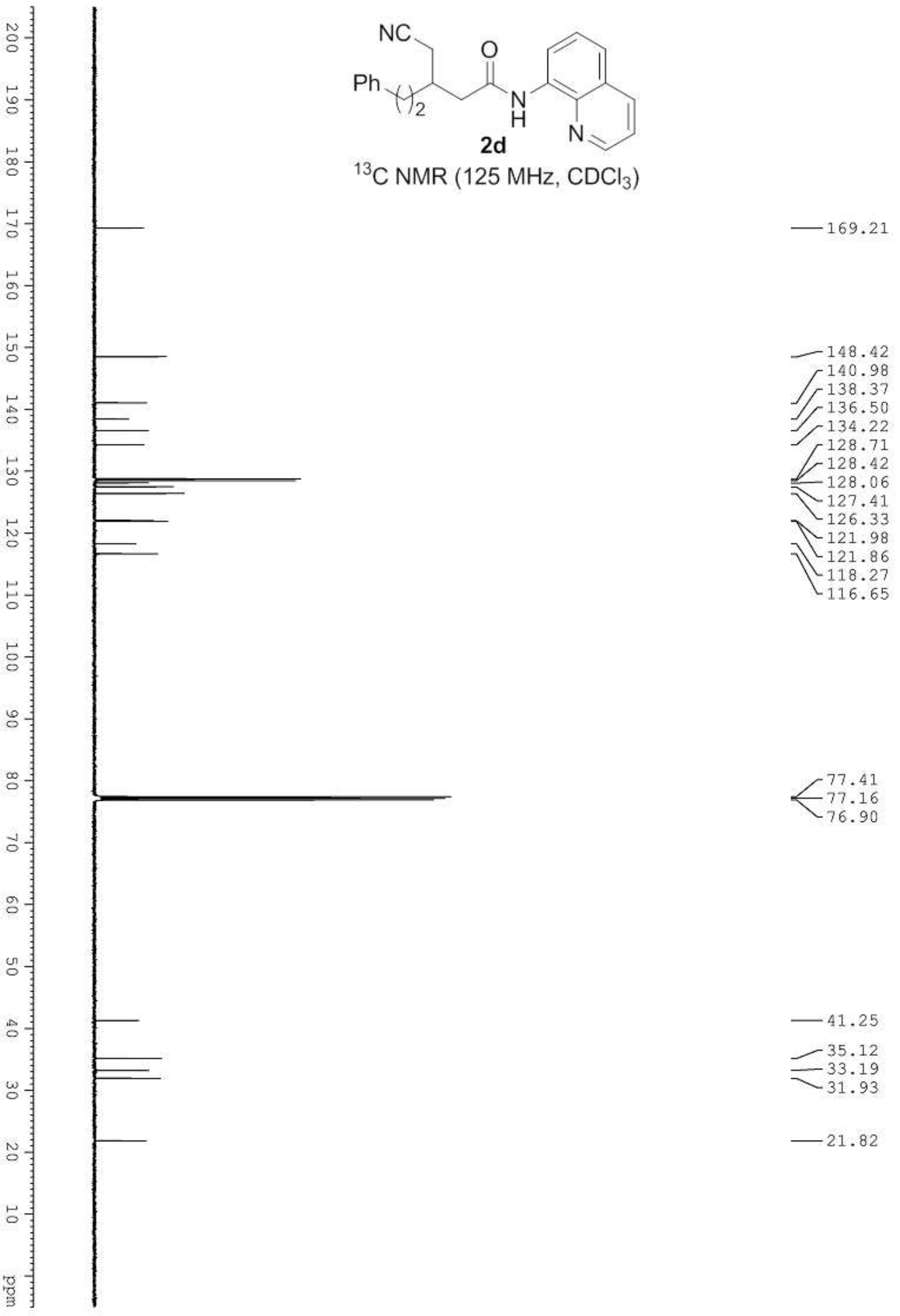
NC
O
Ph
2
N
H
N
2d
^{13}C NMR (125 MHz, CDCl$_3$)
169.21
148.42
140.98
138.37
136.50
134.22
128.71
128.42
128.06
127.41
126.33
121.98
121.86
118.27
116.65
77.41
77.16
76.90
41.25
35.12
33.19
31.93
21.82
200
190
180
170
160
150
140
130
120
110
100
90
80
70
60
50
40
30
20
10
ppm

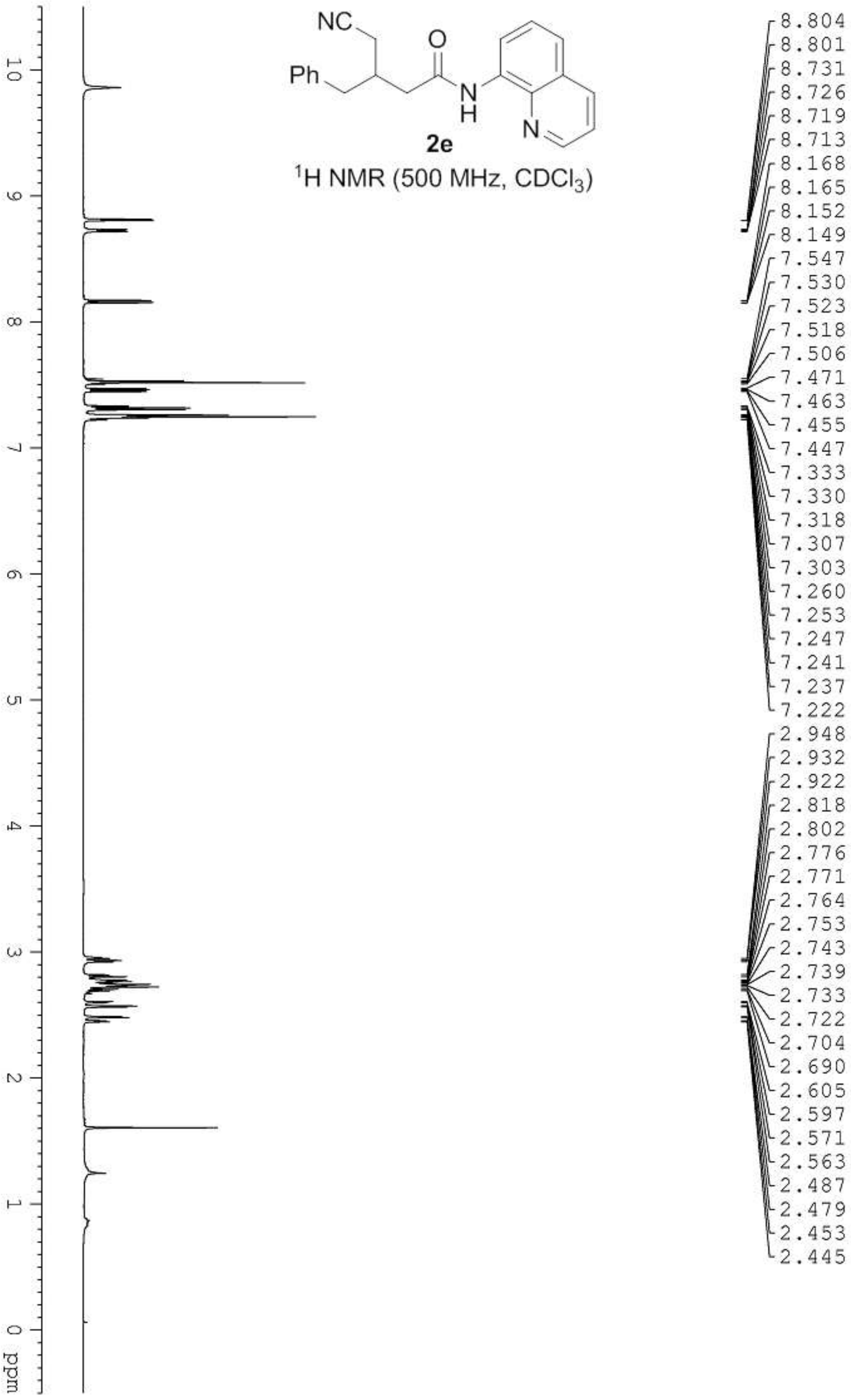

2e

^{1}H NMR (500 MHz, $CDCl_3$)

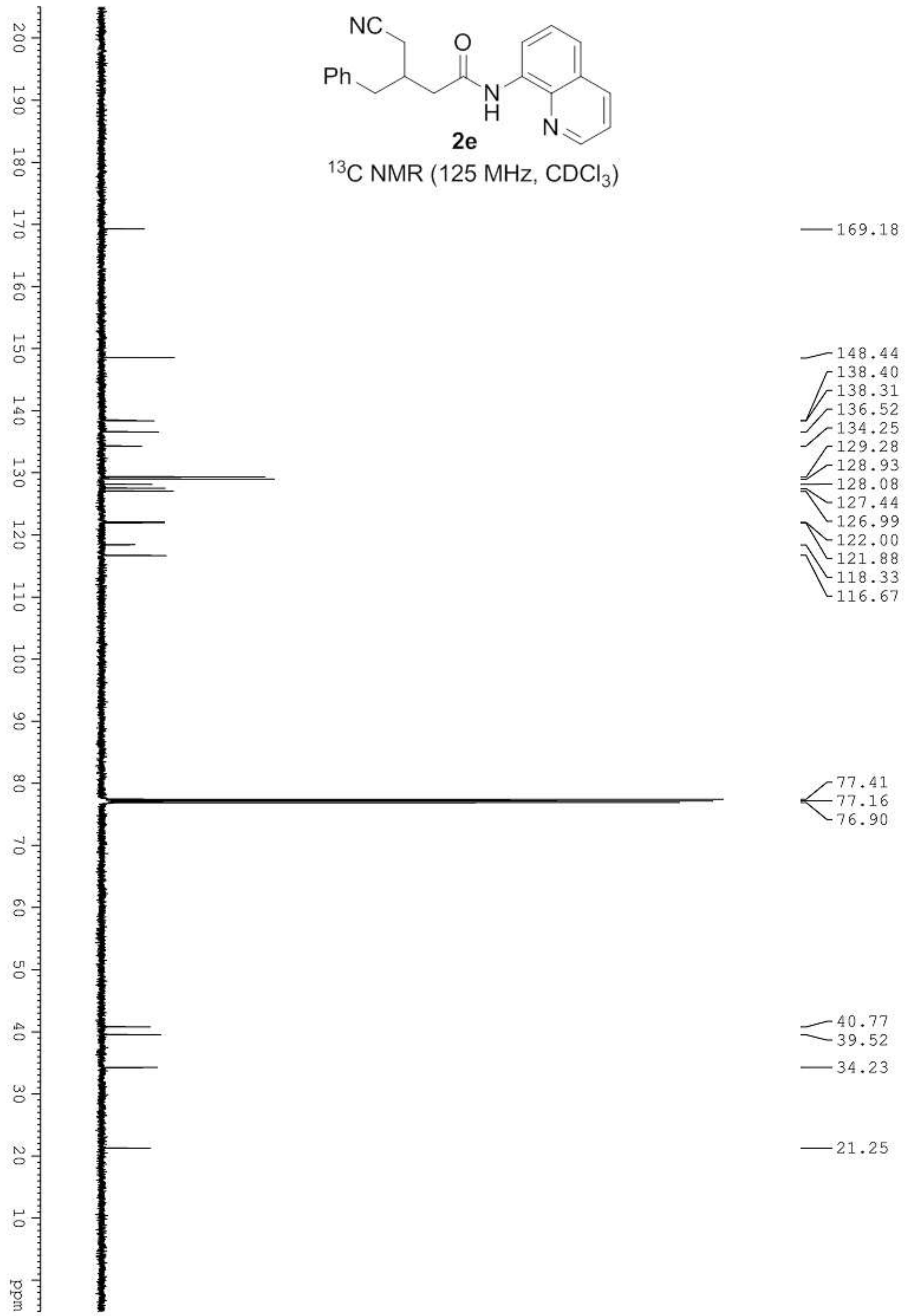

2e

^{13}C NMR (125 MHz, $CDCl_3$)

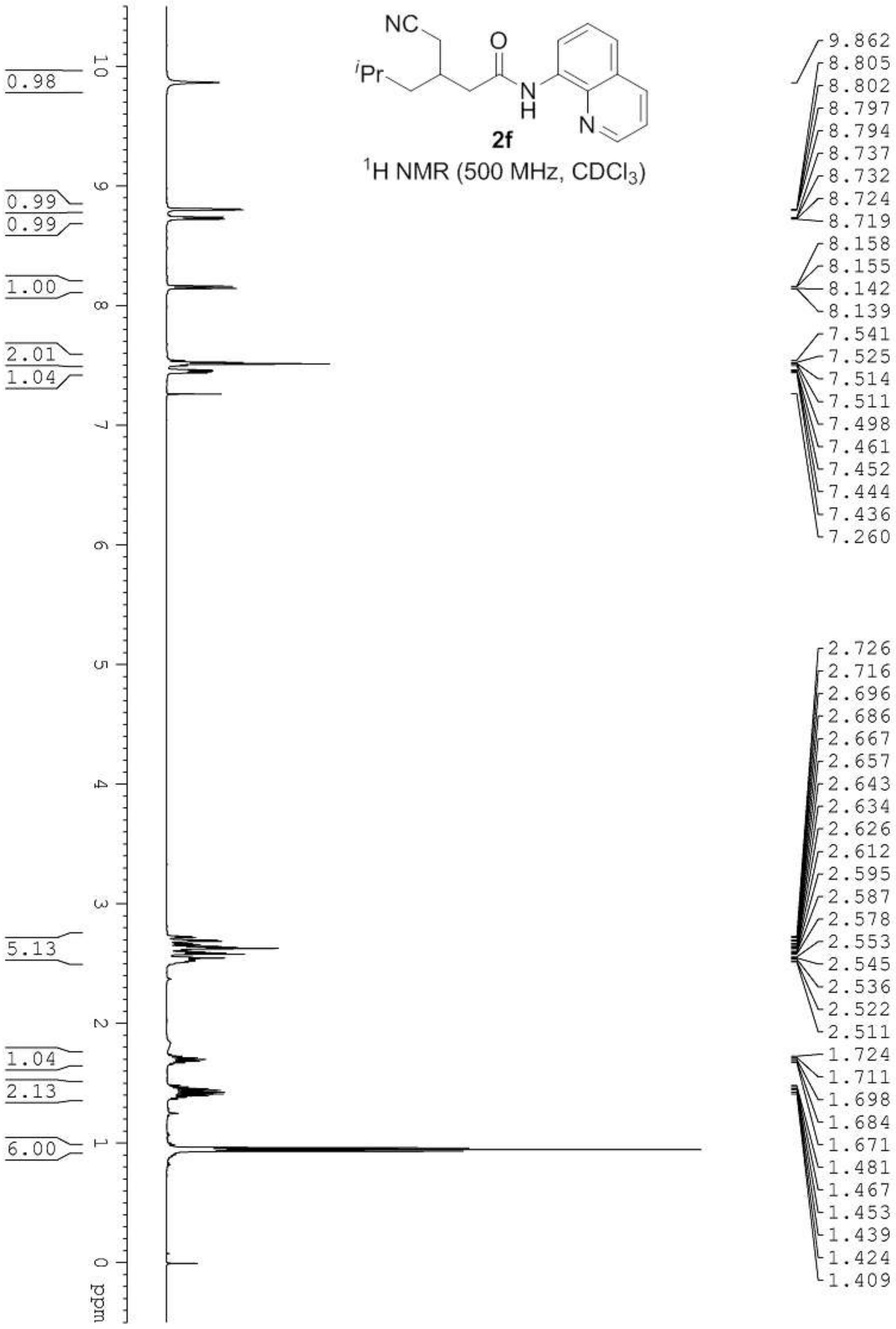

2f

^{1}H NMR (500 MHz, $CDCl_3$)

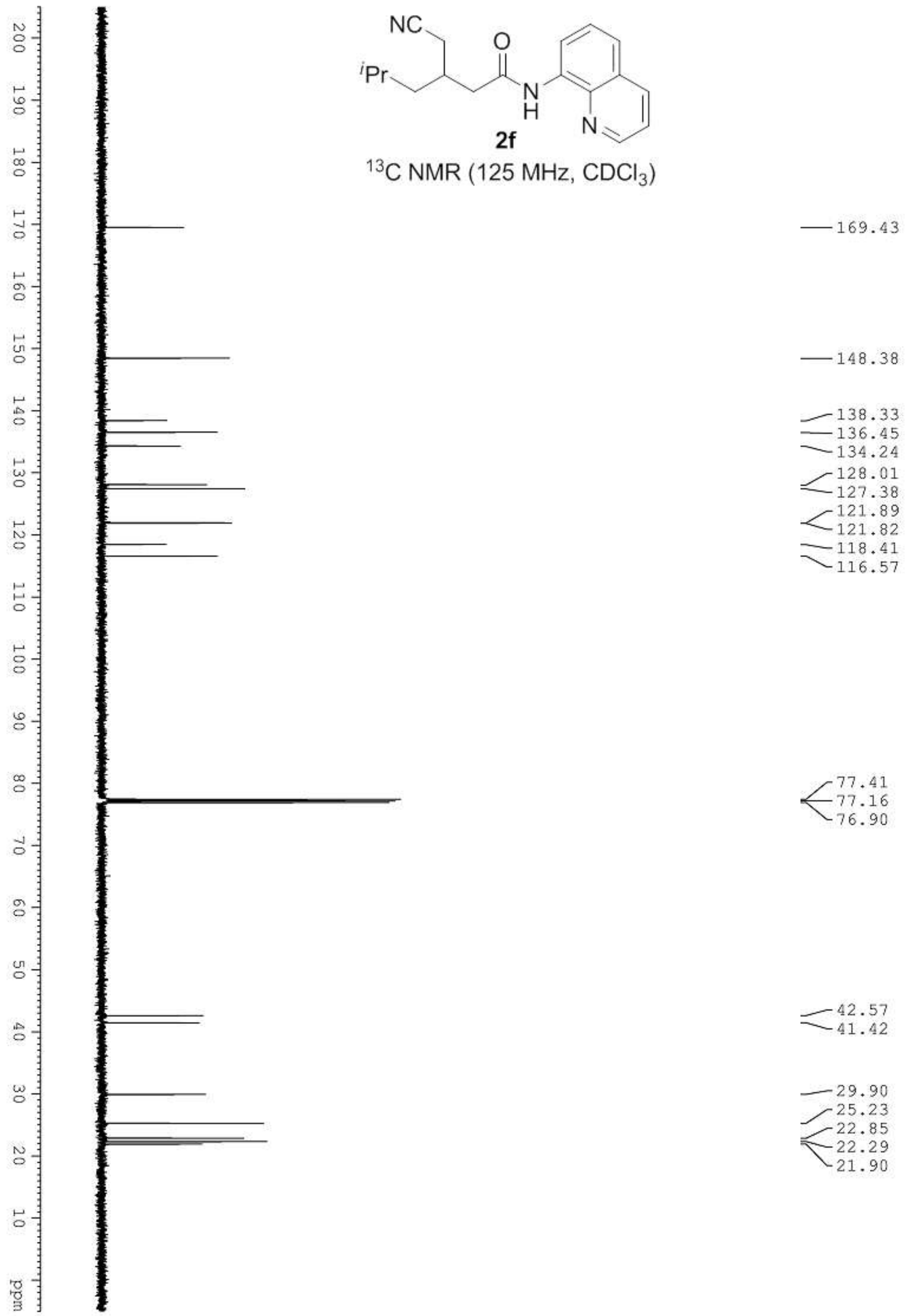
NC
O
iPr
N
H
N
2f
13C NMR (125 MHz, CDCl3)
169.43
148.38
138.33
136.45
134.24
128.01
127.38
121.89
121.82
118.41
116.57
77.41
77.16
76.90
42.57
41.42
29.90
25.23
22.85
22.29
21.90
200
190
180
170
160
150
140
130
120
110
100
90
80
70
60
50
40
30
20
10
ppm

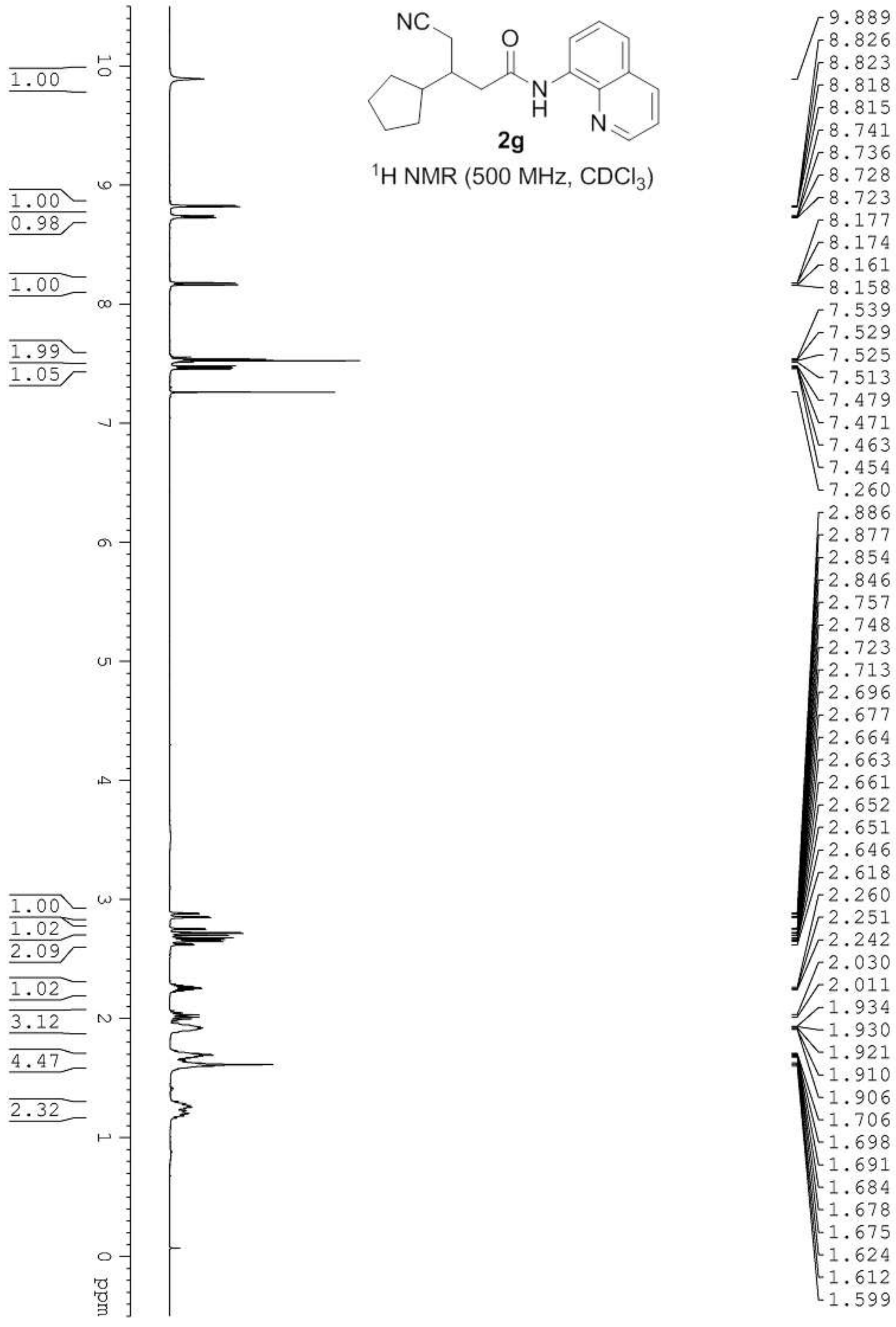
NC
O
N
H
N
2g
^{1}H NMR (500 MHz, CDCl$_3$)
1.00
1.00
0.98
1.00
1.99
1.05
1.00
1.02
2.09
1.02
3.12
4.47
2.32
10
9
8
7
6
5
4
3
2
1
0 ppm
9.889
8.826
8.823
8.818
8.815
8.741
8.736
8.728
8.723
8.177
8.174
8.161
8.158
7.539
7.529
7.525
7.513
7.479
7.471
7.463
7.454
7.260
2.886
2.877
2.854
2.846
2.757
2.748
2.723
2.713
2.696
2.677
2.664
2.663
2.661
2.652
2.651
2.646
2.618
2.260
2.251
2.242
2.030
2.011
1.934
1.930
1.921
1.910
1.906
1.706
1.698
1.691
1.684
1.678
1.675
1.624
1.612
1.599

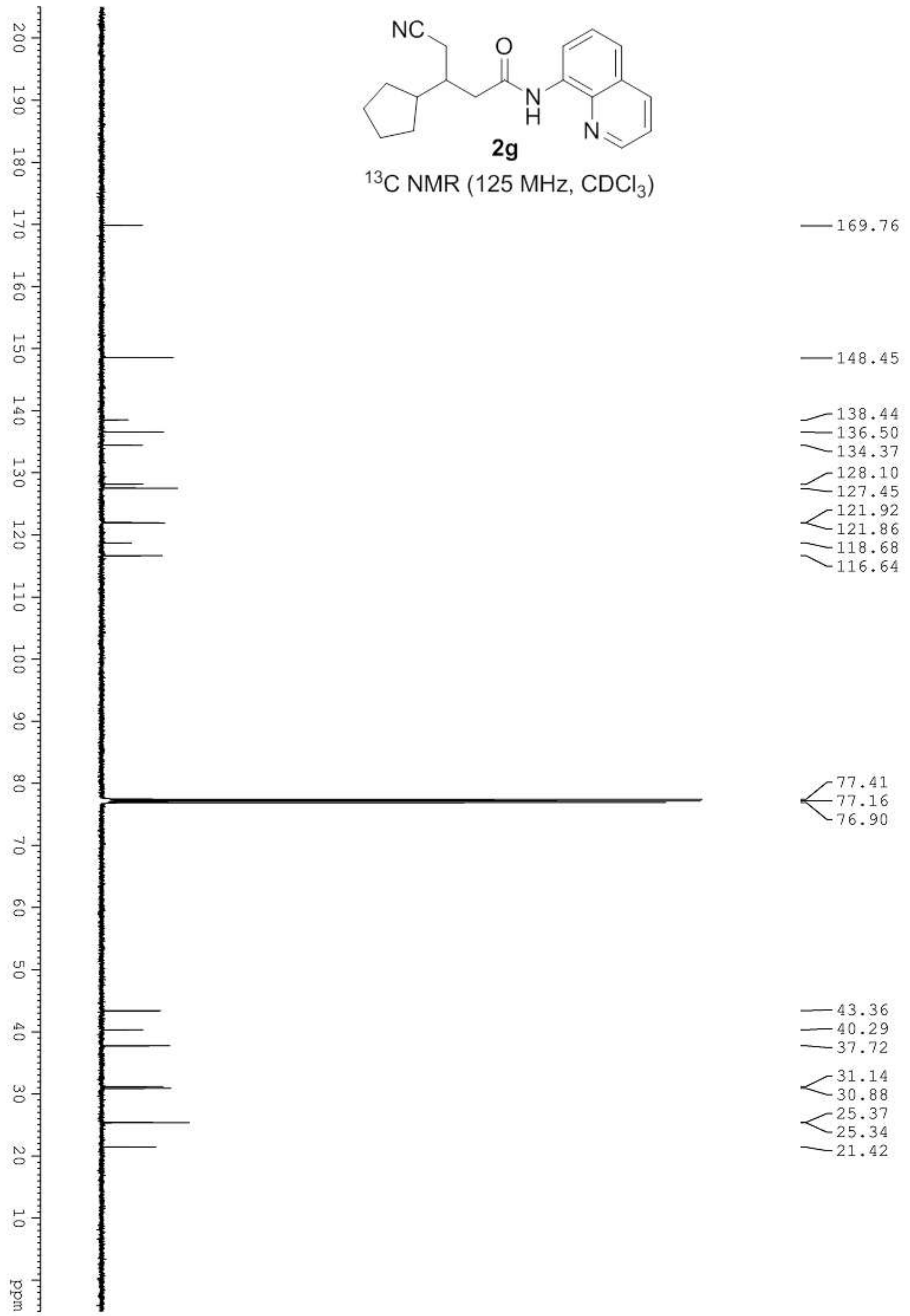
NC
O
N
H
N
2g
^{13}C NMR (125 MHz, CDCl$_3$)
169.76
148.45
138.44
136.50
134.37
128.10
127.45
121.92
121.86
118.68
116.64
77.41
77.16
76.90
43.36
40.29
37.72
31.14
30.88
25.37
25.34
21.42
200
190
180
170
160
150
140
130
120
110
100
90
80
70
60
50
40
30
20
10
ppm

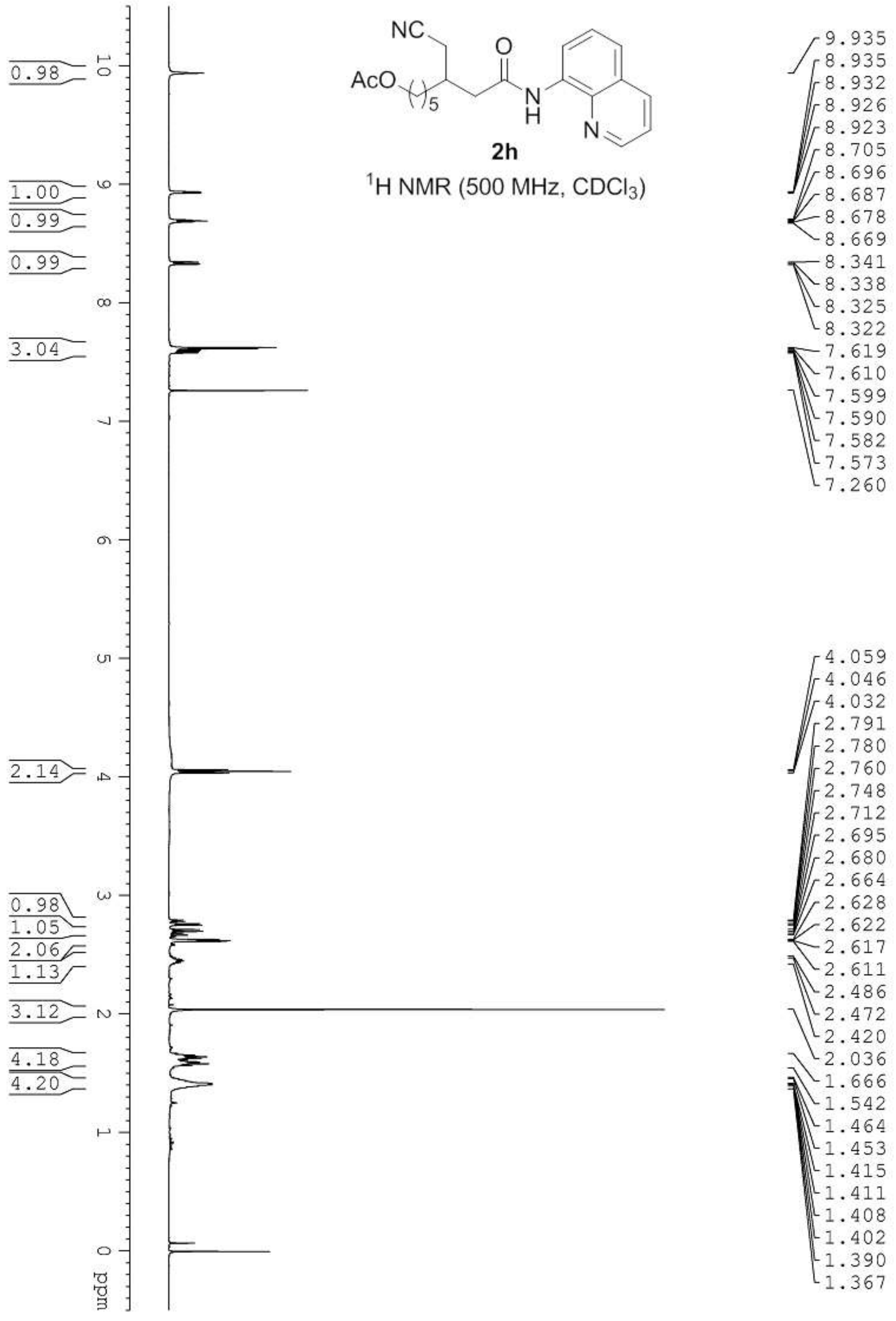
NC
AcO
5
O
N
H
N
2h
1H NMR (500 MHz, CDCl3)
9.935
8.935
8.932
8.926
8.923
8.705
8.696
8.687
8.678
8.669
8.341
8.338
8.325
8.322
7.619
7.610
7.599
7.590
7.582
7.573
7.260
4.059
4.046
4.032
2.791
2.780
2.760
2.748
2.712
2.695
2.680
2.664
2.628
2.622
2.617
2.611
2.486
2.472
2.420
2.036
1.666
1.542
1.464
1.453
1.415
1.411
1.408
1.402
1.390
1.367
0.98
1.00
0.99
0.99
3.04
2.14
0.98
1.05
2.06
1.13
3.12
4.18
4.20
ppm

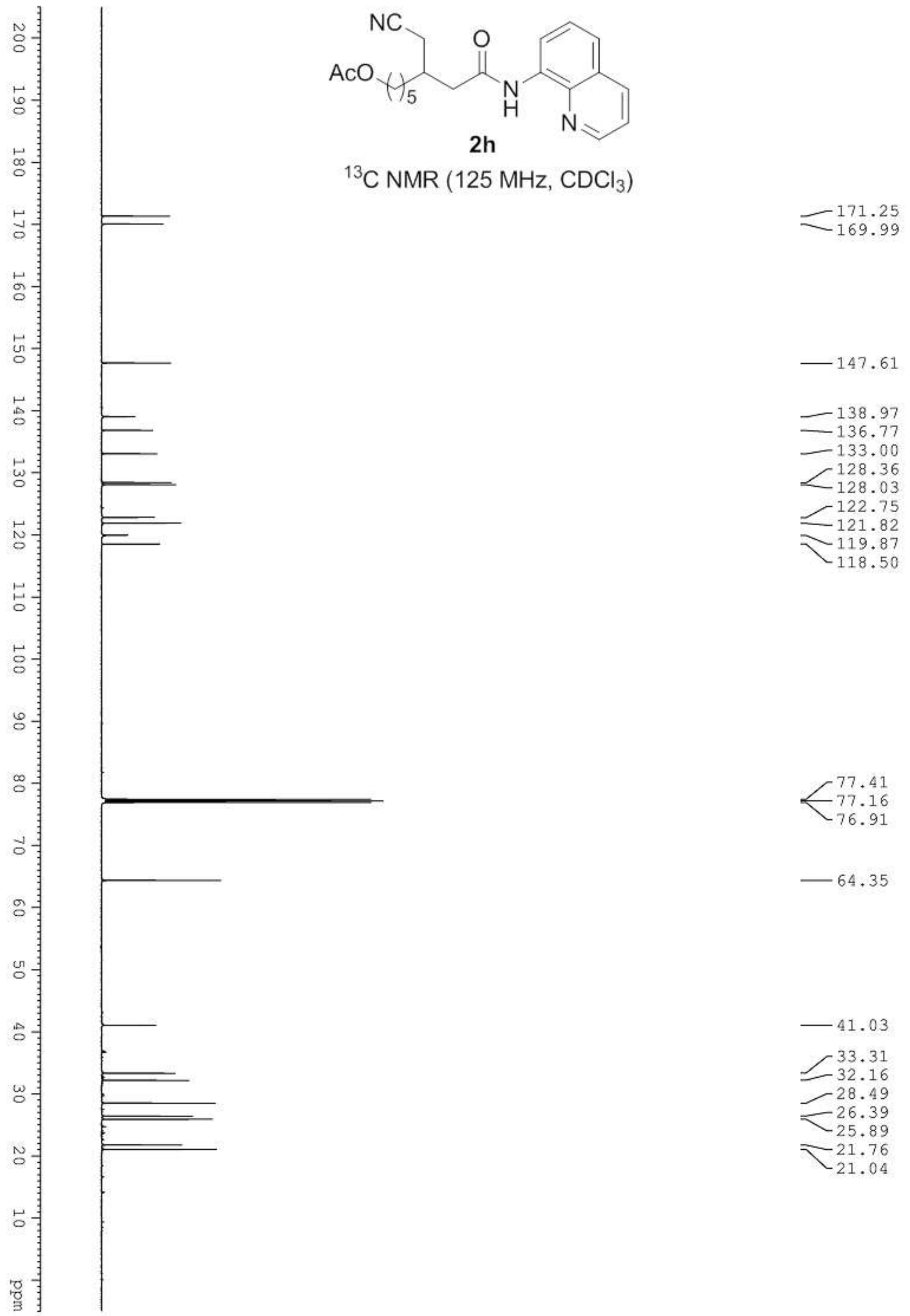

2h

^{13}C NMR (125 MHz, $CDCl_3$)

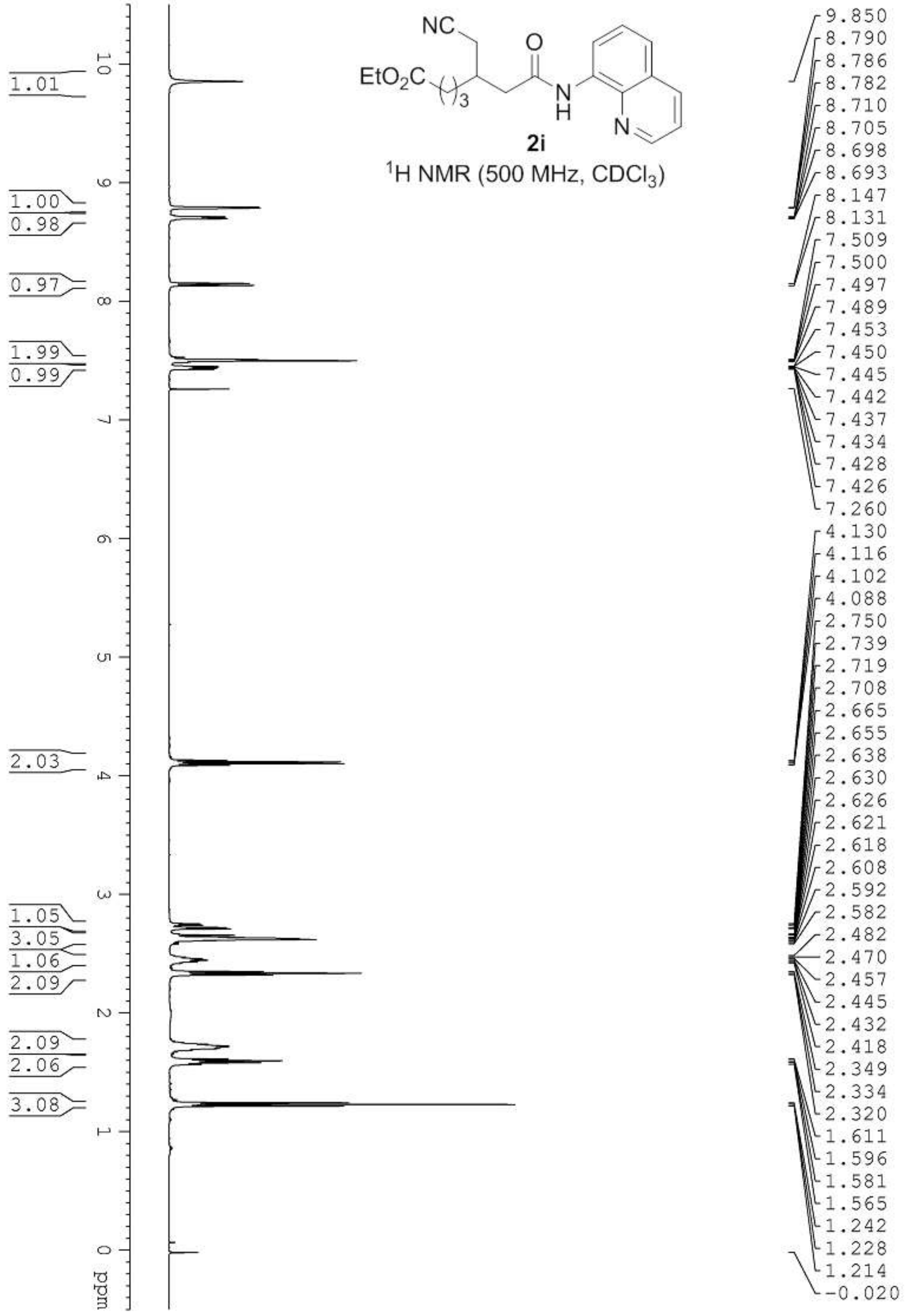
NC
EtO2C
3
O
N
H
N
2i
1H NMR (500 MHz, CDCl3)
9.850
8.790
8.786
8.782
8.710
8.705
8.698
8.693
8.147
8.131
7.509
7.500
7.497
7.489
7.453
7.450
7.445
7.442
7.437
7.434
7.428
7.426
7.260
4.130
4.116
4.102
4.088
2.750
2.739
2.719
2.708
2.665
2.655
2.638
2.630
2.626
2.621
2.618
2.608
2.592
2.582
2.482
2.470
2.457
2.445
2.432
2.418
2.349
2.334
2.320
1.611
1.596
1.581
1.565
1.242
1.228
1.214
-0.020
1.01
1.00
0.98
0.97
1.99
0.99
2.03
1.05
3.05
1.06
2.09
2.09
2.06
3.08
10
9
8
7
6
5
4
3
2
1
0
ppm

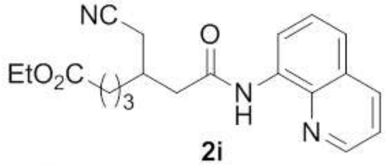

2i

^{13}C NMR (125 MHz, $CDCl_3$)

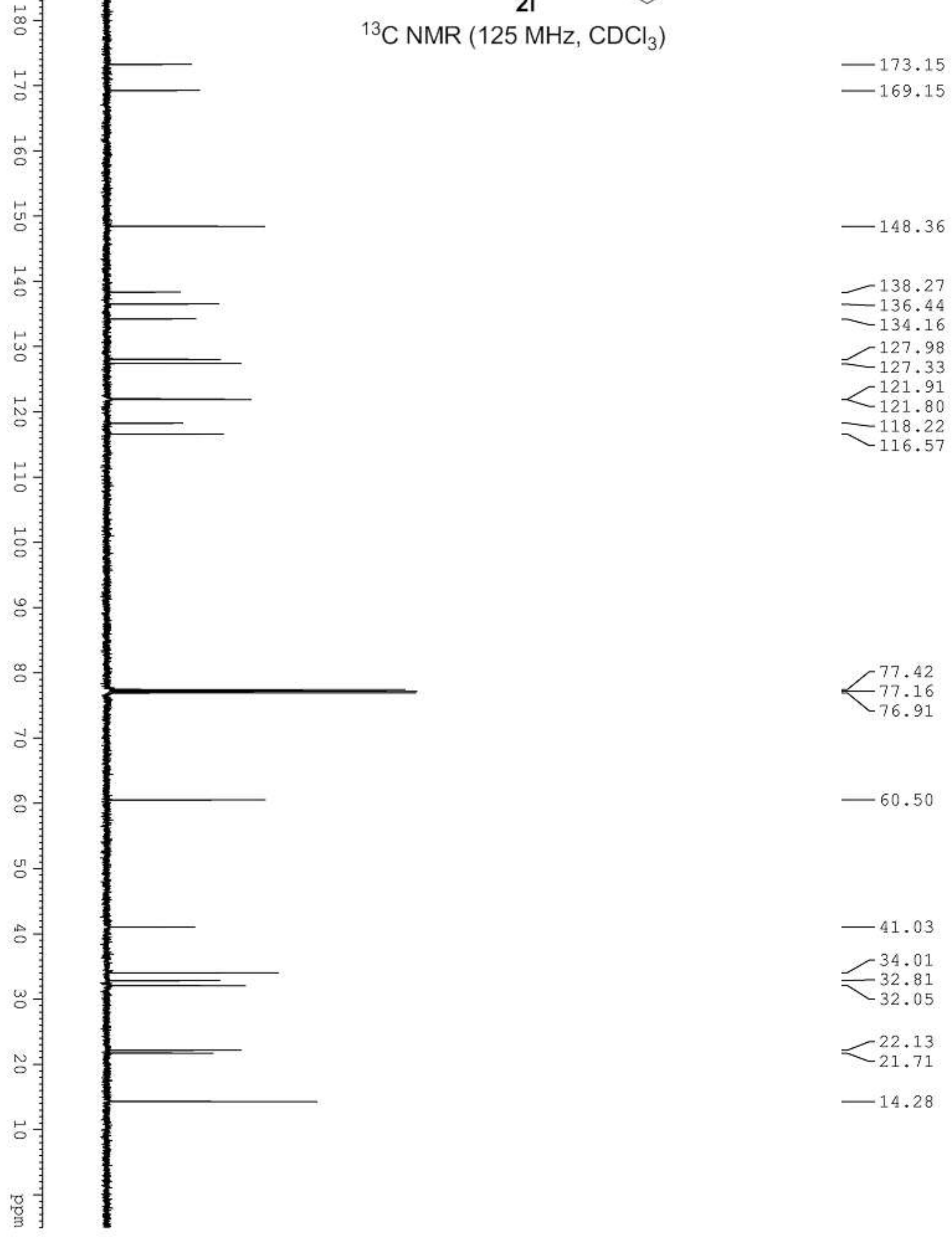

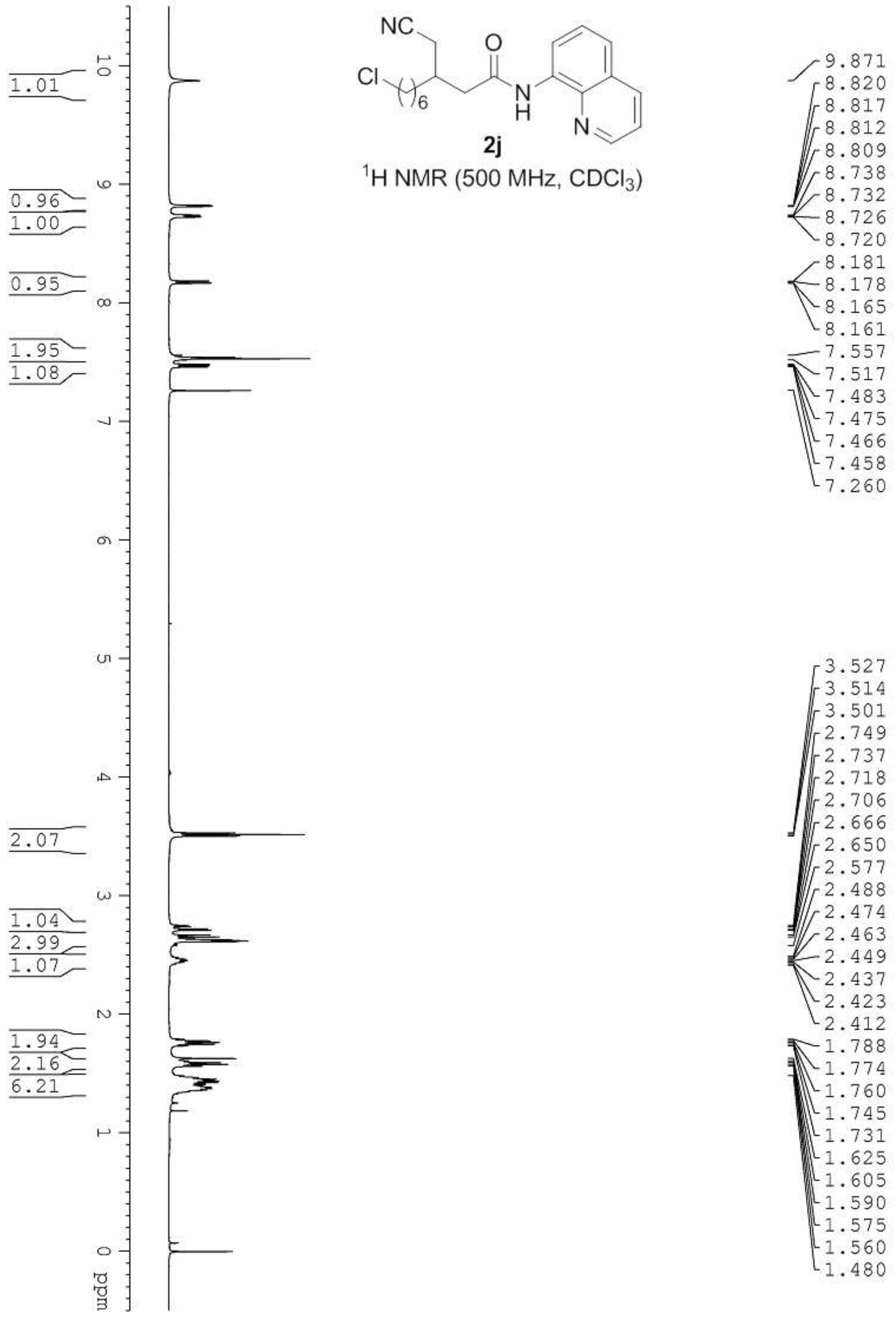

2j

^{1}H NMR (500 MHz, $CDCl_3$)

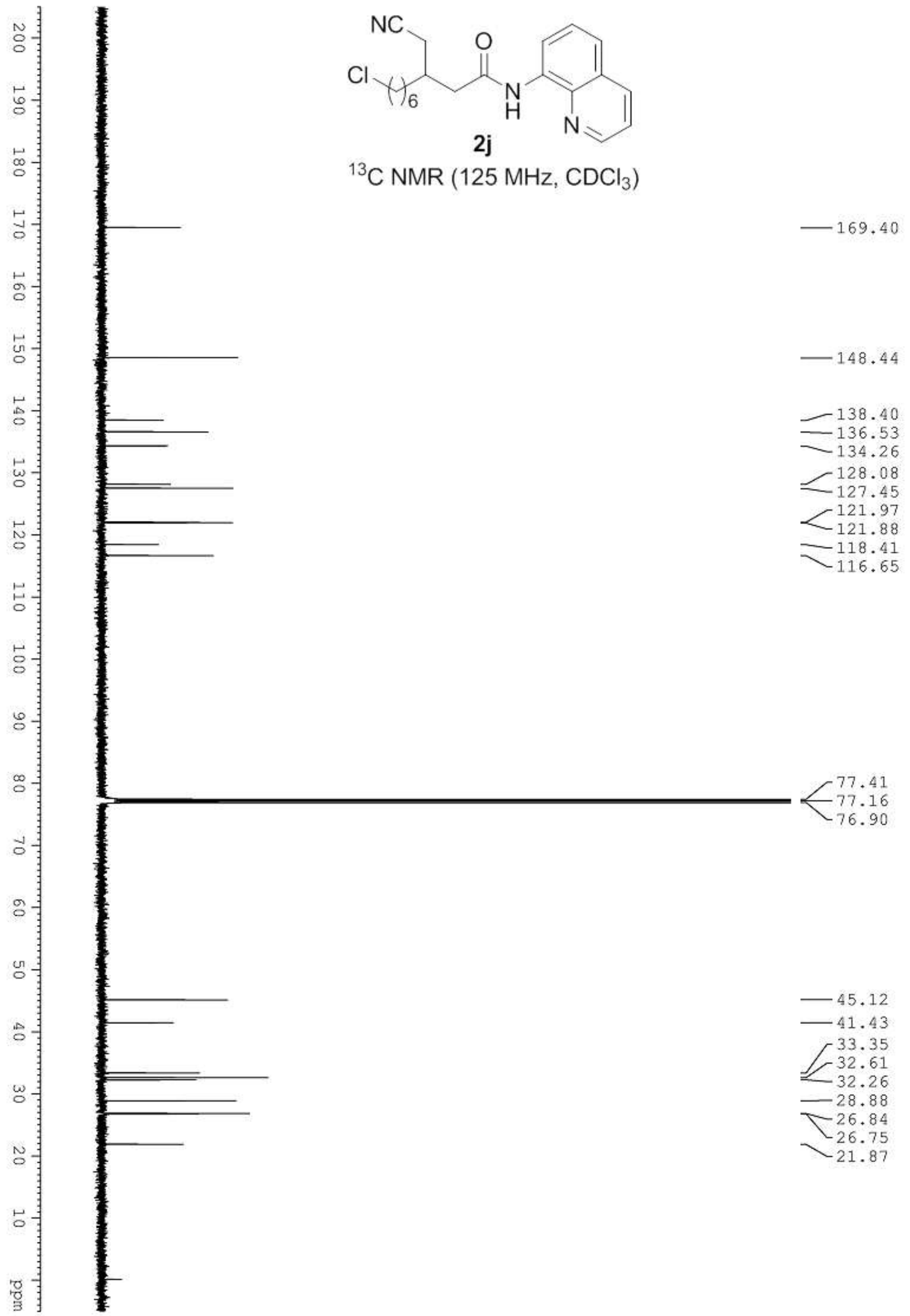

2j

^{13}C NMR (125 MHz, $CDCl_3$)

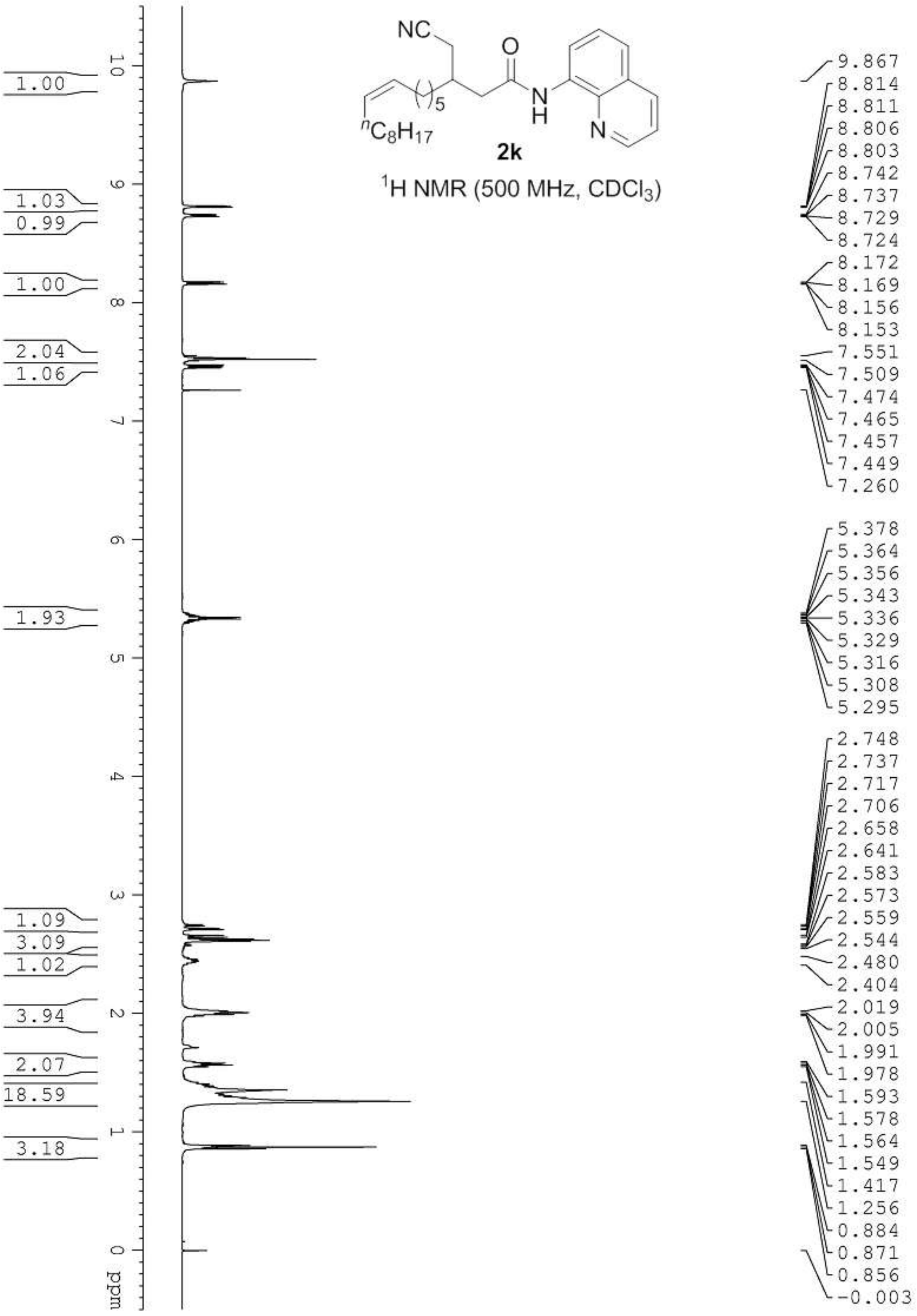
NC
O
N
H
N
5
$^nC_8H_{17}$
2k
^{1}H NMR (500 MHz, $CDCl_3$)
1.00
1.03
0.99
1.00
2.04
1.06
1.93
1.09
3.09
1.02
3.94
2.07
18.59
3.18
10
9
8
7
6
5
4
3
2
1
0 ppm
9.867
8.814
8.811
8.806
8.803
8.742
8.737
8.729
8.724
8.172
8.169
8.156
8.153
7.551
7.509
7.474
7.465
7.457
7.449
7.260
5.378
5.364
5.356
5.343
5.336
5.329
5.316
5.308
5.295
2.748
2.737
2.717
2.706
2.658
2.641
2.583
2.573
2.559
2.544
2.480
2.404
2.019
2.005
1.991
1.978
1.593
1.578
1.564
1.549
1.417
1.256
0.884
0.871
0.856
-0.003

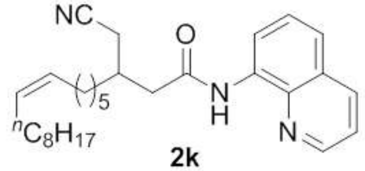

2k

^{13}C NMR (125 MHz, $CDCl_3$)

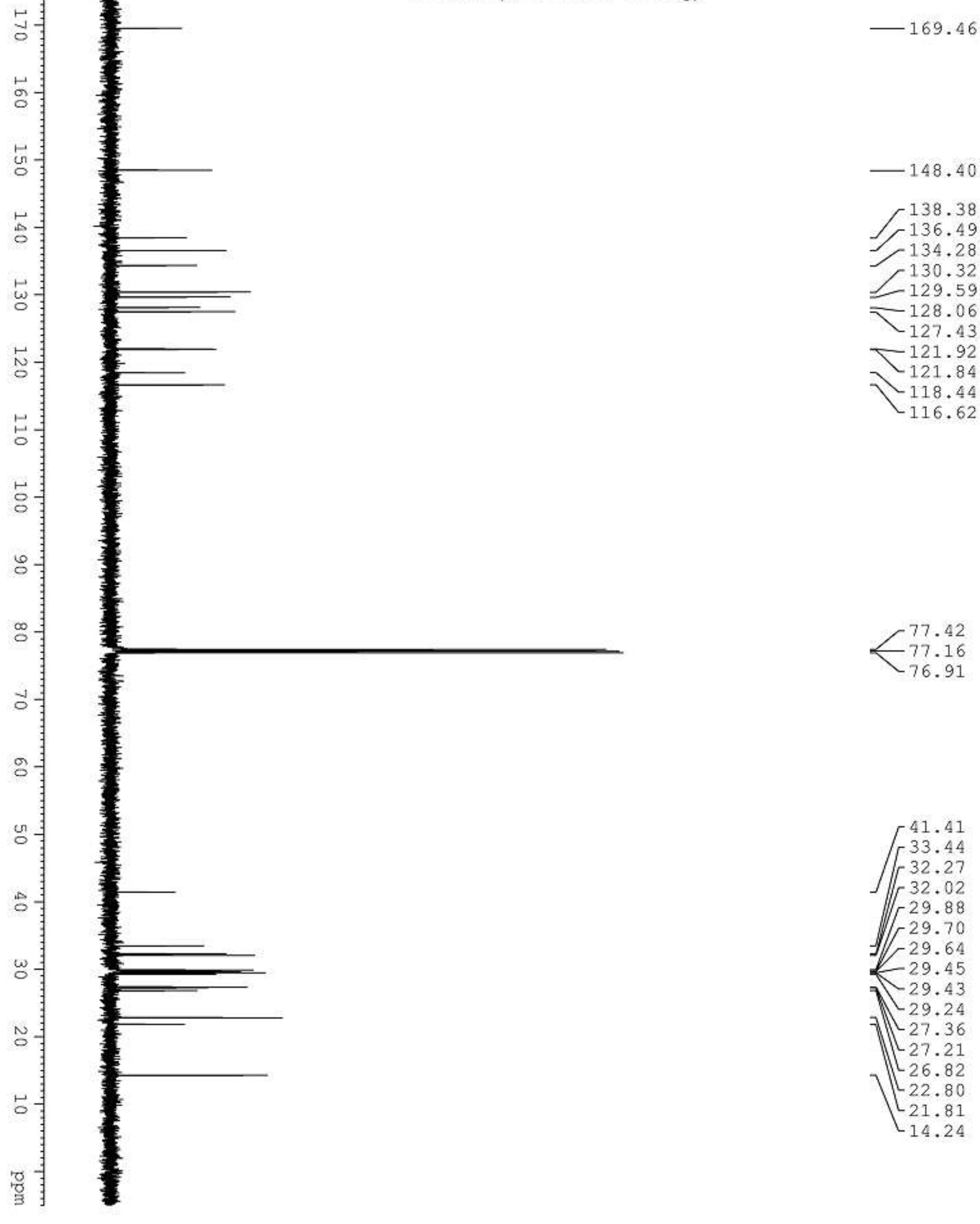

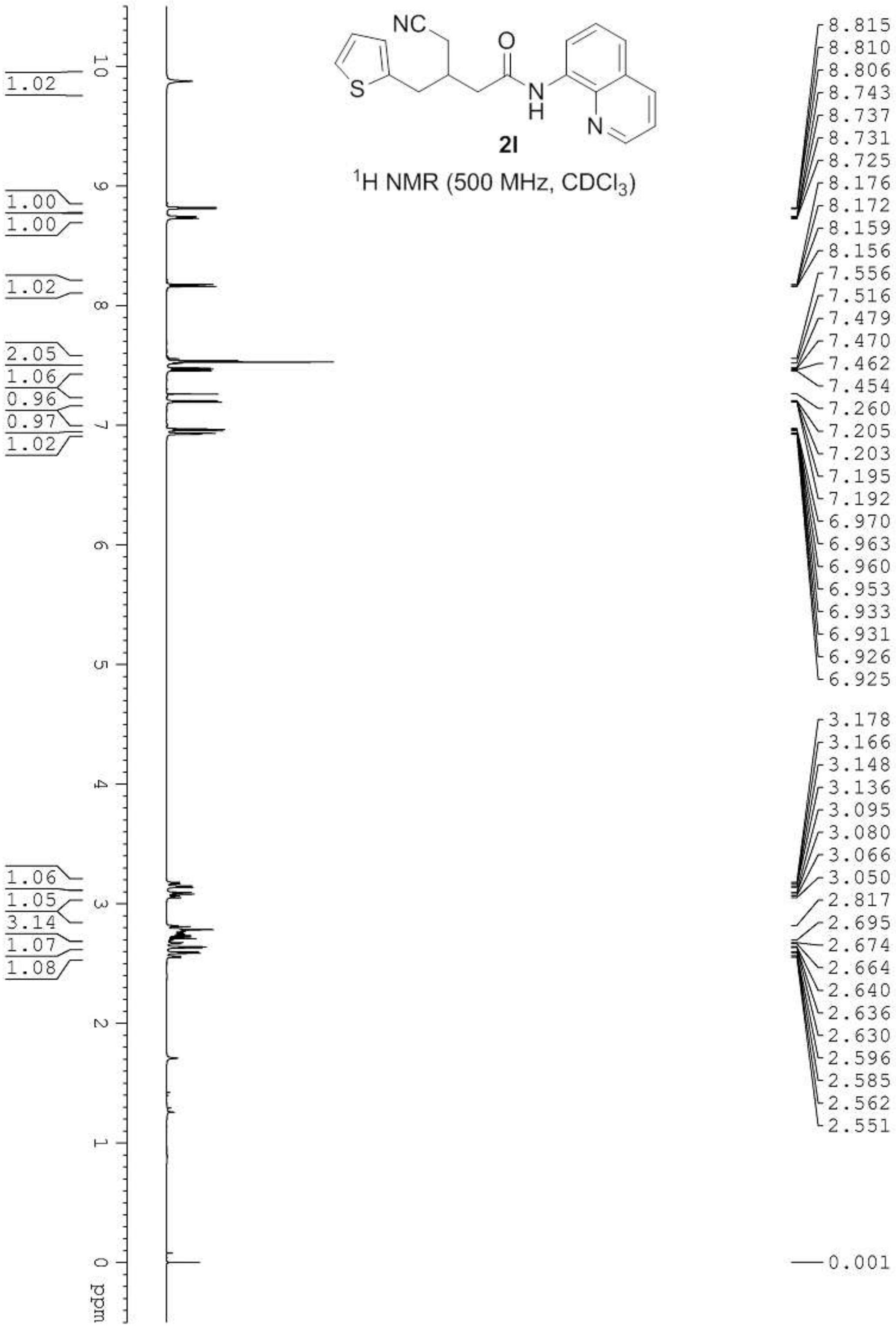

2l

^{1}H NMR (500 MHz, $CDCl_3$)

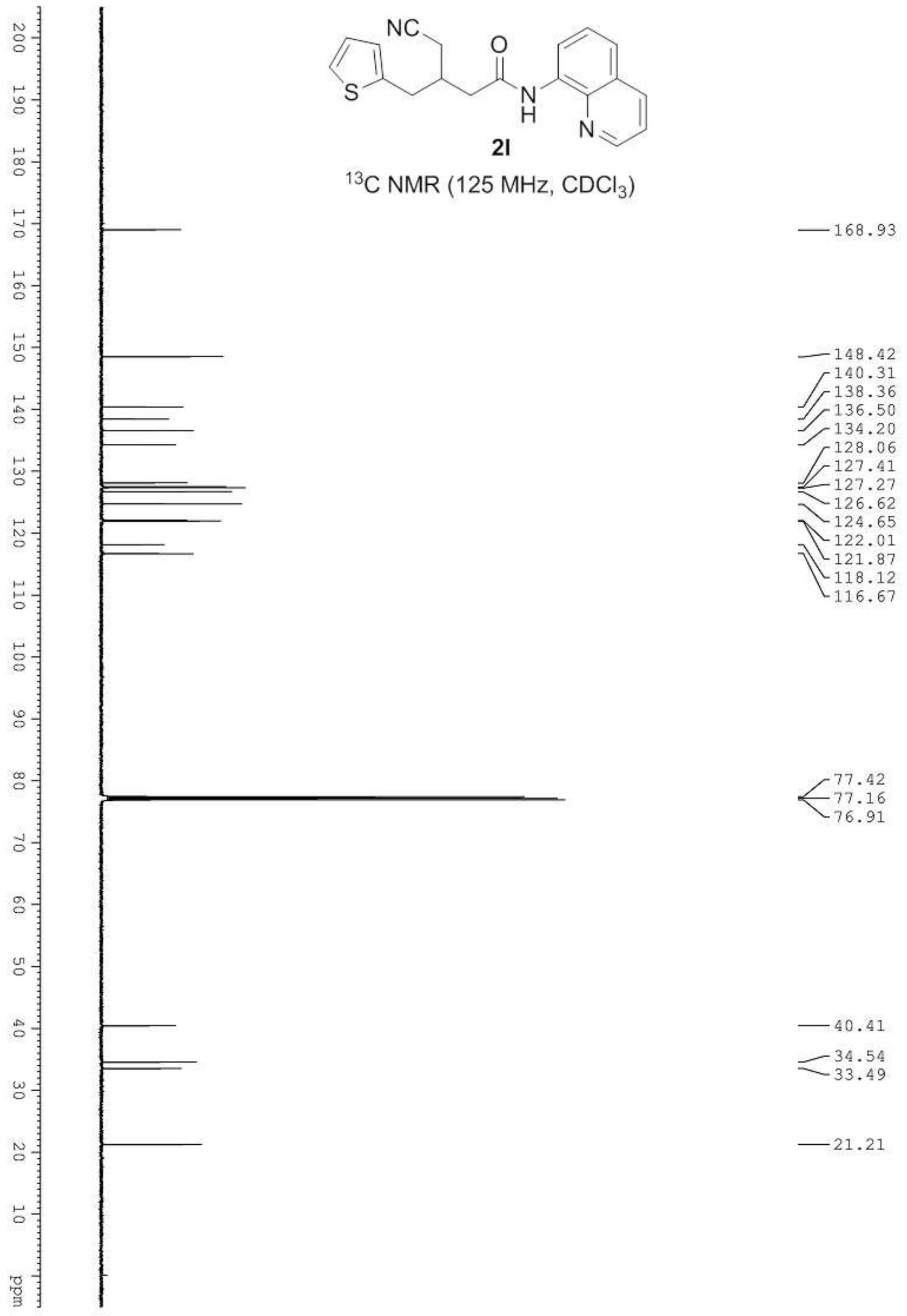

NC
O
S
N
H
N
2l
13C NMR (125 MHz, CDCl3)
168.93
148.42
140.31
138.36
136.50
134.20
128.06
127.41
127.27
126.62
124.65
122.01
121.87
118.12
116.67
77.42
77.16
76.91
40.41
34.54
33.49
21.21
ppm

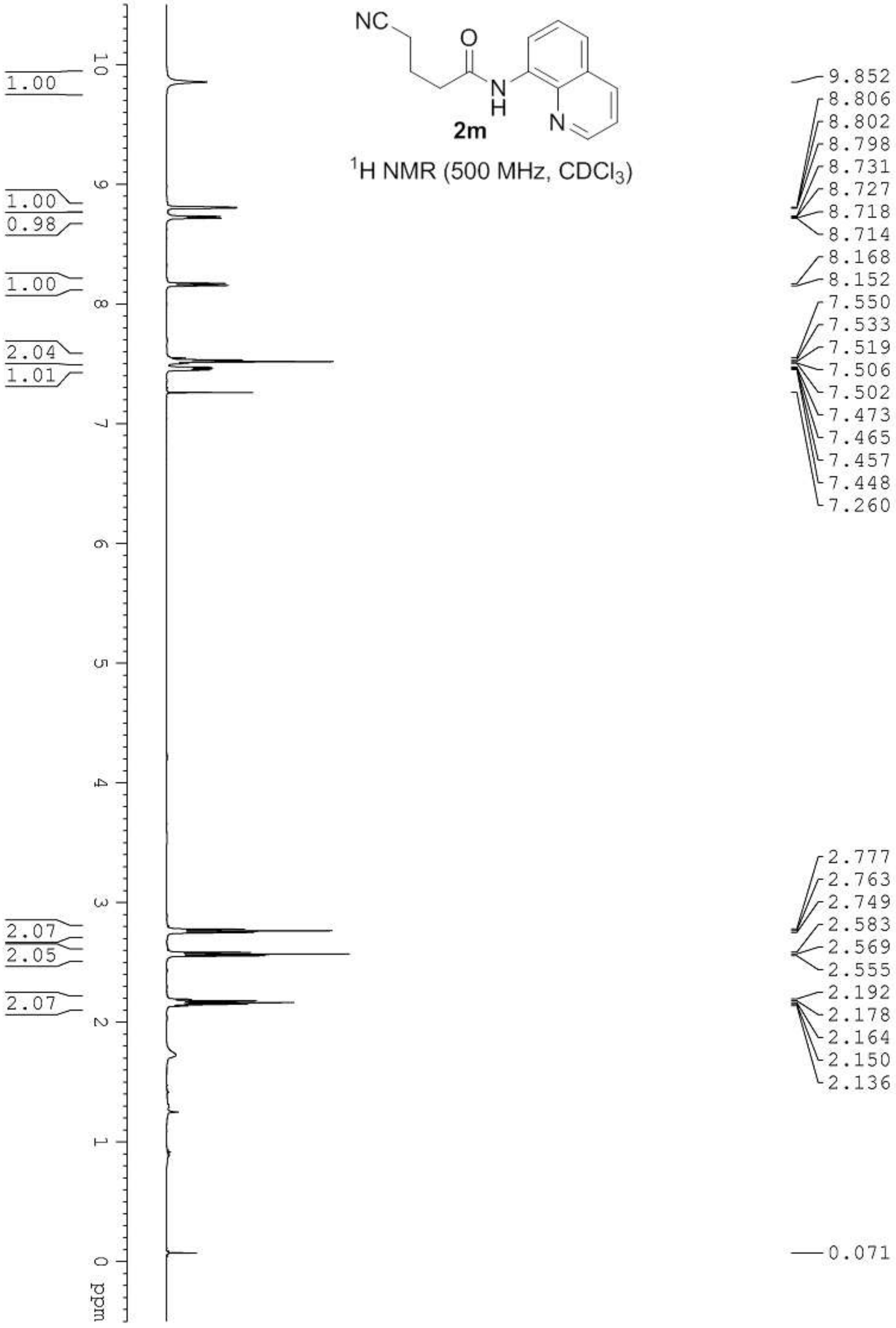

2m

^{1}H NMR (500 MHz, $CDCl_3$)

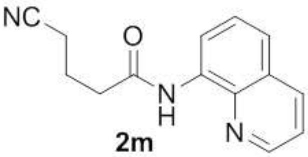

2m

^{13}C NMR (125 MHz, $CDCl_3$)

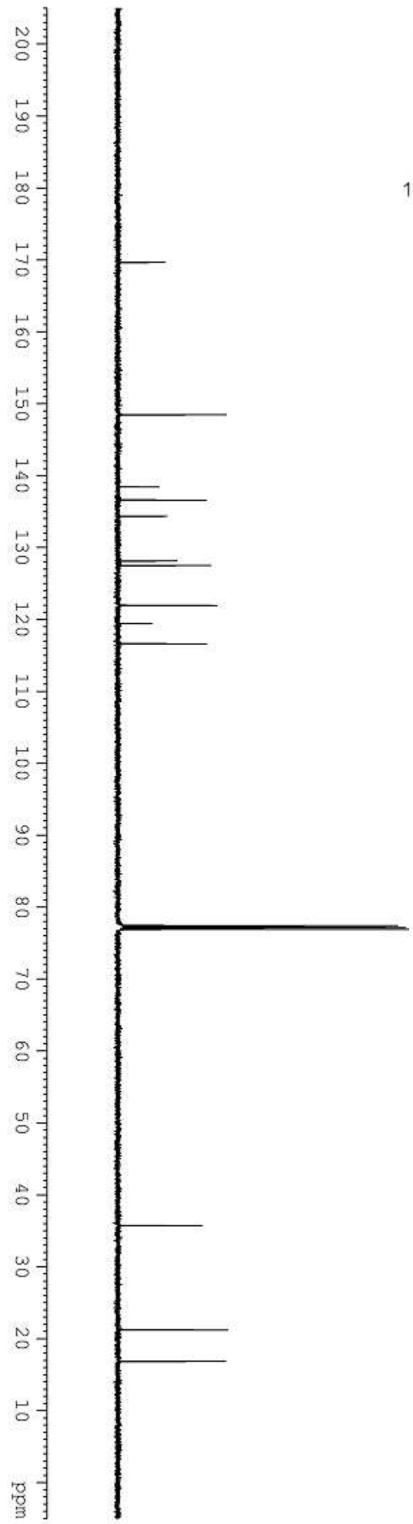

4a

^1H NMR (500 MHz, $CDCl_3$)

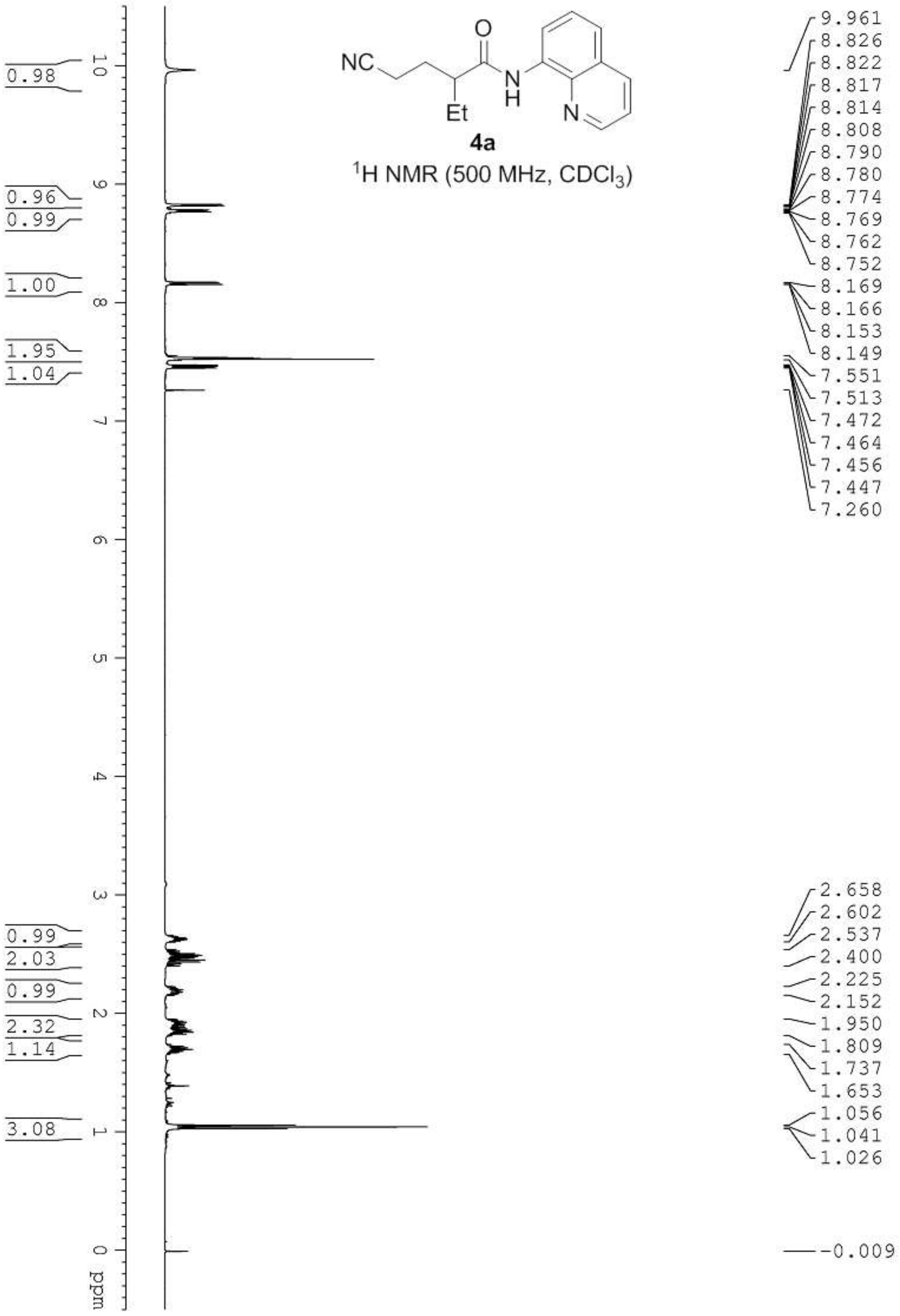

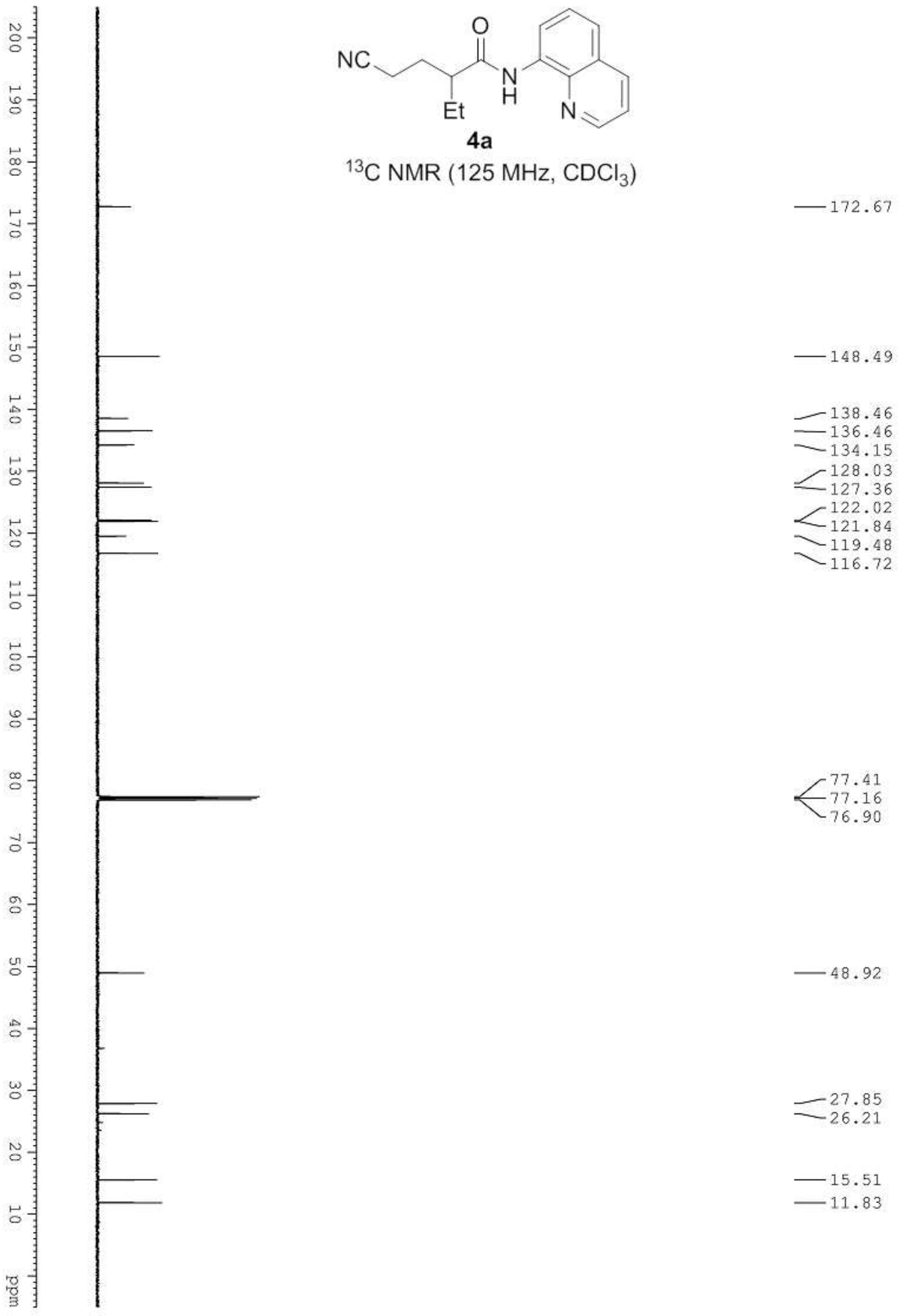

4a

^{13}C NMR (125 MHz, $CDCl_3$)

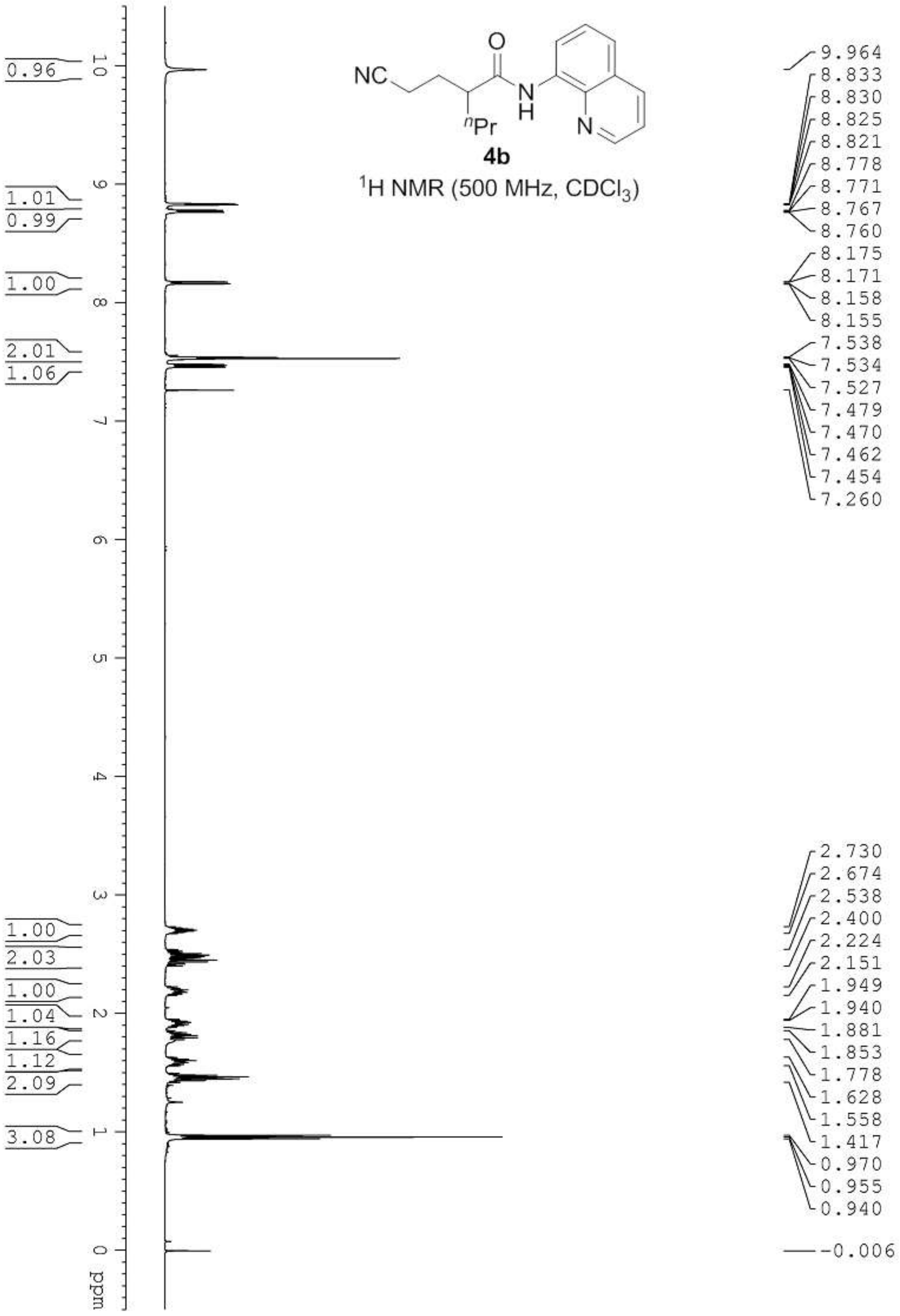
NC
O
N
H
N
nPr
4b
1H NMR (500 MHz, CDCl3)
9.964
8.833
8.830
8.825
8.821
8.778
8.771
8.767
8.760
8.175
8.171
8.158
8.155
7.538
7.534
7.527
7.479
7.470
7.462
7.454
7.260
2.730
2.674
2.538
2.400
2.224
2.151
1.949
1.940
1.881
1.853
1.778
1.628
1.558
1.417
0.970
0.955
0.940
-0.006
0.96
1.01
0.99
1.00
2.01
1.06
1.00
2.03
1.00
1.04
1.16
1.12
2.09
3.08
ppm

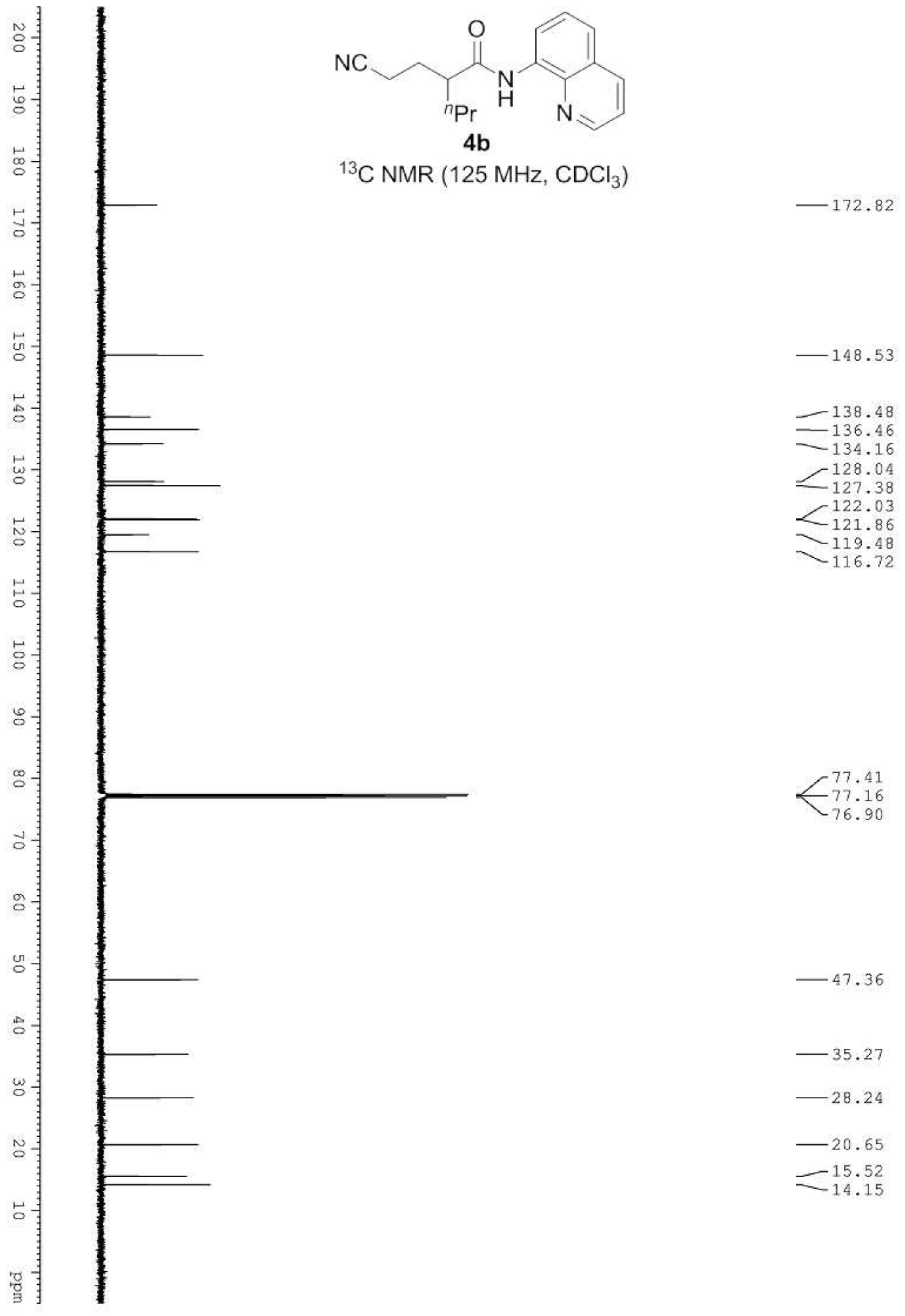

4b

^{13}C NMR (125 MHz, $CDCl_3$)

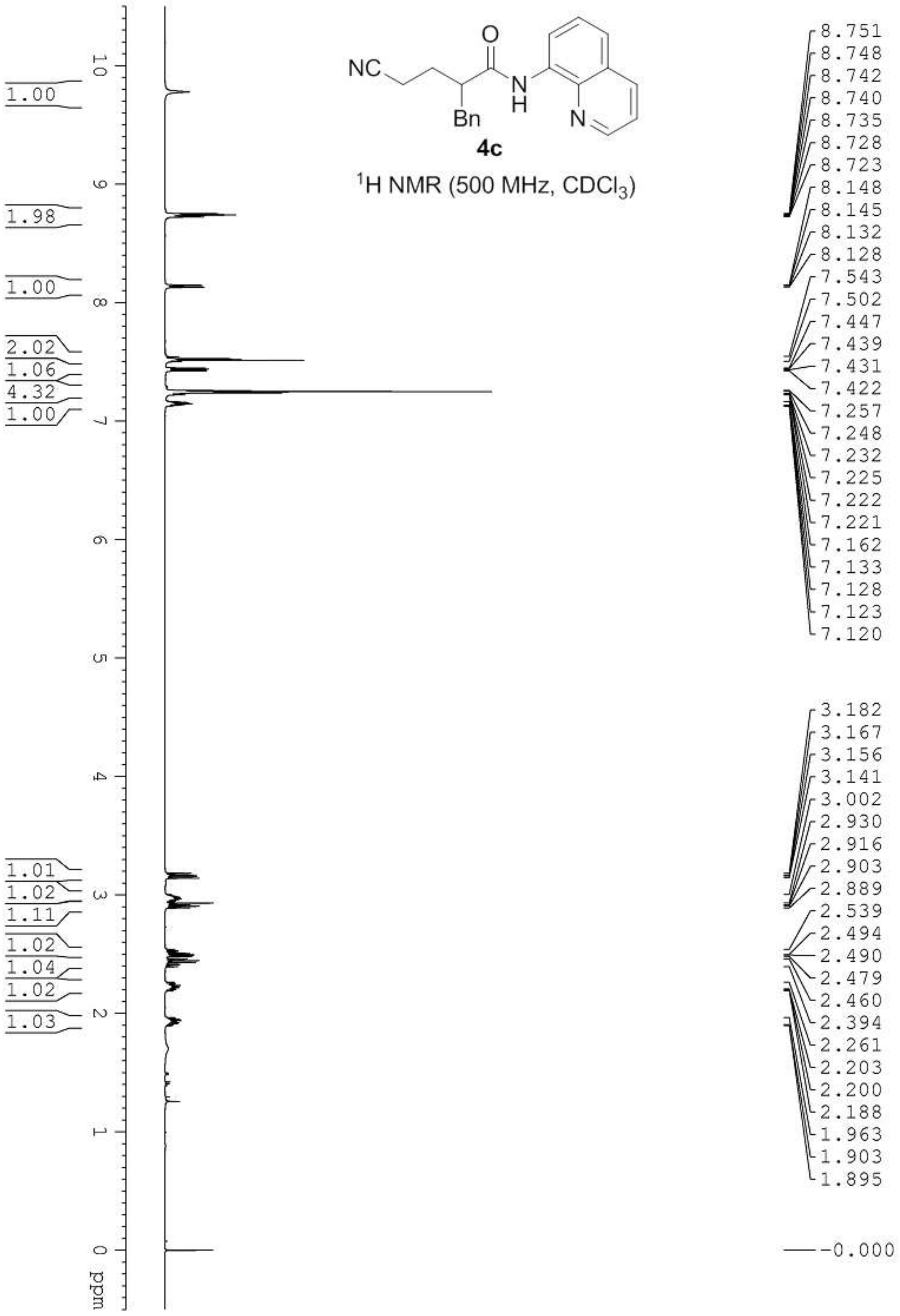

NC
O
N
H
Bn
N
4c
1H NMR (500 MHz, CDCl3)
8.751
8.748
8.742
8.740
8.735
8.728
8.723
8.148
8.145
8.132
8.128
7.543
7.502
7.447
7.439
7.431
7.422
7.257
7.248
7.232
7.225
7.222
7.221
7.162
7.133
7.128
7.123
7.120
3.182
3.167
3.156
3.141
3.002
2.930
2.916
2.903
2.889
2.539
2.494
2.490
2.479
2.460
2.394
2.261
2.203
2.200
2.188
1.963
1.903
1.895
-0.000
1.00
1.98
1.00
2.02
1.06
4.32
1.00
1.01
1.02
1.11
1.02
1.04
1.02
1.03
ppm

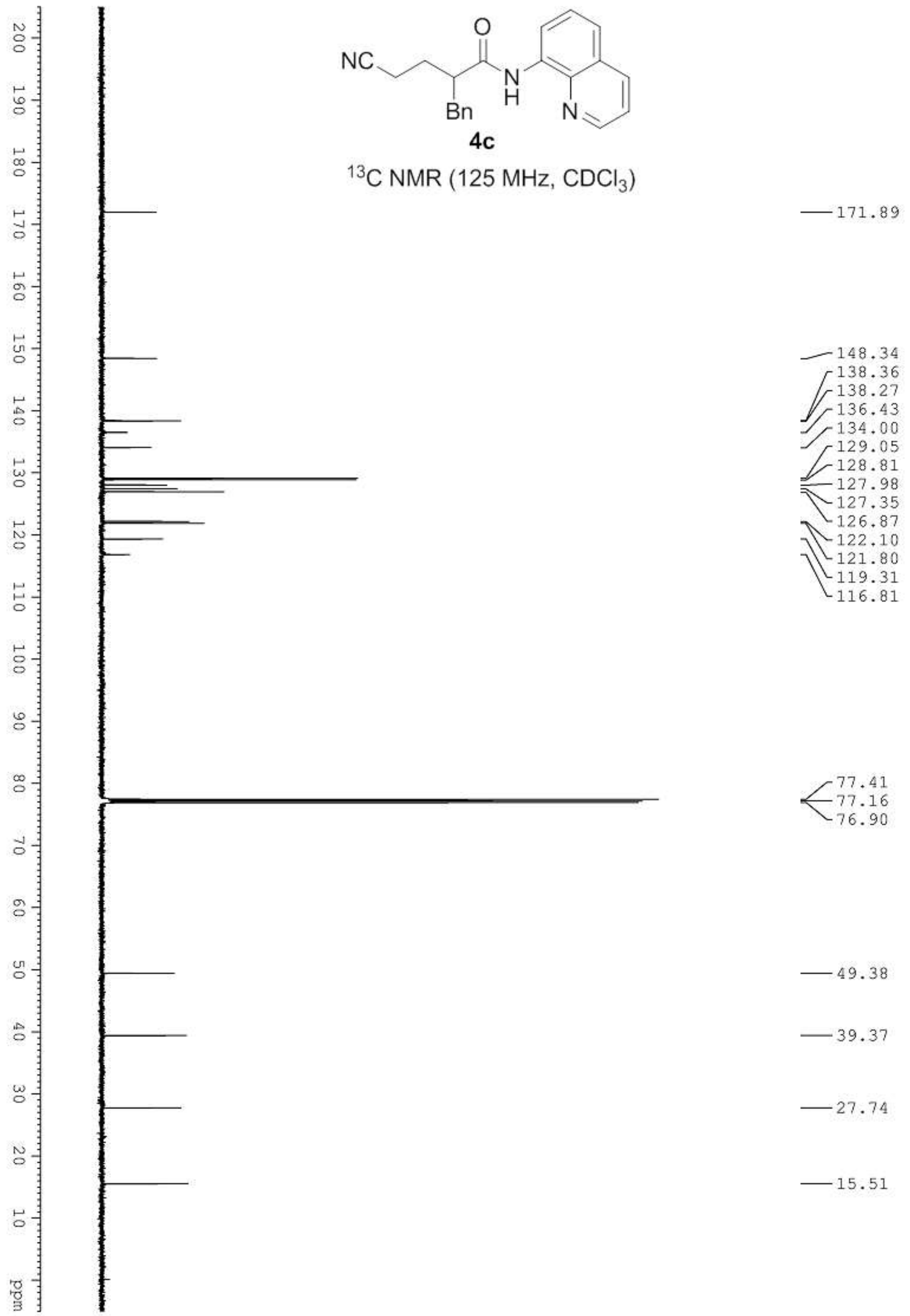

4c

^{13}C NMR (125 MHz, $CDCl_3$)

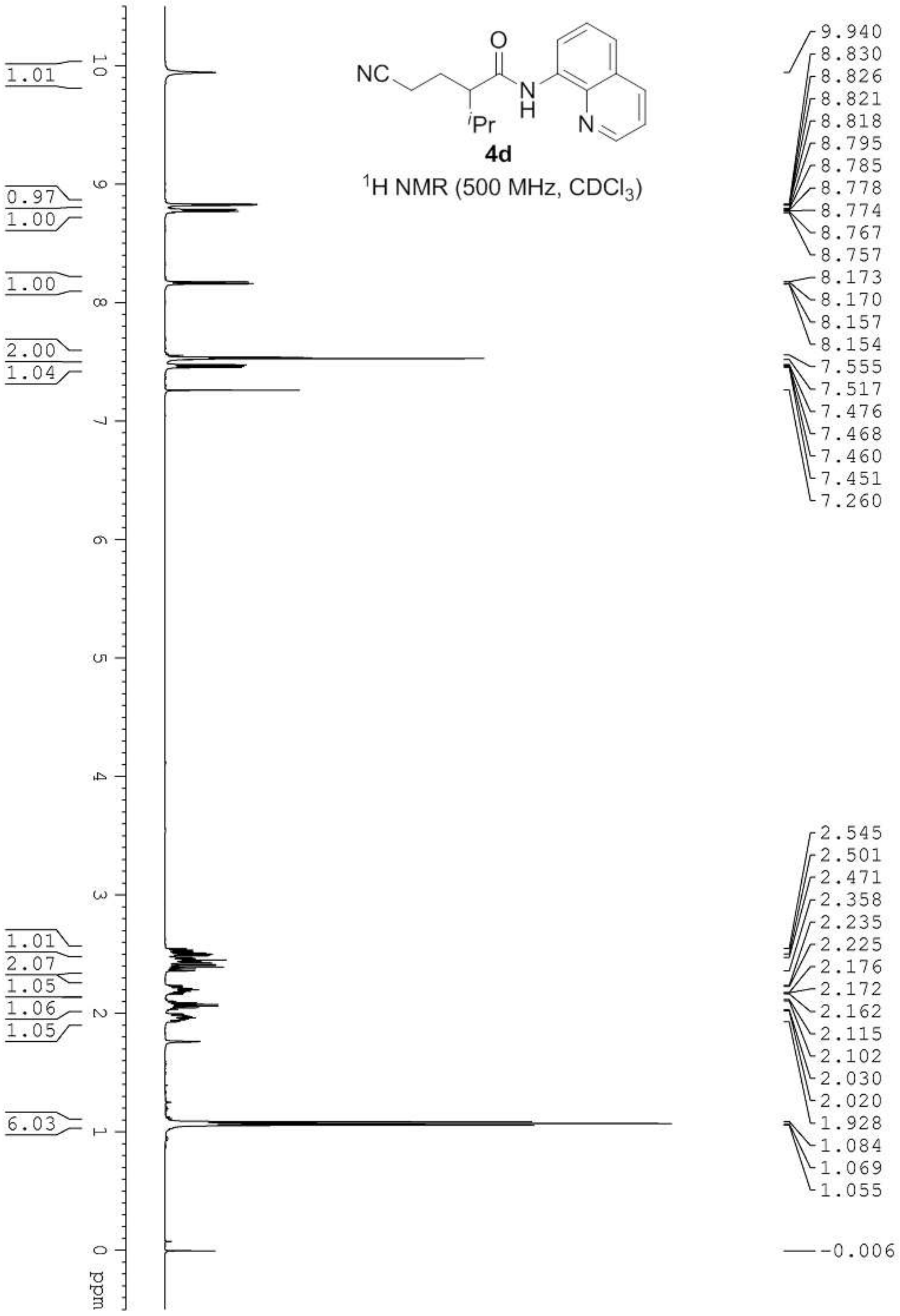
4d
1H NMR (500 MHz, CDCl3)
9.940
8.830
8.826
8.821
8.818
8.795
8.785
8.778
8.774
8.767
8.757
8.173
8.170
8.157
8.154
7.555
7.517
7.476
7.468
7.460
7.451
7.260
2.545
2.501
2.471
2.358
2.235
2.225
2.176
2.172
2.162
2.115
2.102
2.030
2.020
1.928
1.084
1.069
1.055
-0.006
1.01
0.97
1.00
1.00
2.00
1.04
1.01
2.07
1.05
1.06
1.05
6.03
ppm

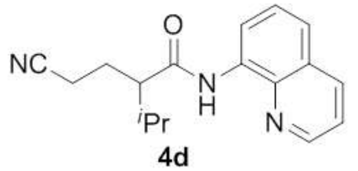

4d

^{13}C NMR (125 MHz, $CDCl_3$)

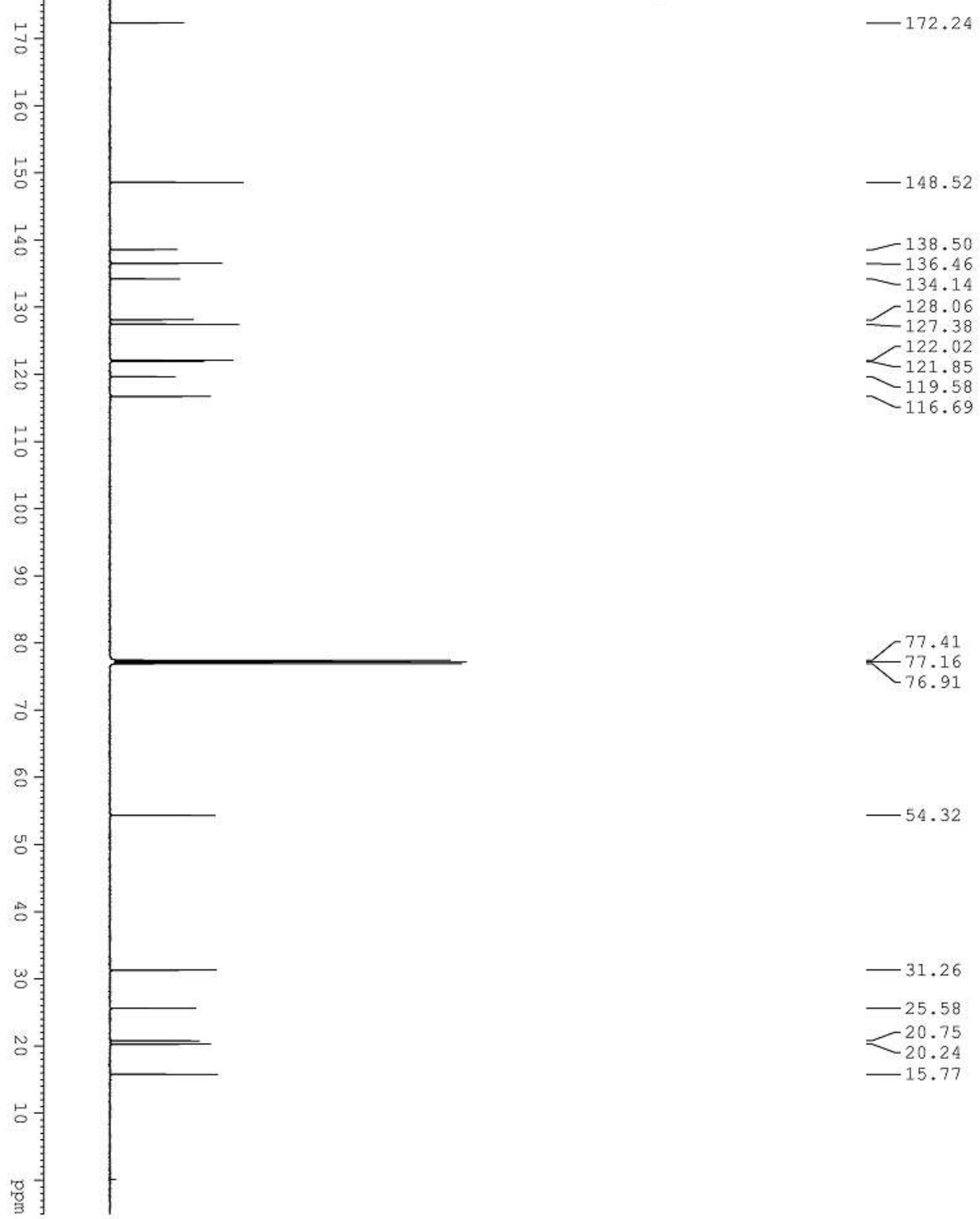

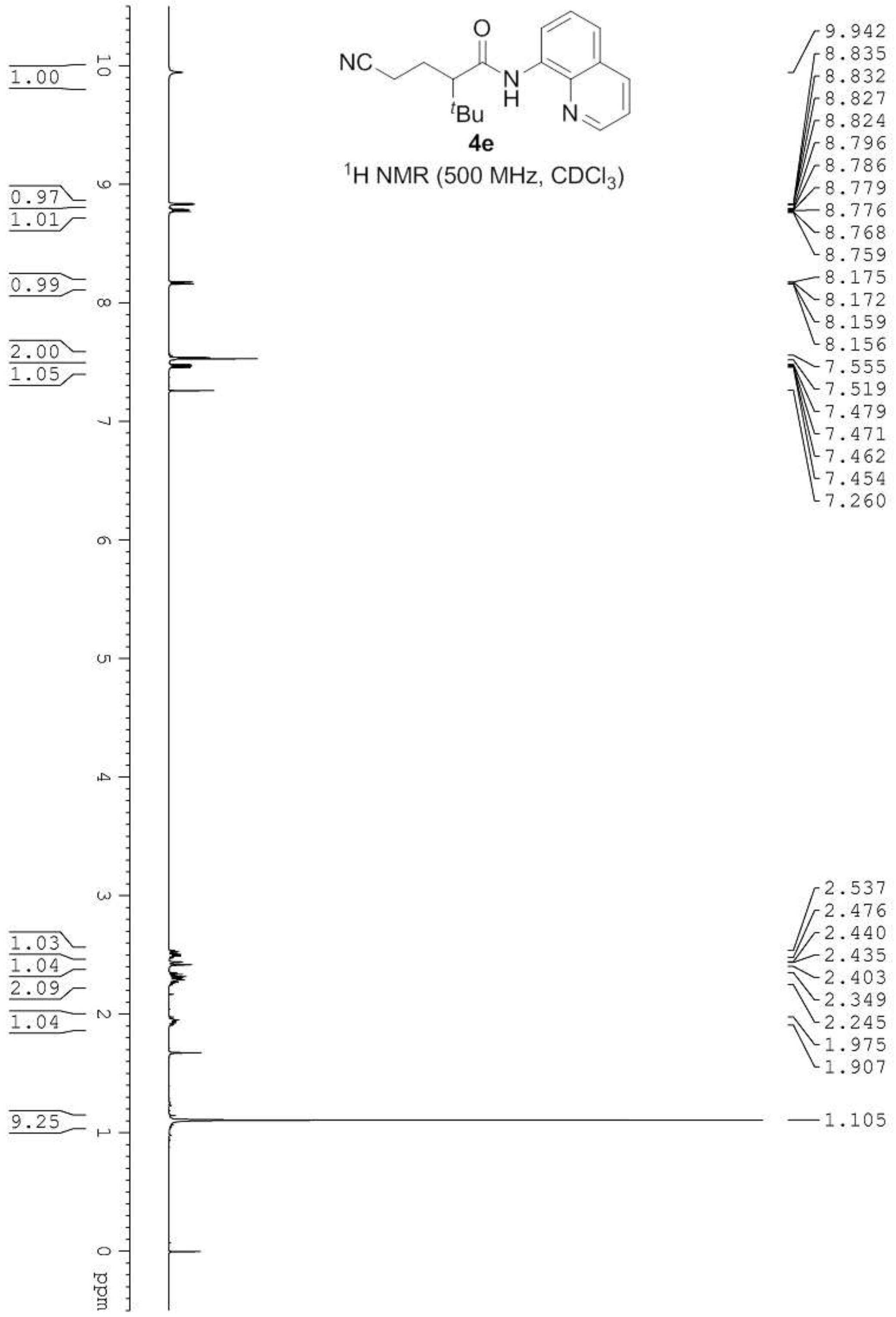
NC
O
N
H
N
tBu
4e
1H NMR (500 MHz, CDCl3)
9.942
8.835
8.832
8.827
8.824
8.796
8.786
8.779
8.776
8.768
8.759
8.175
8.172
8.159
8.156
7.555
7.519
7.479
7.471
7.462
7.454
7.260
2.537
2.476
2.440
2.435
2.403
2.349
2.245
1.975
1.907
1.105
1.00
0.97
1.01
0.99
2.00
1.05
1.03
1.04
2.09
1.04
9.25
10
9
8
7
6
5
4
3
2
1
0 ppm

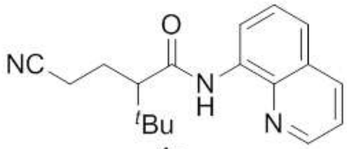

4e

^{13}C NMR (125 MHz, $CDCl_3$)

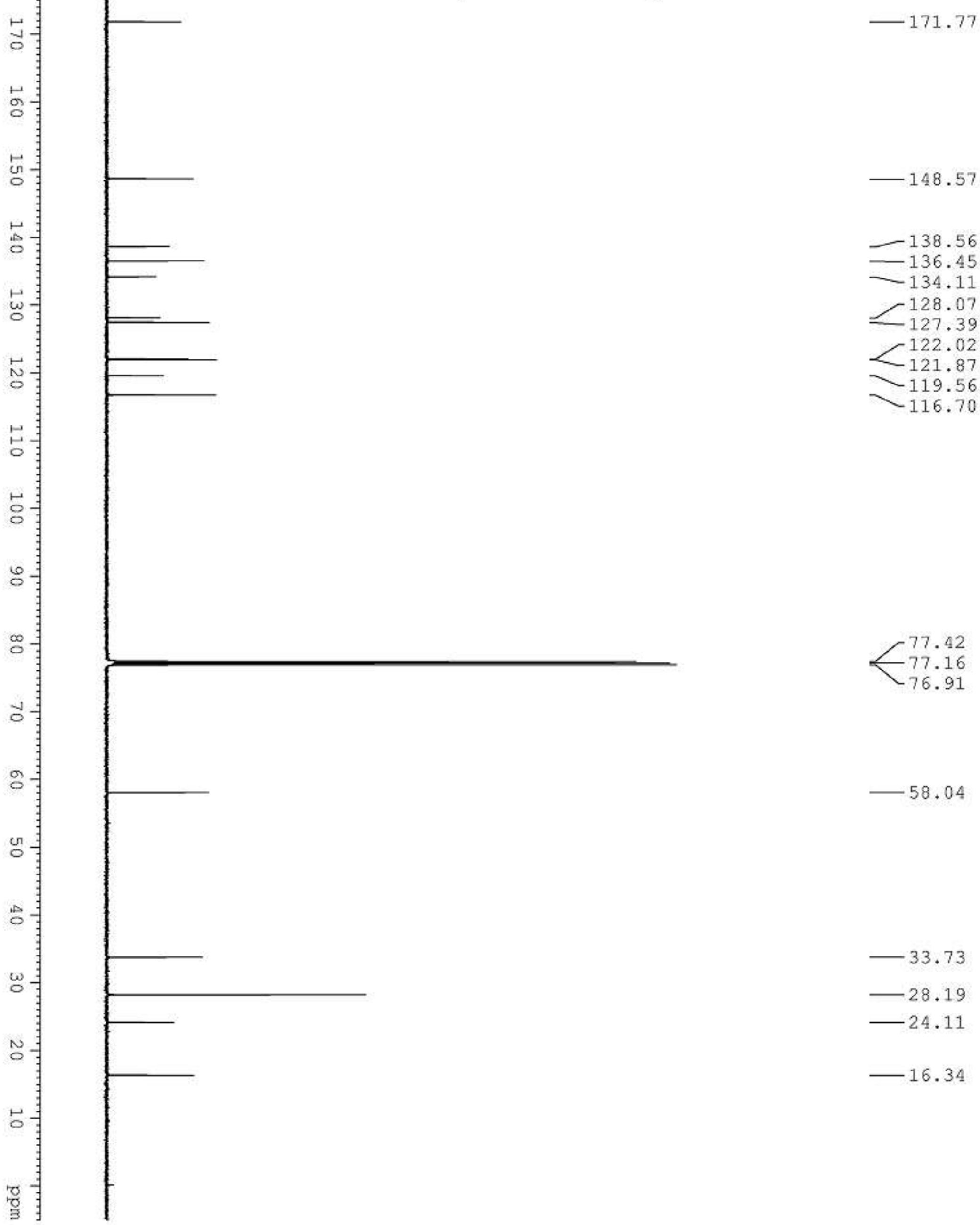

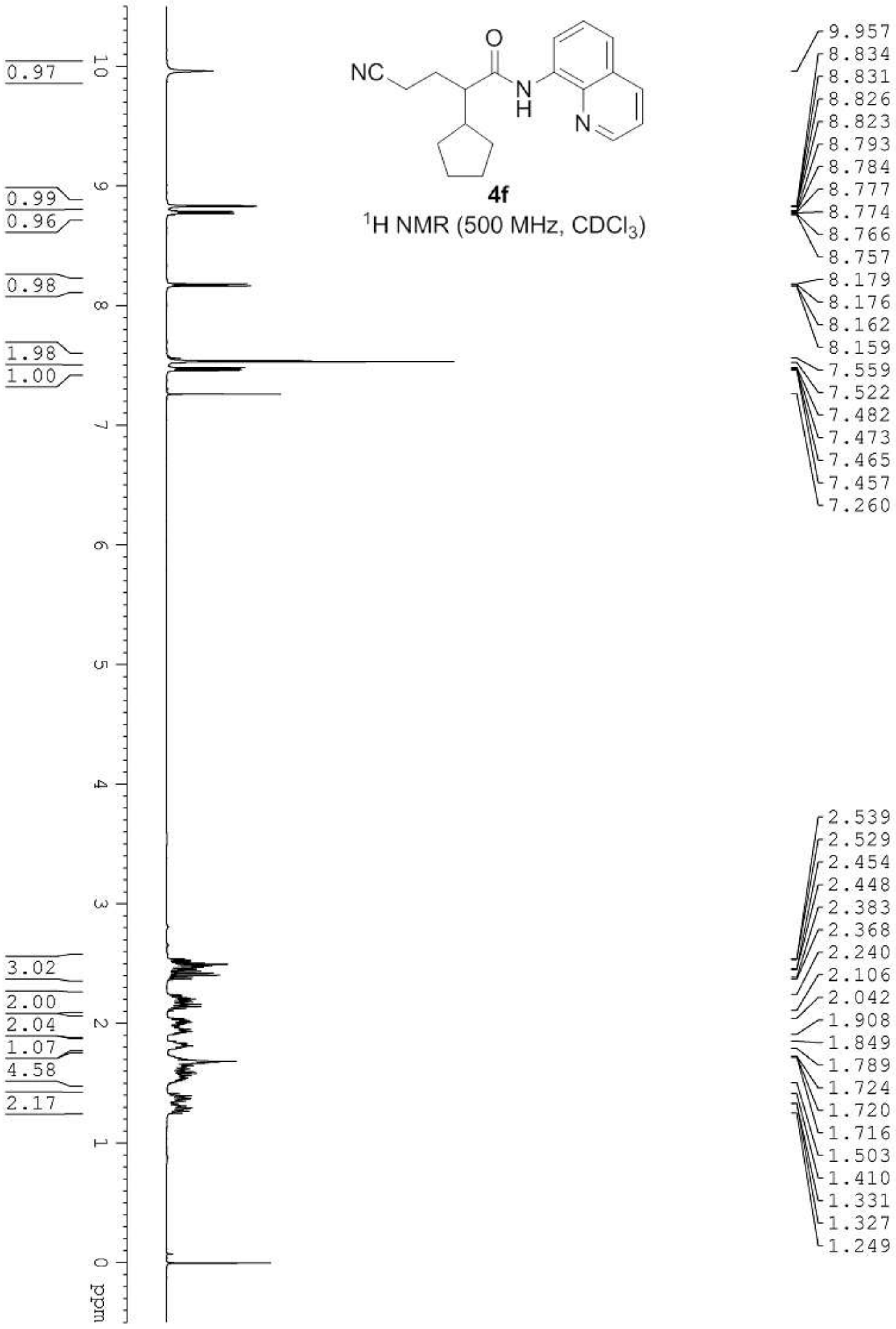
NC
O
N
H
N
4f
1H NMR (500 MHz, CDCl3)
9.957
8.834
8.831
8.826
8.823
8.793
8.784
8.777
8.774
8.766
8.757
8.179
8.176
8.162
8.159
7.559
7.522
7.482
7.473
7.465
7.457
7.260
2.539
2.529
2.454
2.448
2.383
2.368
2.240
2.106
2.042
1.908
1.849
1.789
1.724
1.720
1.716
1.503
1.410
1.331
1.327
1.249
0.97
0.99
0.96
0.98
1.98
1.00
3.02
2.00
2.04
1.07
4.58
2.17
ppm

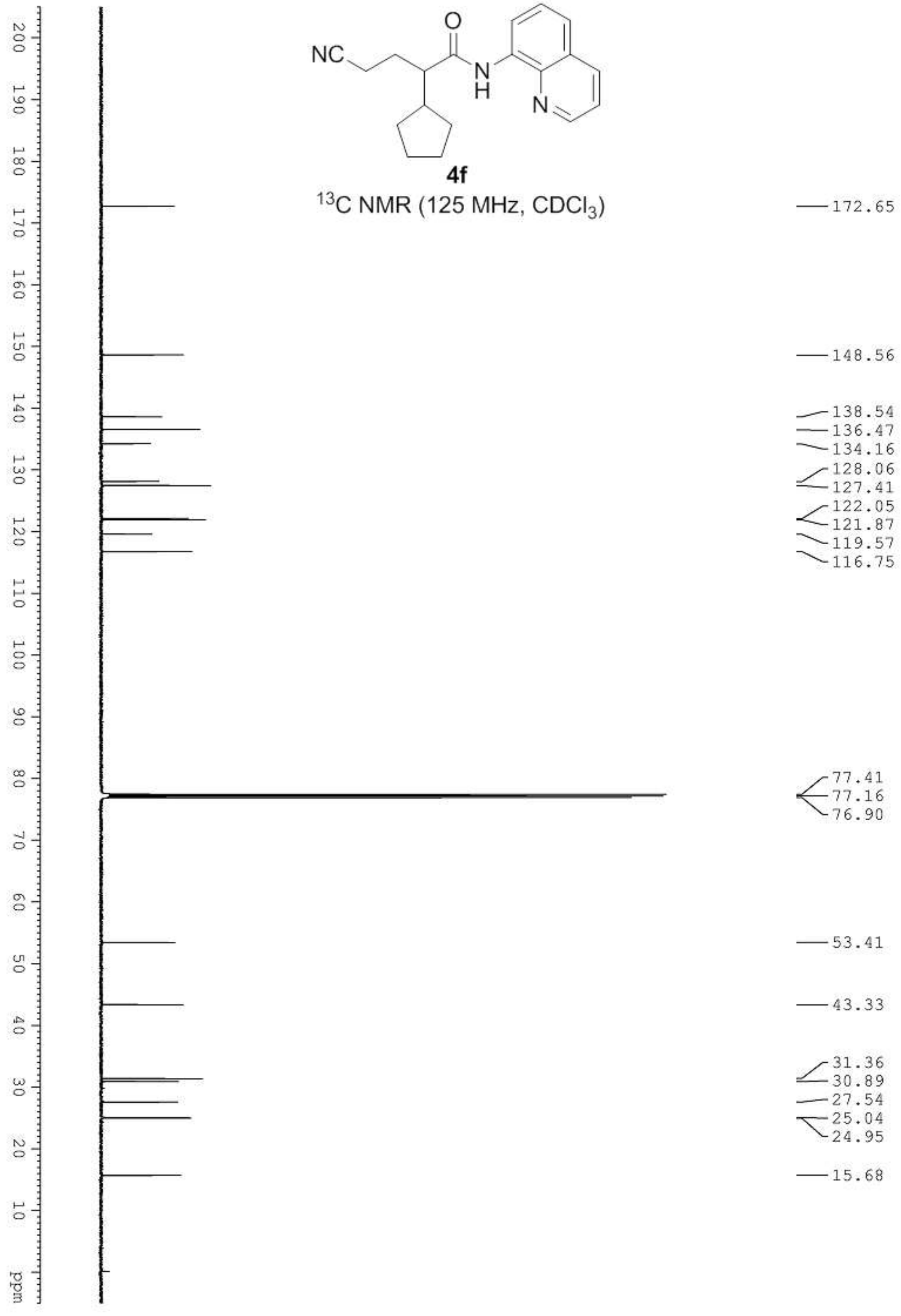
O
NC
N
H
N
4f
^{13}C NMR (125 MHz, $CDCl_3$)
172.65
148.56
138.54
136.47
134.16
128.06
127.41
122.05
121.87
119.57
116.75
77.41
77.16
76.90
53.41
43.33
31.36
30.89
27.54
25.04
24.95
15.68
200
190
180
170
160
150
140
130
120
110
100
90
80
70
60
50
40
30
20
10
ppm

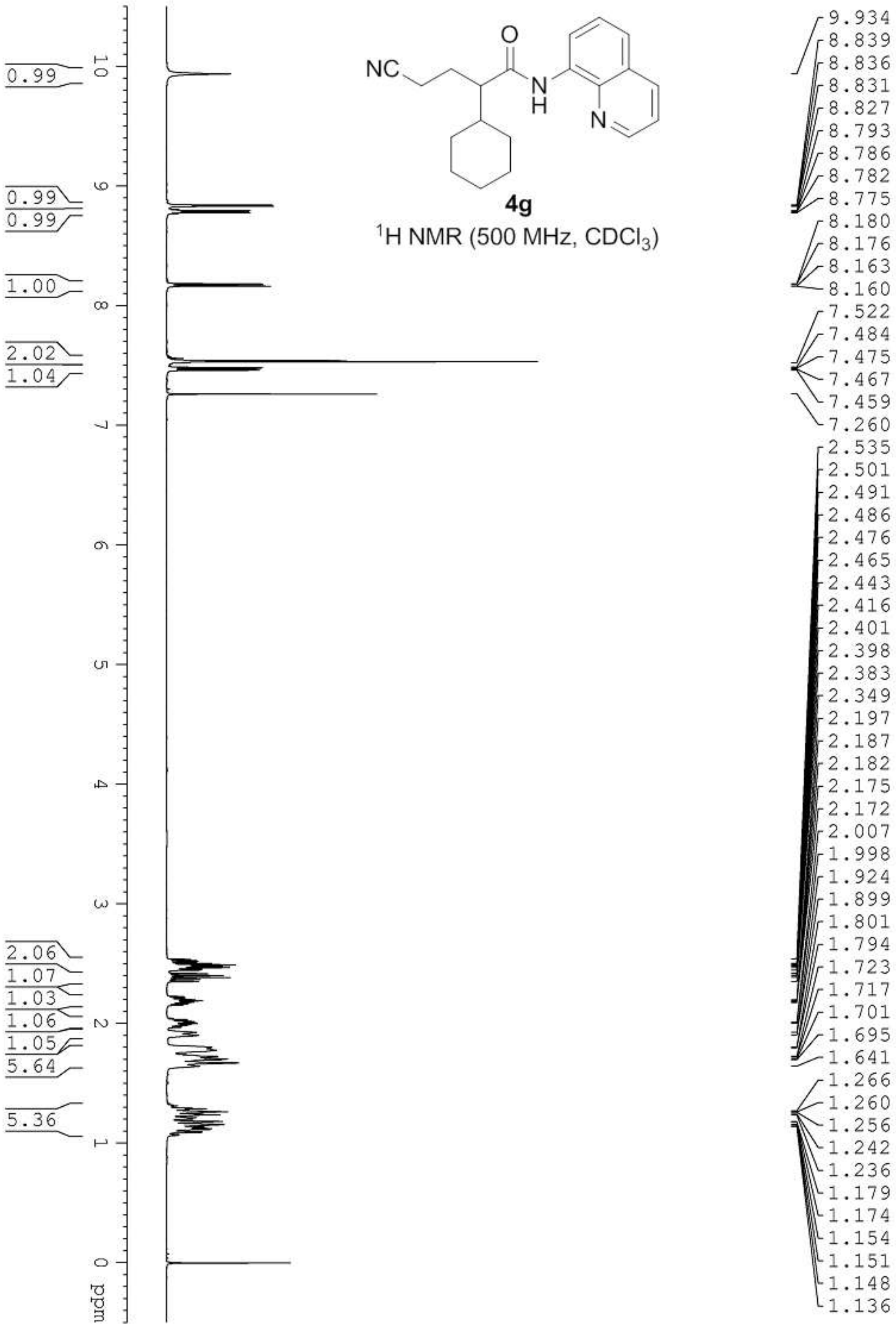
NC
O
N
H
N
4g
^{1}H NMR (500 MHz, CDCl$_3$)
9.934
8.839
8.836
8.831
8.827
8.793
8.786
8.782
8.775
8.180
8.176
8.163
8.160
7.522
7.484
7.475
7.467
7.459
7.260
2.535
2.501
2.491
2.486
2.476
2.465
2.443
2.416
2.401
2.398
2.383
2.349
2.197
2.187
2.182
2.175
2.172
2.007
1.998
1.924
1.899
1.801
1.794
1.723
1.717
1.701
1.695
1.641
1.266
1.260
1.256
1.242
1.236
1.179
1.174
1.154
1.151
1.148
1.136
0.99
0.99
0.99
1.00
2.02
1.04
2.06
1.07
1.03
1.06
1.05
5.64
5.36
ppm

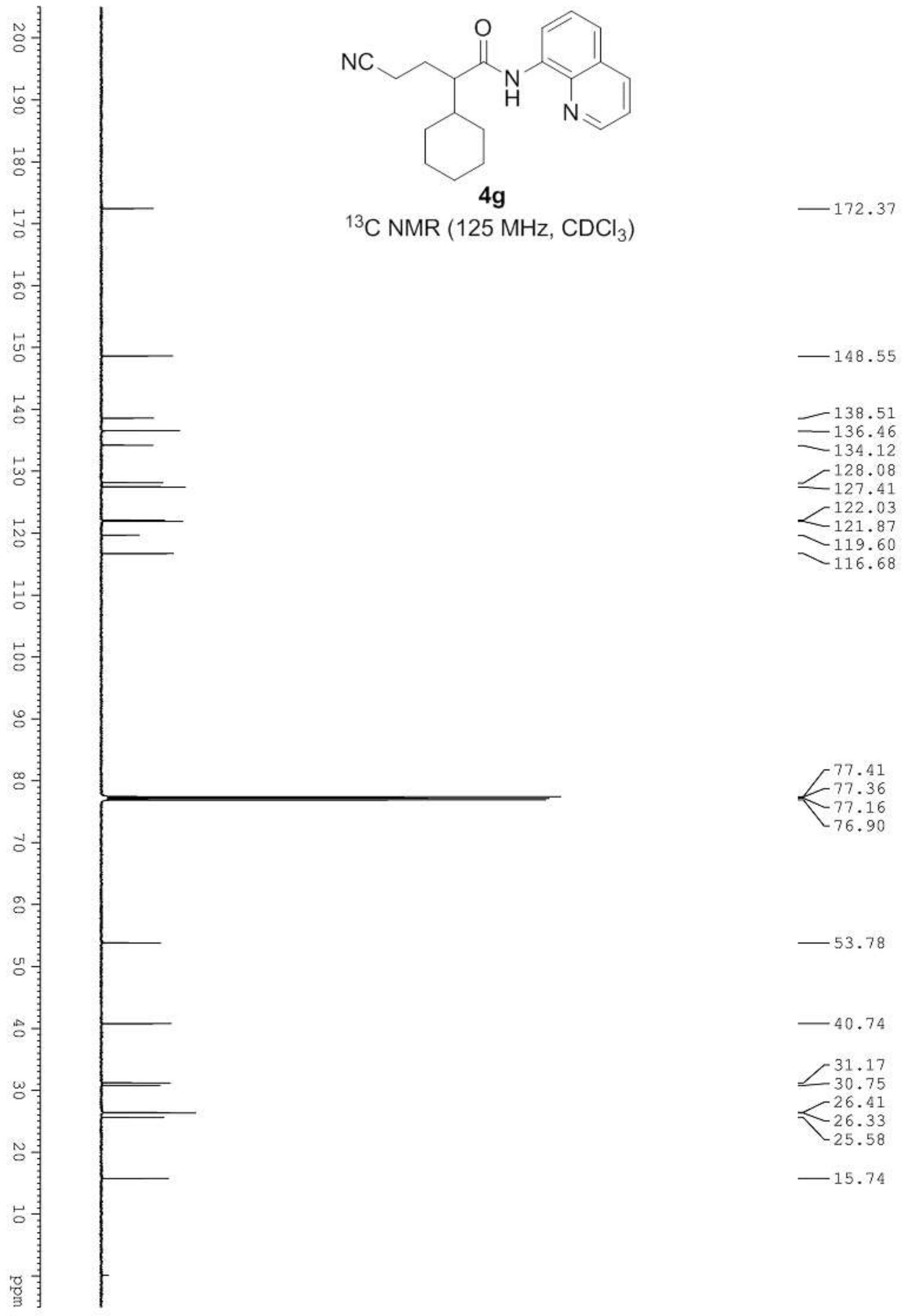

4g

^{13}C NMR (125 MHz, $CDCl_3$)

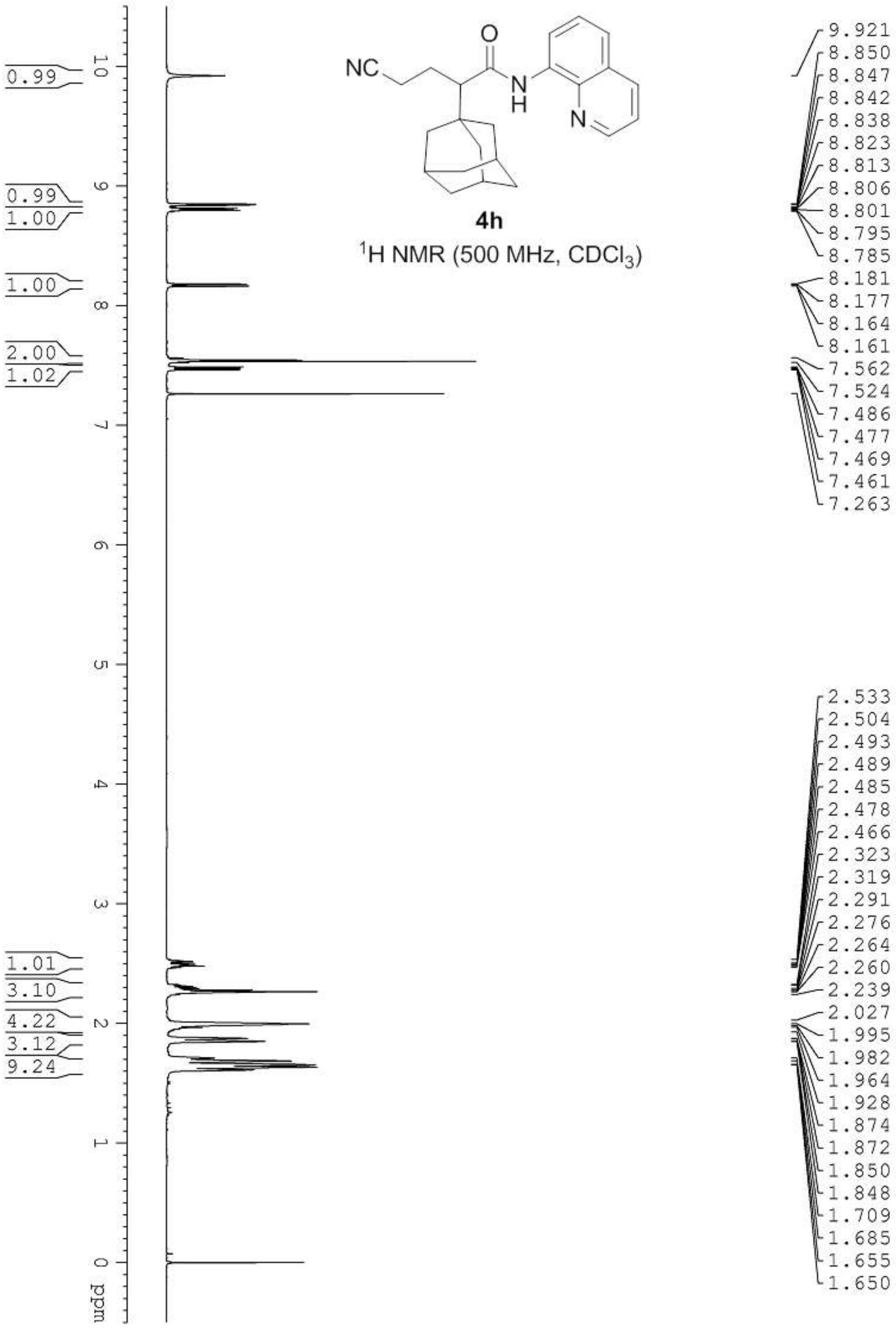

NC
O
N
H
N
4h
^{1}H NMR (500 MHz, $CDCl_3$)
9.921
8.850
8.847
8.842
8.838
8.823
8.813
8.806
8.801
8.795
8.785
8.181
8.177
8.164
8.161
7.562
7.524
7.486
7.477
7.469
7.461
7.263
2.533
2.504
2.493
2.489
2.485
2.478
2.466
2.323
2.319
2.291
2.276
2.264
2.260
2.239
2.027
1.995
1.982
1.964
1.928
1.874
1.872
1.850
1.848
1.709
1.685
1.655
1.650
0.99
0.99
1.00
1.00
2.00
1.02
1.01
3.10
4.22
3.12
9.24
10
9
8
7
6
5
4
3
2
1
0 ppm

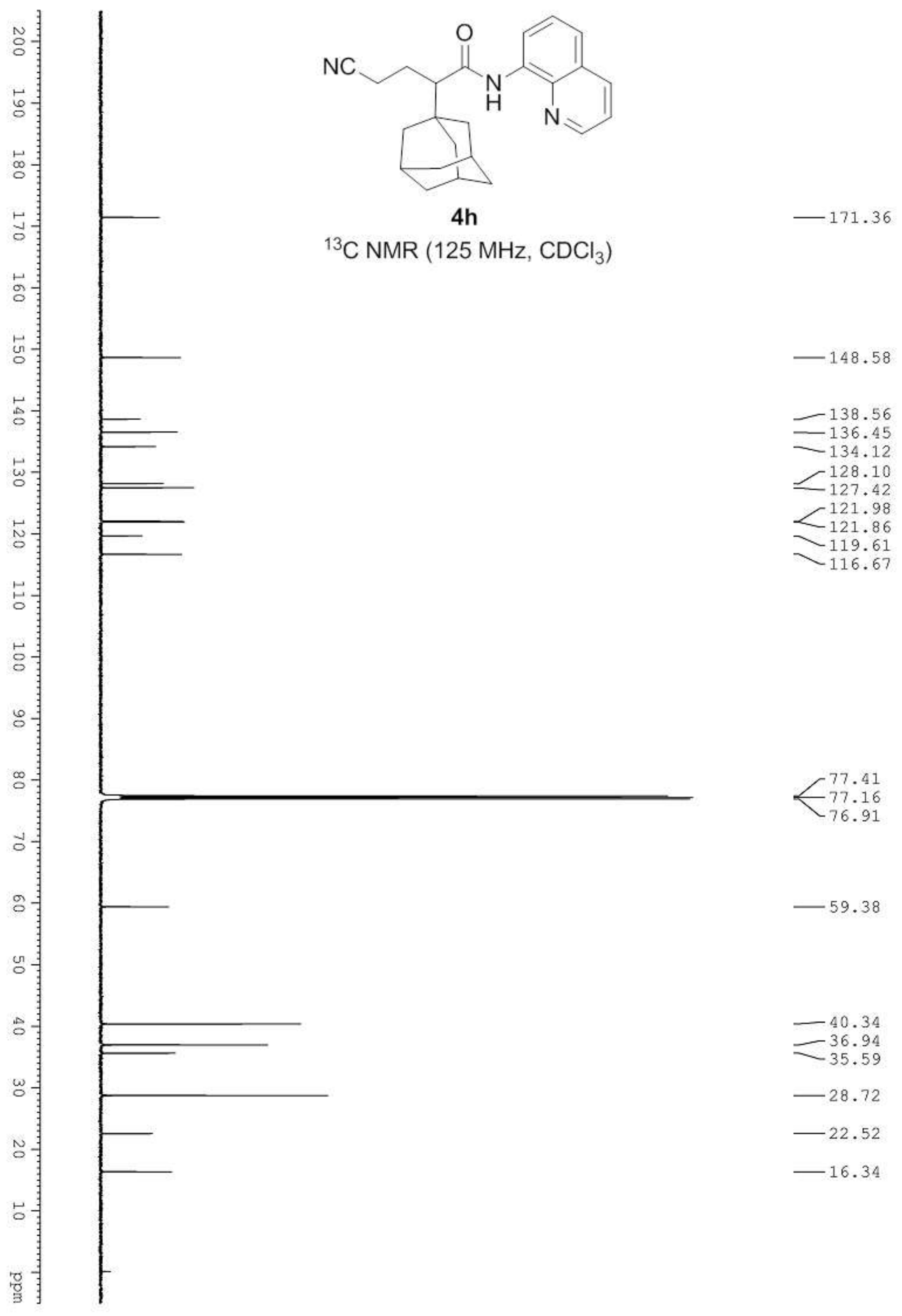

4h

^{13}C NMR (125 MHz, $CDCl_3$)

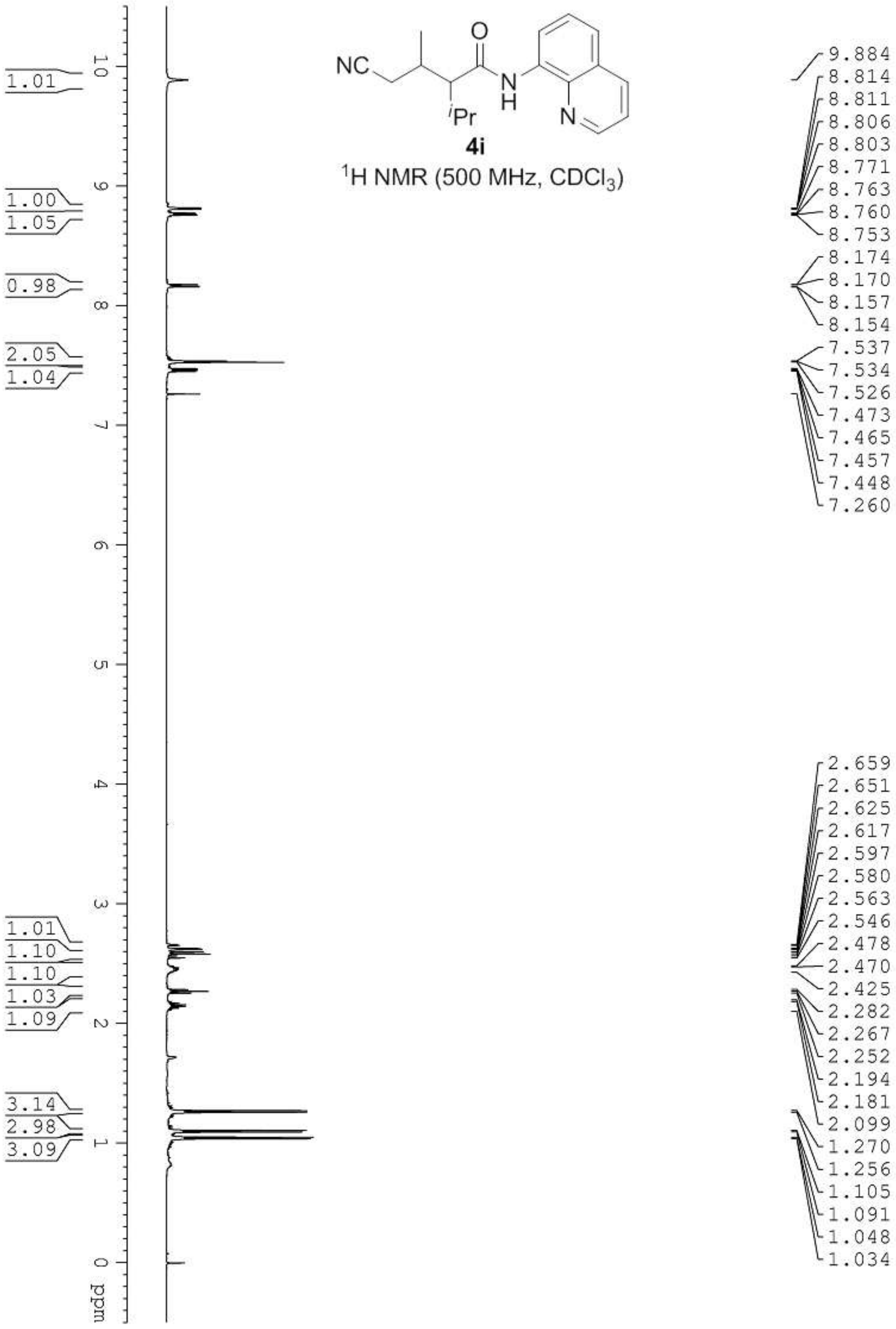
NC
O
N
H
iPr
N
4i
1H NMR (500 MHz, CDCl3)
9.884
8.814
8.811
8.806
8.803
8.771
8.763
8.760
8.753
8.174
8.170
8.157
8.154
7.537
7.534
7.526
7.473
7.465
7.457
7.448
7.260
2.659
2.651
2.625
2.617
2.597
2.580
2.563
2.546
2.478
2.470
2.425
2.282
2.267
2.252
2.194
2.181
2.099
1.270
1.256
1.105
1.091
1.048
1.034
1.01
1.00
1.05
0.98
2.05
1.04
1.01
1.10
1.10
1.03
1.09
3.14
2.98
3.09
10
9
8
7
6
5
4
3
2
1
0
ppm

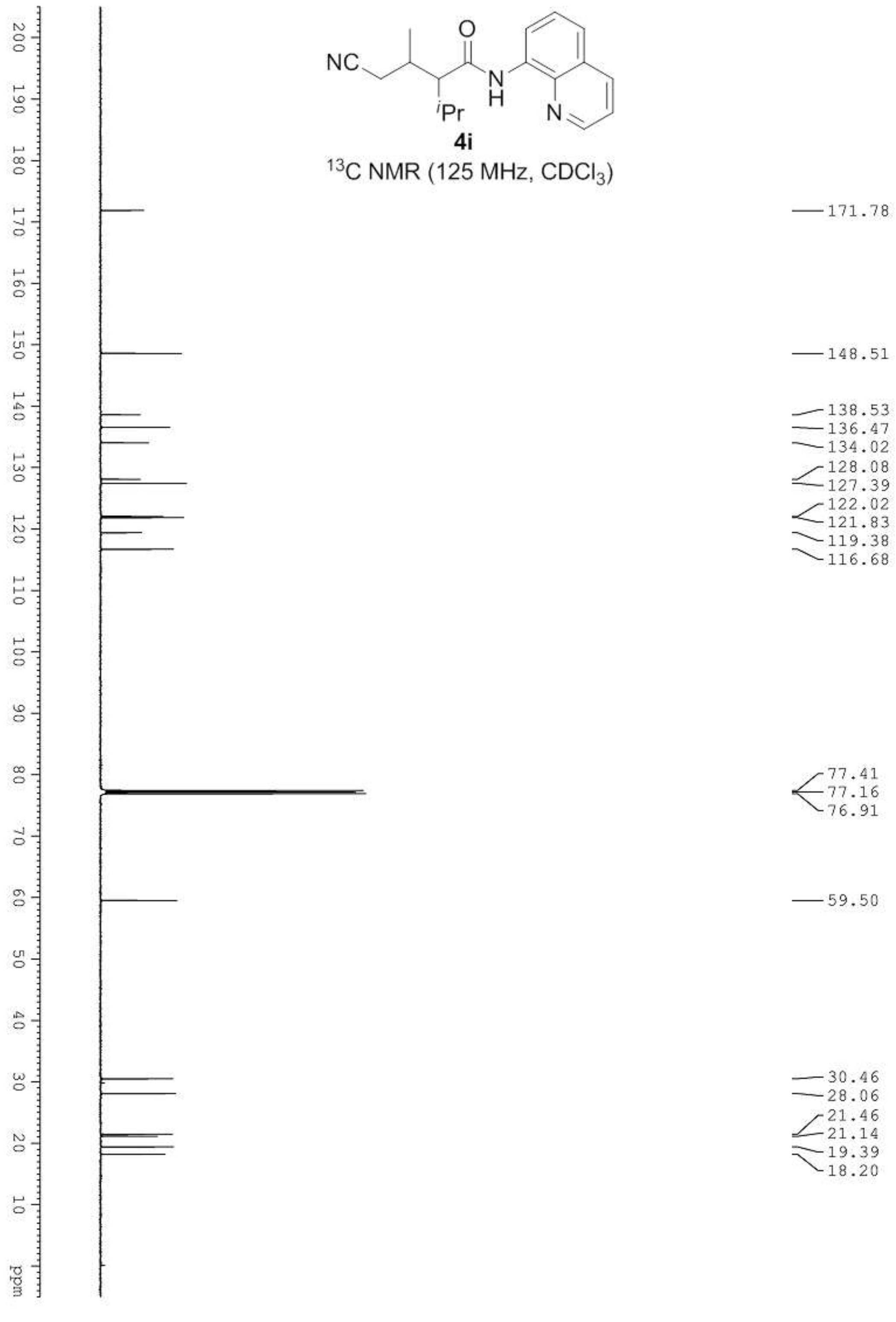
NC
O
N
H
iPr
N
4i
13C NMR (125 MHz, CDCl3)
171.78
148.51
138.53
136.47
134.02
128.08
127.39
122.02
121.83
119.38
116.68
77.41
77.16
76.91
59.50
30.46
28.06
21.46
21.14
19.39
18.20
200
190
180
170
160
150
140
130
120
110
100
90
80
70
60
50
40
30
20
10
ppm

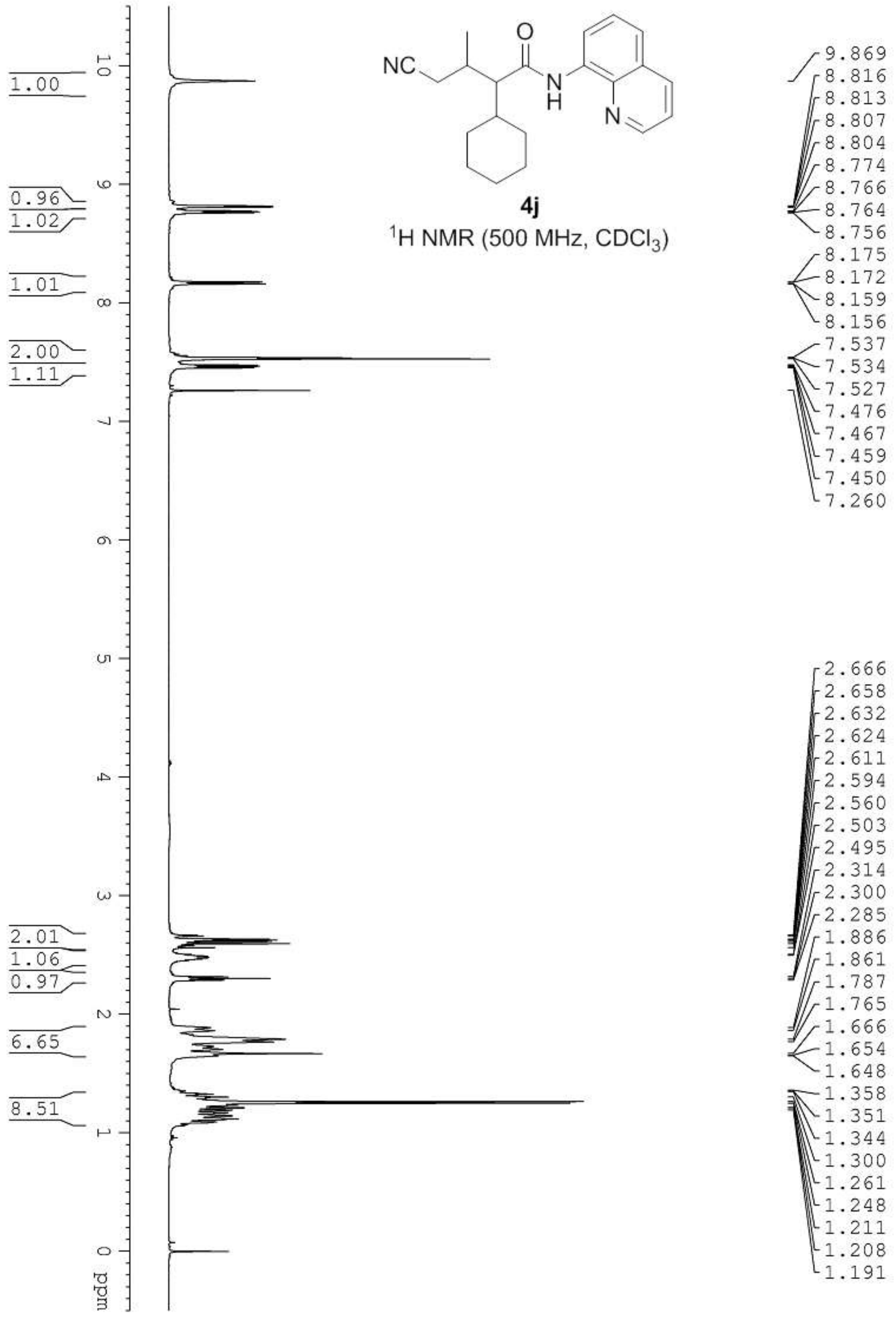

NC
O
N
H
N
4j
1H NMR (500 MHz, CDCl3)
9.869
8.816
8.813
8.807
8.804
8.774
8.766
8.764
8.756
8.175
8.172
8.159
8.156
7.537
7.534
7.527
7.476
7.467
7.459
7.450
7.260
2.666
2.658
2.632
2.624
2.611
2.594
2.560
2.503
2.495
2.314
2.300
2.285
1.886
1.861
1.787
1.765
1.666
1.654
1.648
1.358
1.351
1.344
1.300
1.261
1.248
1.211
1.208
1.191
1.00
0.96
1.02
1.01
2.00
1.11
2.01
1.06
0.97
6.65
8.51
ppm

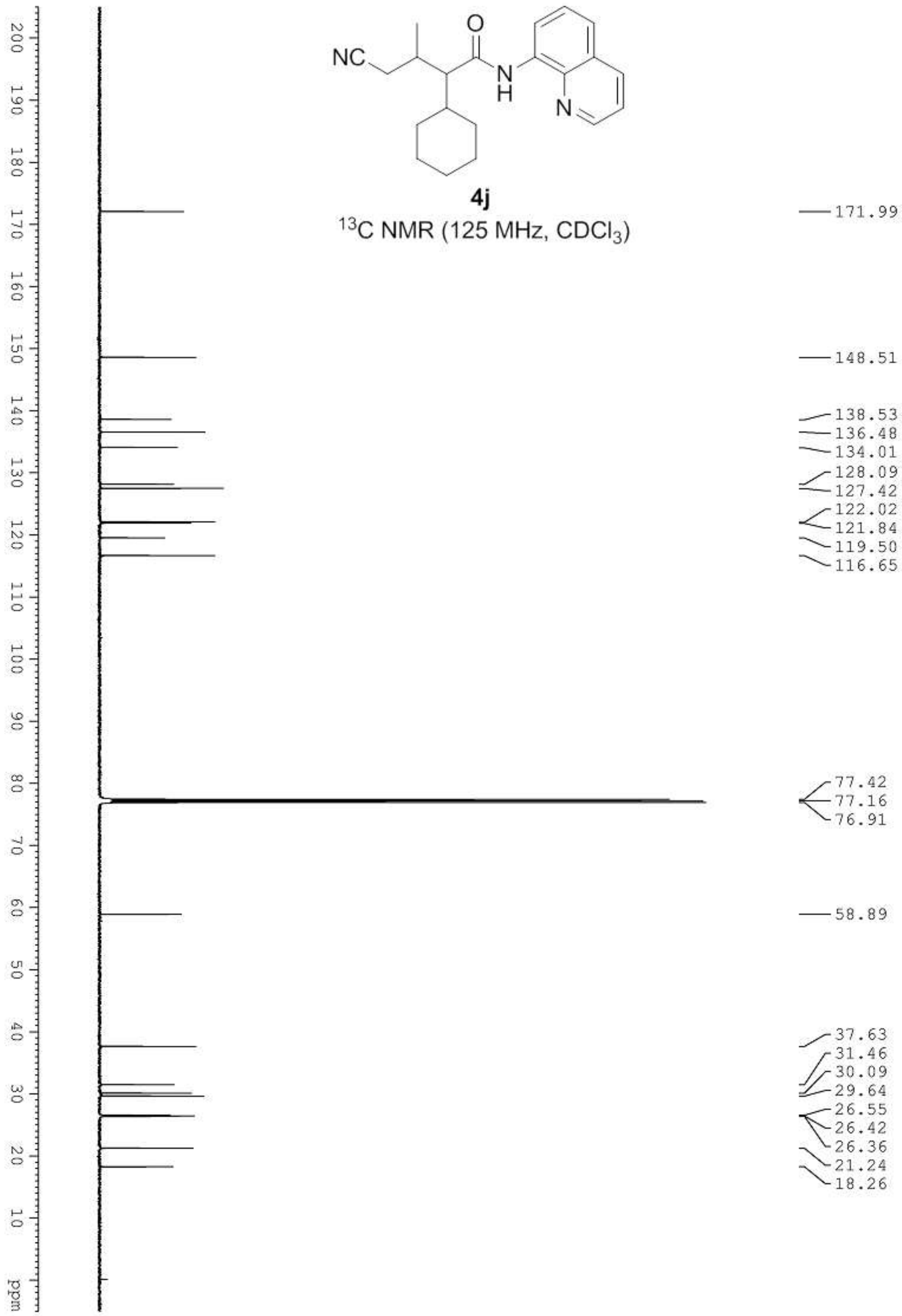

4j

^{13}C NMR (125 MHz, $CDCl_3$)

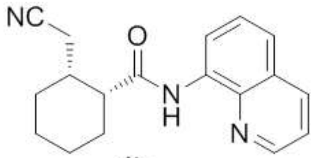

4k

^{1}H NMR (500 MHz, $CDCl_3$)

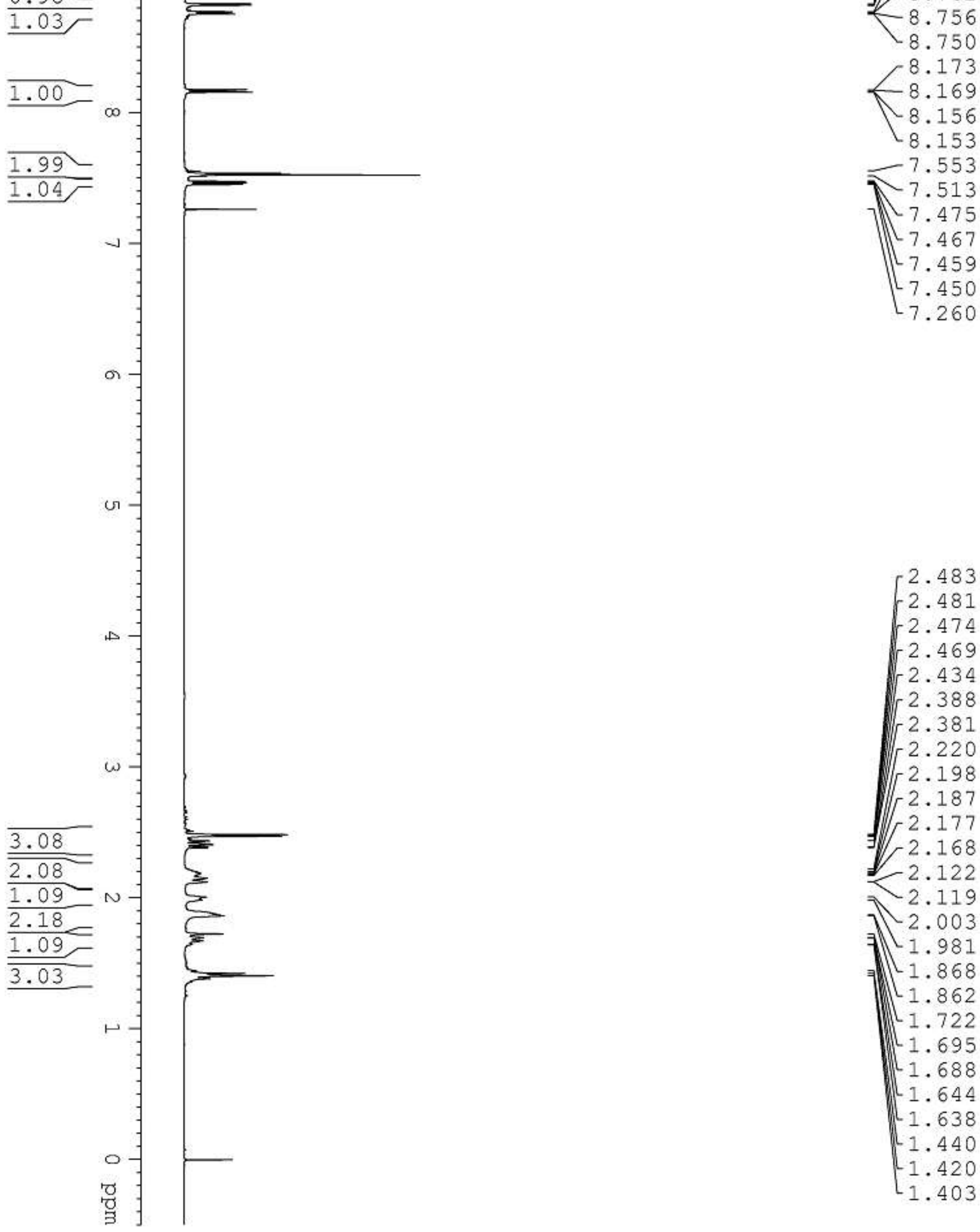

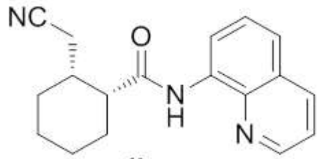

4k

^{13}C NMR (125 MHz, $CDCl_3$)

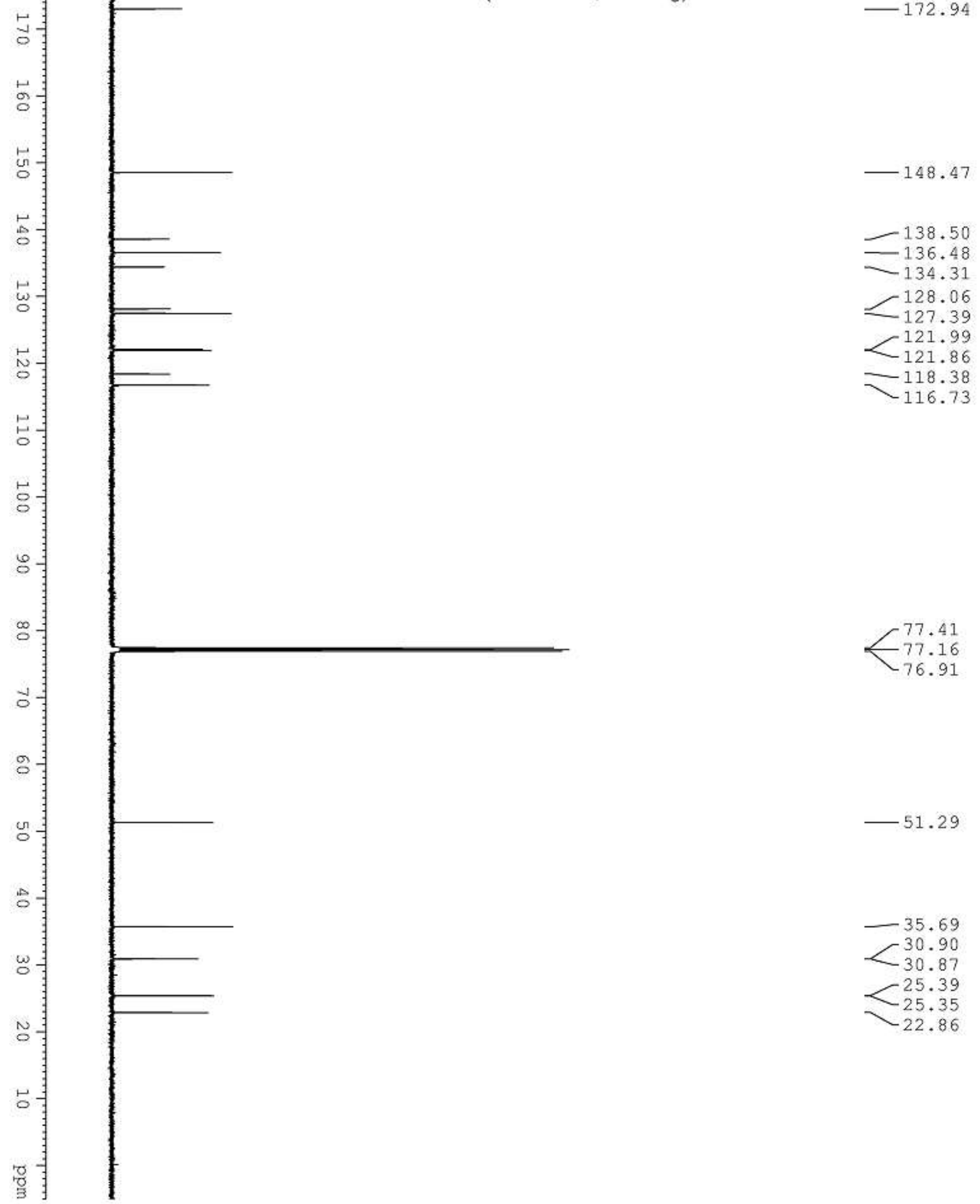

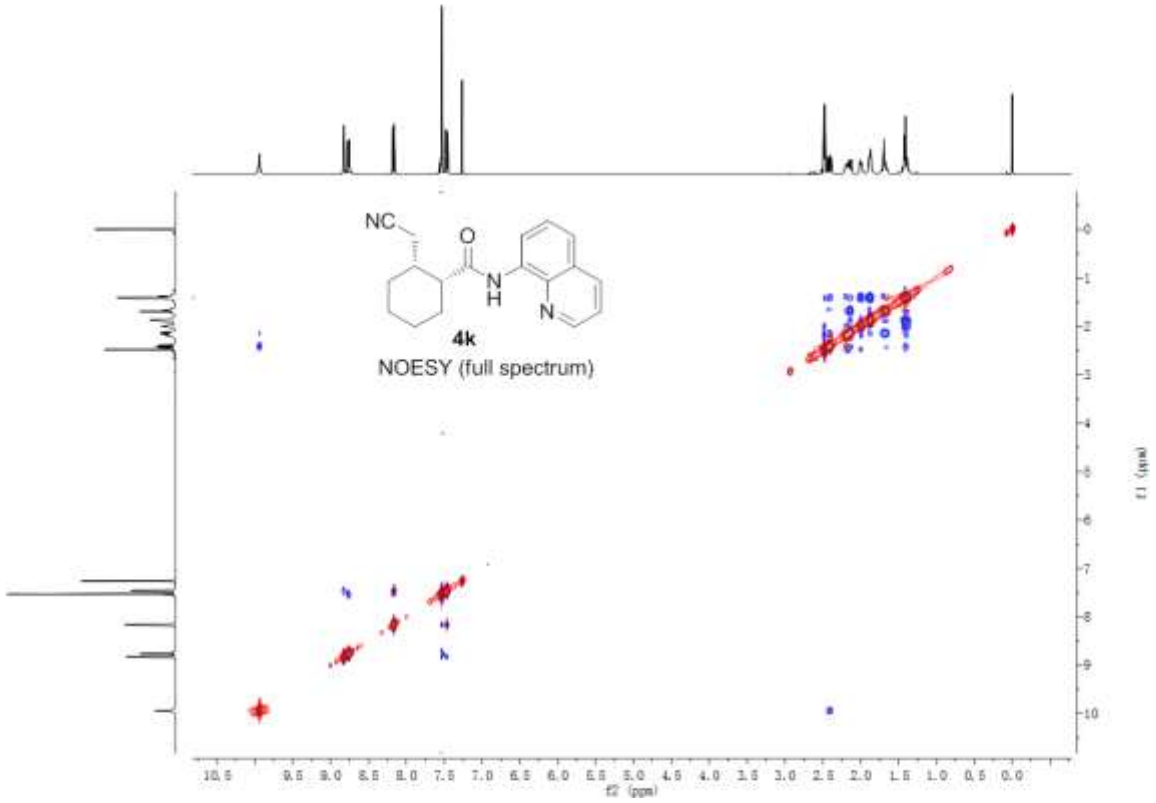
NC
O
N
H
N
4k
NOESY (full spectrum)
f2 (ppm)
f1 (ppm)

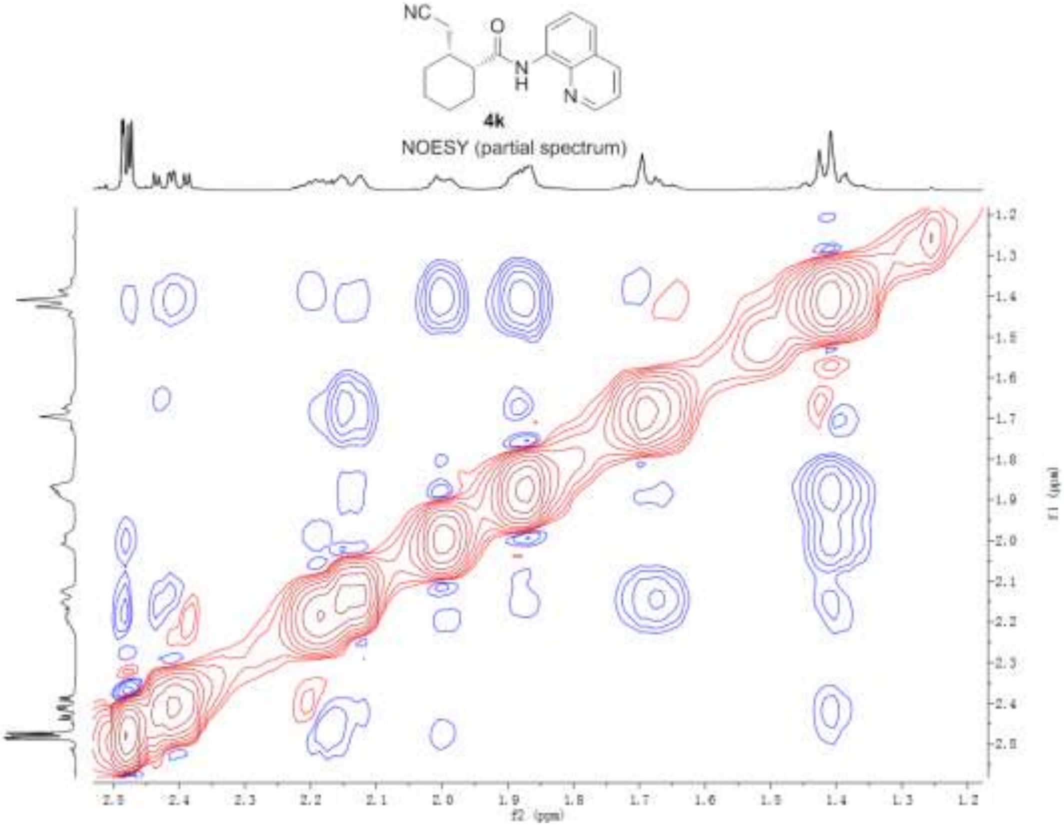
NC
O
N
H
N
4k
NOESY (partial spectrum)
f2 (ppm)
f1 (ppm)

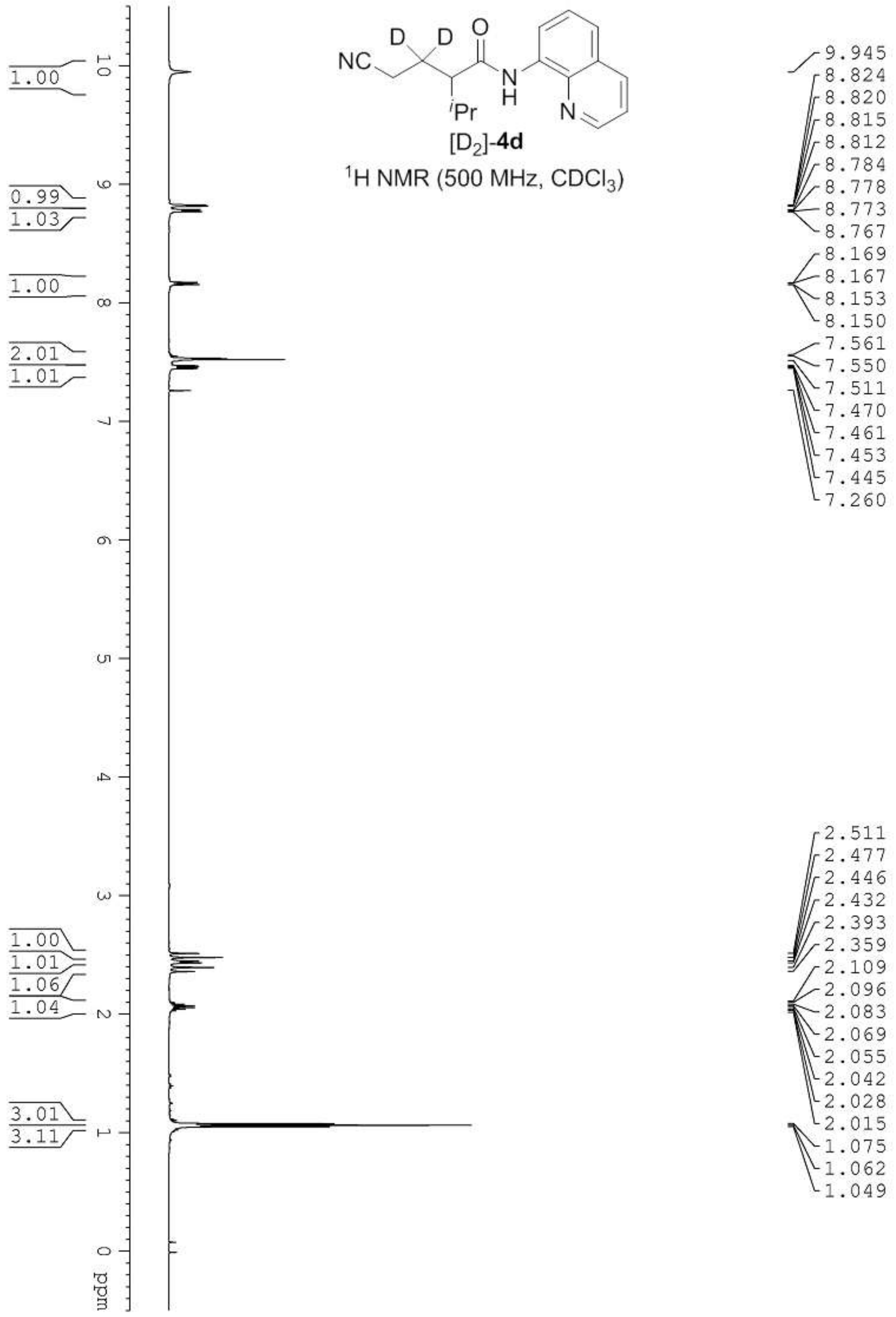

[D_2]-**4d**

^{1}H NMR (500 MHz, $CDCl_3$)

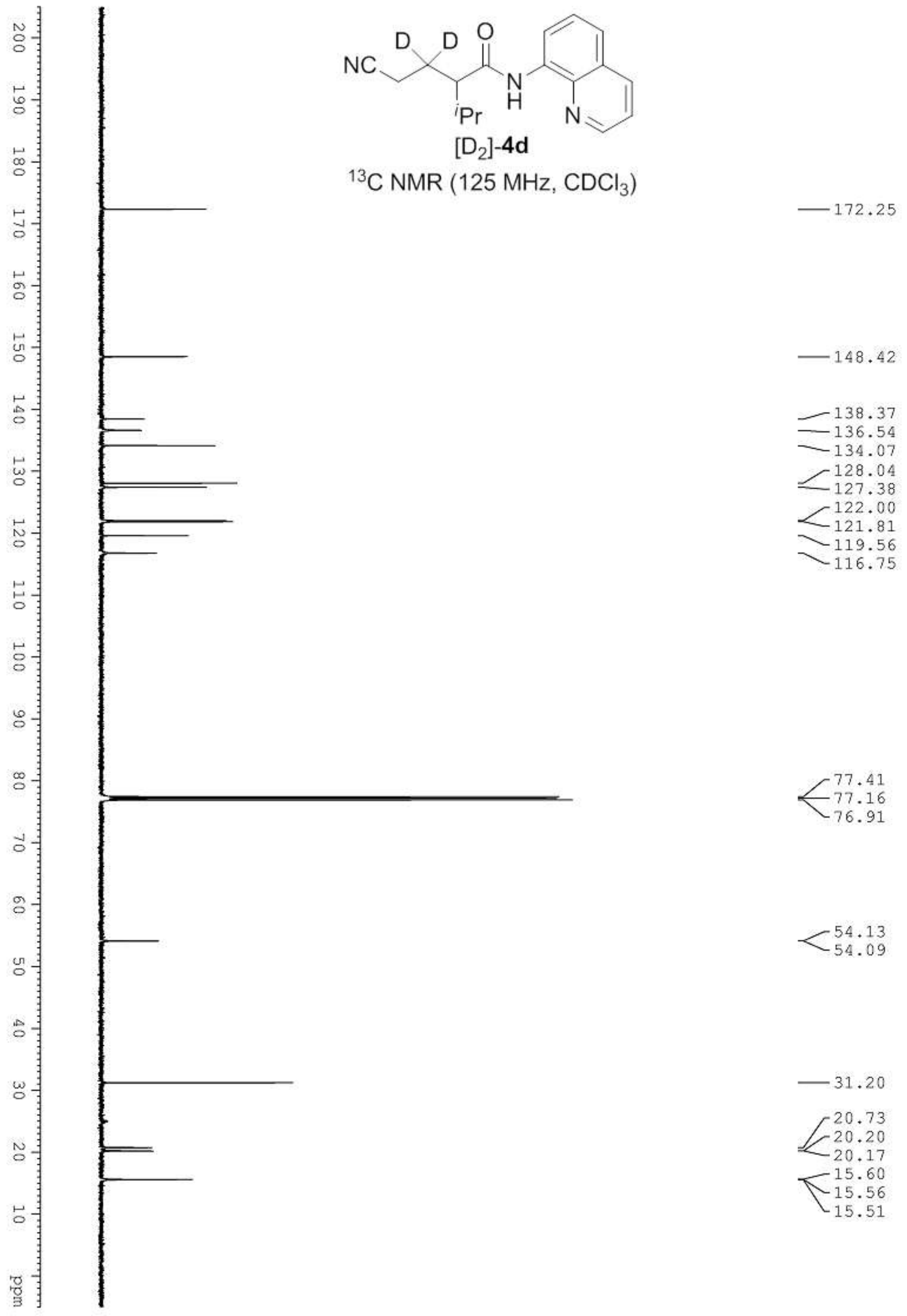
NC
D
D
O
N
H
N
iPr
[D2]-4d
13C NMR (125 MHz, CDCl3)
172.25
148.42
138.37
136.54
134.07
128.04
127.38
122.00
121.81
119.56
116.75
77.41
77.16
76.91
54.13
54.09
31.20
20.73
20.20
20.17
15.60
15.56
15.51
200
190
180
170
160
150
140
130
120
110
100
90
80
70
60
50
40
30
20
10
ppm

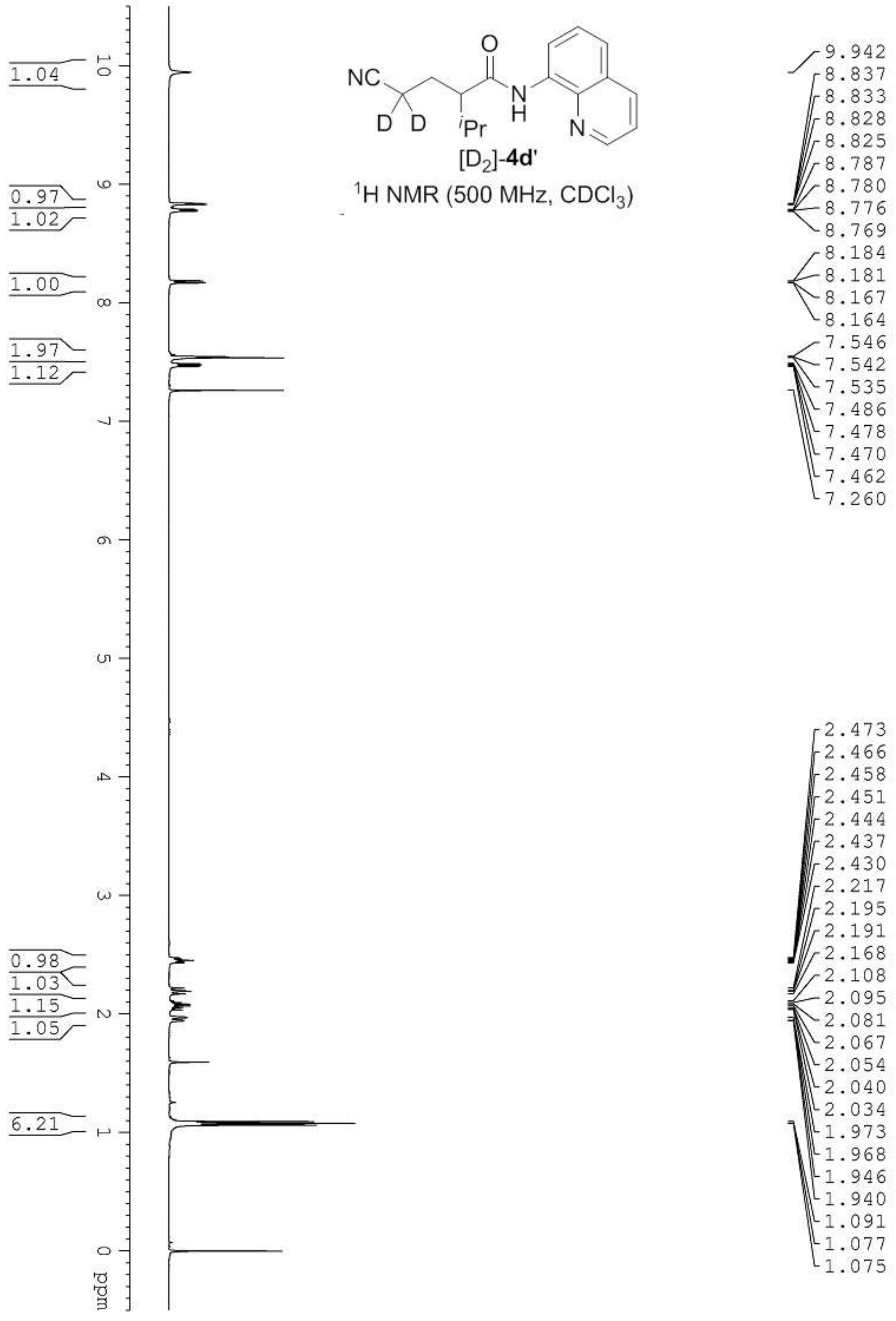
NC
D D
iPr
O
N
H
N
[D2]-4d'
1H NMR (500 MHz, CDCl3)
9.942
8.837
8.833
8.828
8.825
8.787
8.780
8.776
8.769
8.184
8.181
8.167
8.164
7.546
7.542
7.535
7.486
7.478
7.470
7.462
7.260
2.473
2.466
2.458
2.451
2.444
2.437
2.430
2.217
2.195
2.191
2.168
2.108
2.095
2.081
2.067
2.054
2.040
2.034
1.973
1.968
1.946
1.940
1.091
1.077
1.075
1.04
0.97
1.02
1.00
1.97
1.12
0.98
1.03
1.15
1.05
6.21
10
9
8
7
6
5
4
3
2
1
0
ppm

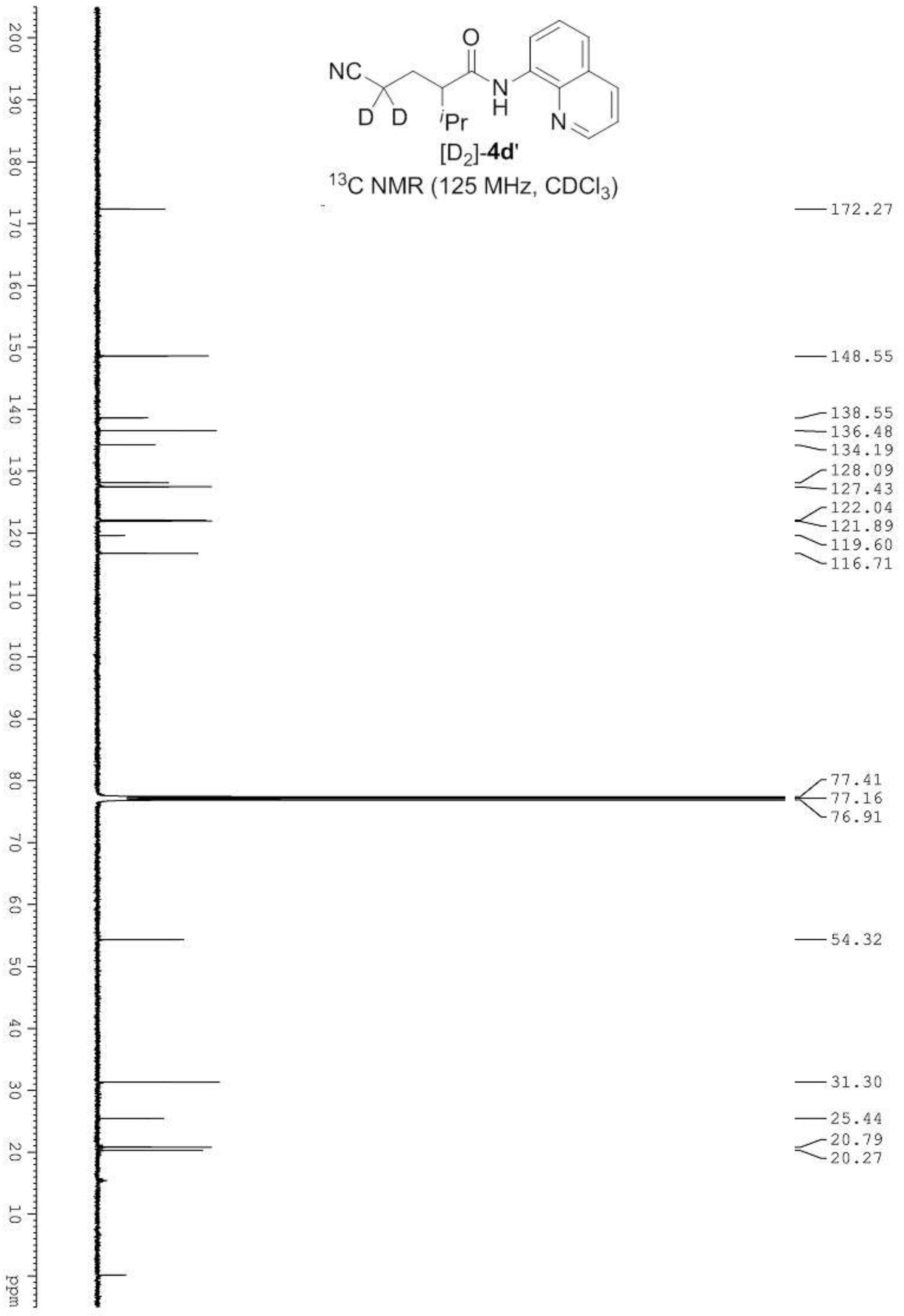
NC
D D
iPr
O
N
H
N
[D2]-4d'
13C NMR (125 MHz, CDCl3)
172.27
148.55
138.55
136.48
134.19
128.09
127.43
122.04
121.89
119.60
116.71
77.41
77.16
76.91
54.32
31.30
25.44
20.79
20.27
200
190
180
170
160
150
140
130
120
110
100
90
80
70
60
50
40
30
20
10
ppm

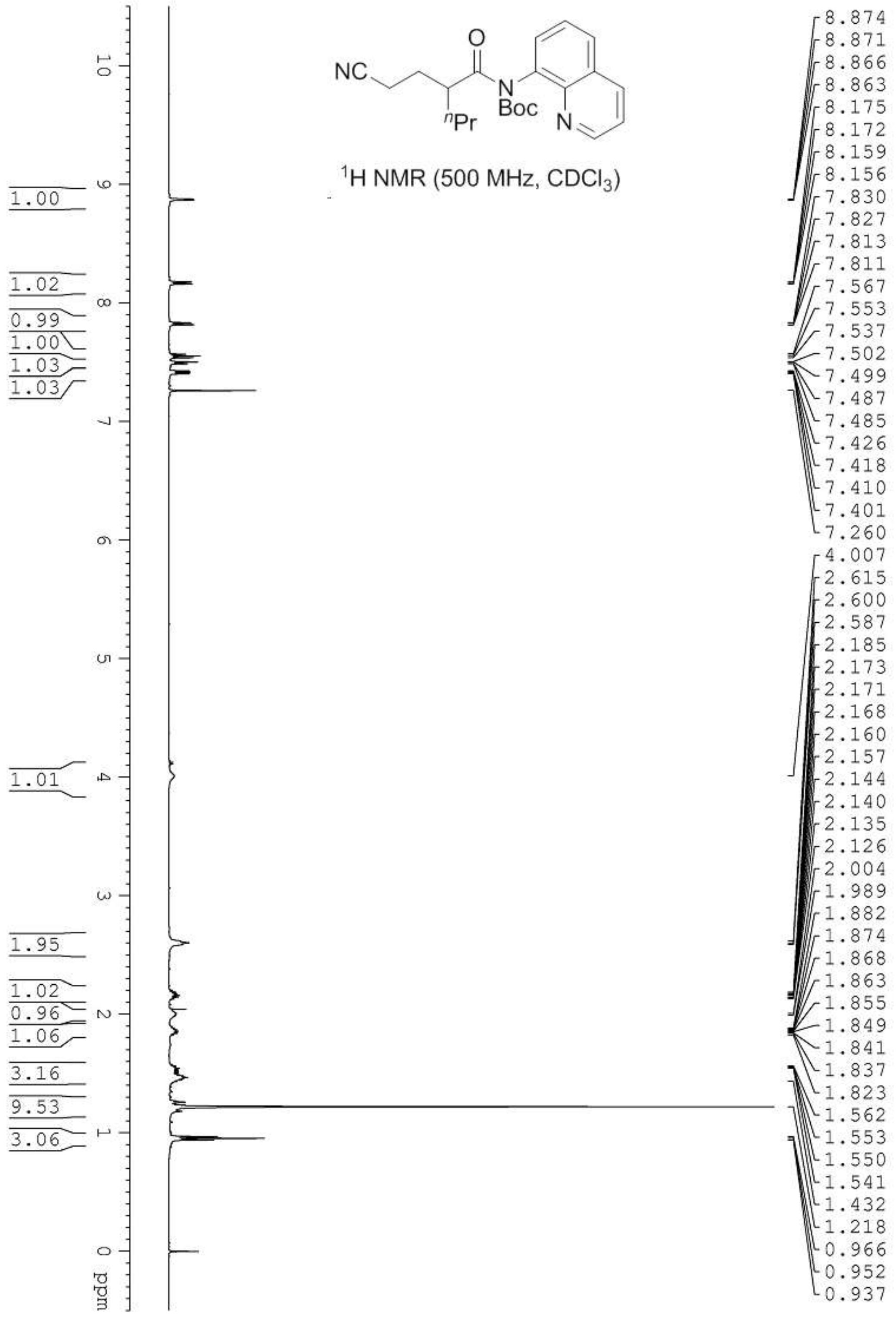
NC
O
N
Boc
nPr
N
1H NMR (500 MHz, CDCl3)
8.874
8.871
8.866
8.863
8.175
8.172
8.159
8.156
7.830
7.827
7.813
7.811
7.567
7.553
7.537
7.502
7.499
7.487
7.485
7.426
7.418
7.410
7.401
7.260
4.007
2.615
2.600
2.587
2.185
2.173
2.171
2.168
2.160
2.157
2.144
2.140
2.135
2.126
2.004
1.989
1.882
1.874
1.868
1.863
1.855
1.849
1.841
1.837
1.823
1.562
1.553
1.550
1.541
1.432
1.218
0.966
0.952
0.937
1.00
1.02
0.99
1.00
1.03
1.03
1.01
1.95
1.02
0.96
1.06
3.16
9.53
3.06
ppm

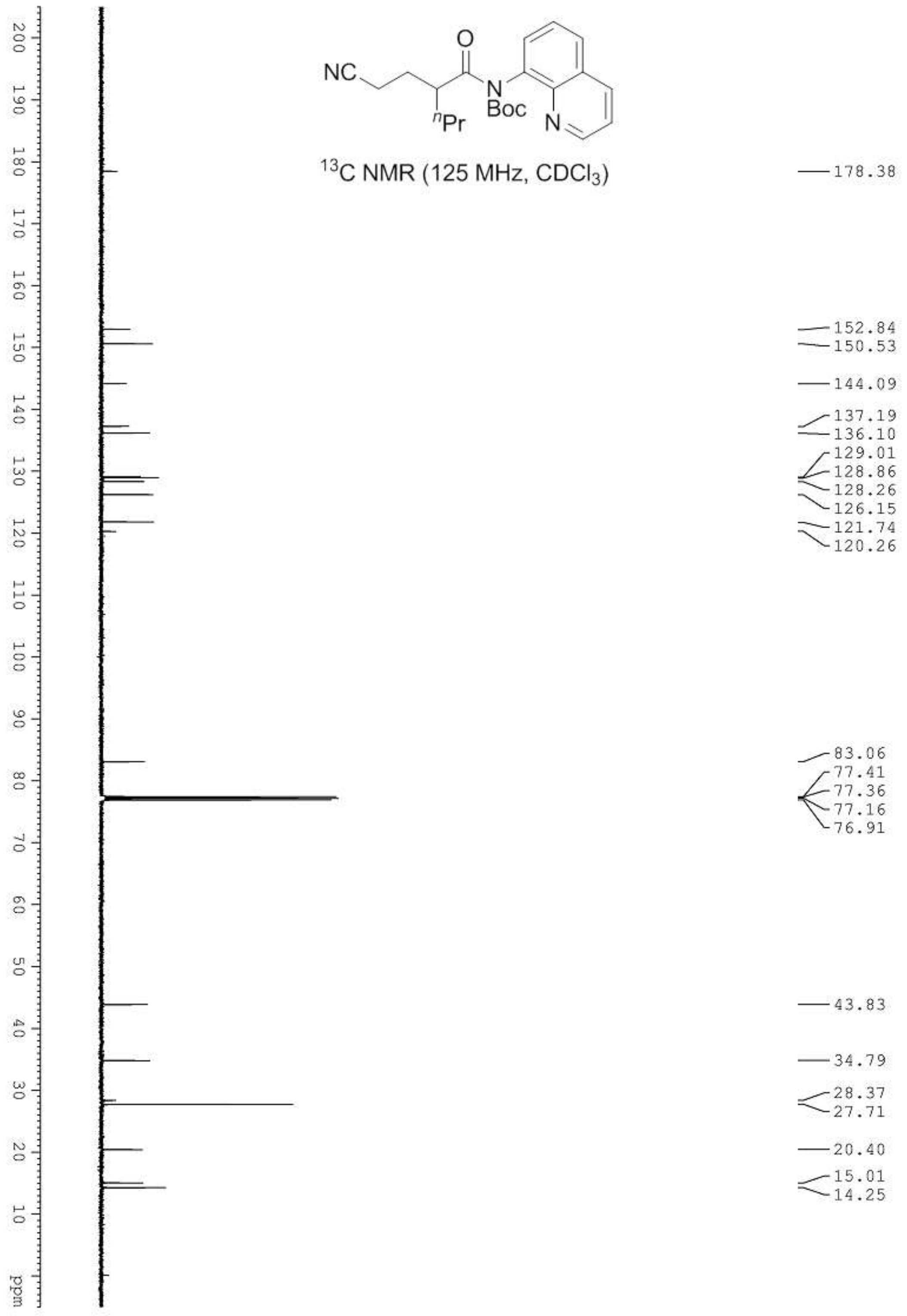
NC
O
N
Boc
N
nPr
13C NMR (125 MHz, CDCl3)
178.38
152.84
150.53
144.09
137.19
136.10
129.01
128.86
128.26
126.15
121.74
120.26
83.06
77.41
77.36
77.16
76.91
43.83
34.79
28.37
27.71
20.40
15.01
14.25
200
190
180
170
160
150
140
130
120
110
100
90
80
70
60
50
40
30
20
10
ppm

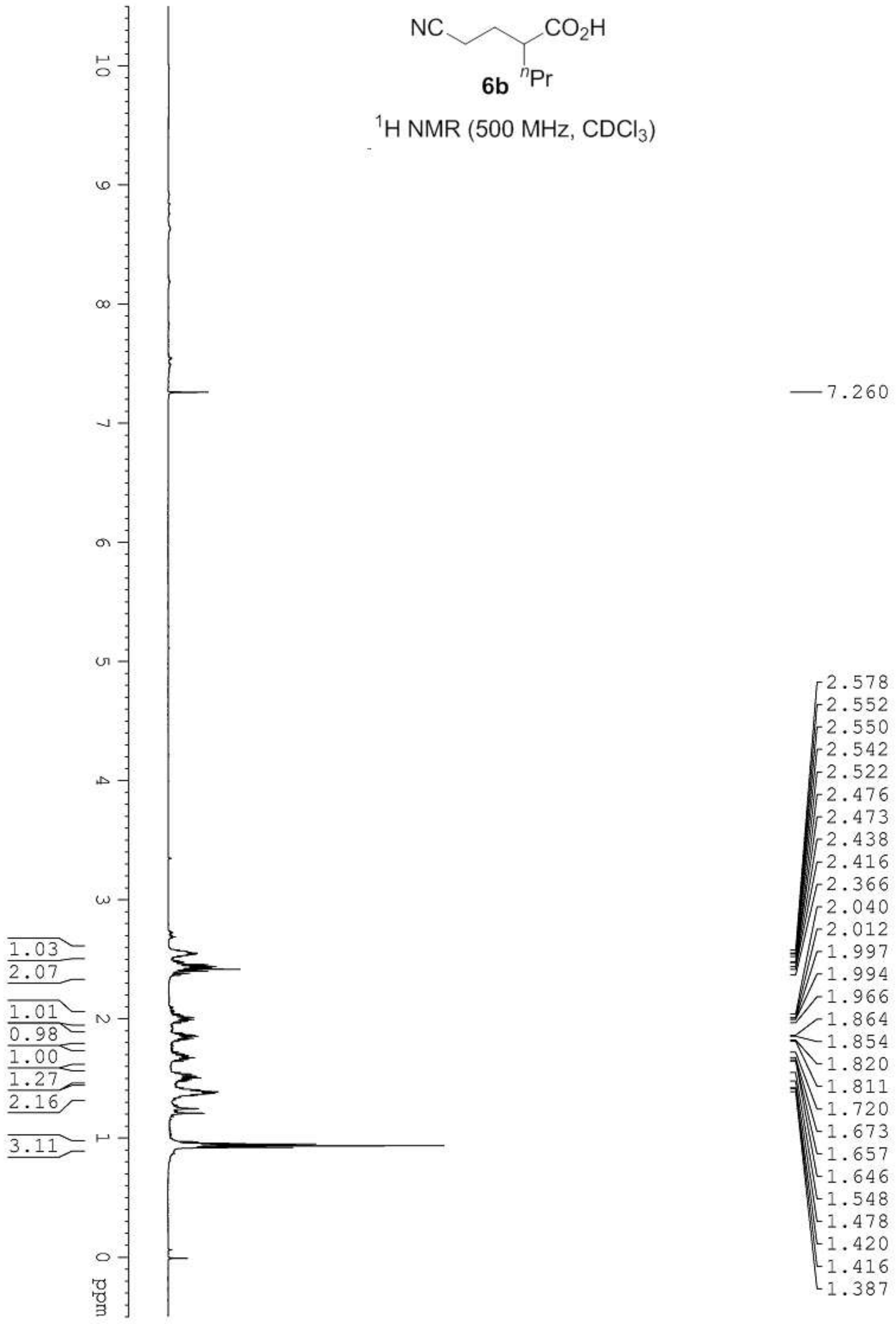
NC
CO2H
6b
nPr
1H NMR (500 MHz, CDCl3)
7.260
2.578
2.552
2.550
2.542
2.522
2.476
2.473
2.438
2.416
2.366
2.040
2.012
1.997
1.994
1.966
1.864
1.854
1.820
1.811
1.720
1.673
1.657
1.646
1.548
1.478
1.420
1.416
1.387
1.03
2.07
1.01
0.98
1.00
1.27
2.16
3.11
10
9
8
7
6
5
4
3
2
1
0
ppm

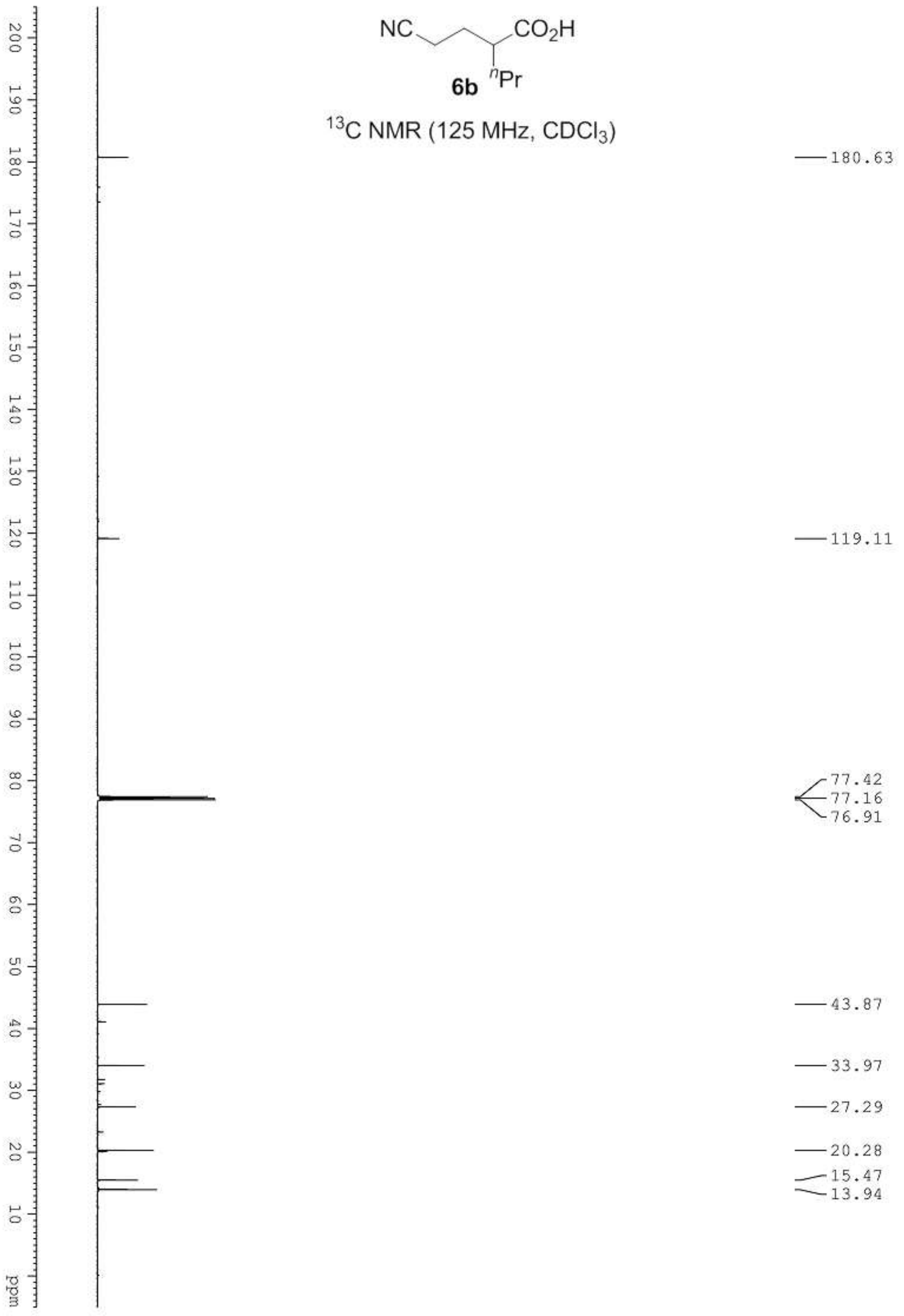

6b

^{13}C NMR (125 MHz, $CDCl_3$)
